# Genome-Wide Identification of Reverse Complementary microRNA Genes in Plants

**DOI:** 10.1371/journal.pone.0046991

**Published:** 2012-10-23

**Authors:** Chaogang Shao, Xiaoxia Ma, Xiufang Xu, Huizhong Wang, Yijun Meng

**Affiliations:** 1 College of Life Sciences, Huzhou Teachers College, Huzhou, The People’s Republic of China; 2 College of Life and Environmental Sciences, Hangzhou Normal University, Hangzhou, The People’s Republic of China; East Carolina University, United States of America

## Abstract

MicroRNAs (miRNAs) are ∼21-nucleotide small RNAs (sRNAs) with essential regulatory roles in plants. They are generated from stem-loop-structured precursors through two sequential Dicer-like 1 (DCL1)-mediated cleavages. To date, hundreds of plant miRNAs have been uncovered. However, the question, whether the sequences reverse complementary (RC) to the miRNA precursors could form hairpin-like structures and produce sRNA duplexes similar to the miRNA/miRNA* pairs has not been solved yet. Here, we interrogated this possibility in 16 plant species based on sRNA high-throughput sequencing data and secondary structure prediction. A total of 59 RC sequences with great potential to form stem-loop structures and generate miRNA/miRNA*-like duplexes were identified in ten plants, which were named as RC-miRNA precursors. Unlike the canonical miRNAs, only a few cleavage targets of the RC-miRNAs were identified in *Arabidopsis* (*Arabidopsis thaliana*) and rice (*Oryza sativa*), and none in Soybean (*Glycine max*) based on degradome data. Surprisingly, the genomic regions surrounding some of the RC-miRNA target recognition sites were observed to be specifically methylated in both *Arabidopsis* and rice. Taken together, we reported a new class of miRNAs, called RC-miRNAs, which were generated from the antisense strands of the miRNA precursors. Based on the results, we speculated that the mature RC-miRNAs might have subtle regulatory activity through target cleavages, but might possess short interfering RNA-like activity by guiding sequence-specific DNA methylation.

## Introduction

MicroRNAs (miRNAs) are small non-coding molecules discovered at the end of the last century, and their critical regulatory roles in numerous biological processes in organisms were widely recognized at the beginning of this century [1]–[4]. In recent years, the newly developed high-throughput sequencing (HTS; also called next-generation sequencing) technology was employed for transcriptome-wide small RNA (sRNA) profiling [5], [6]. The sequencing results provided us with an unprecedented sRNA universe containing miRNAs and other sRNA species such as short interfering RNAs (siRNAs). However, a problem was subsequently encountered that how to predict and validate new miRNA genes based on such huge HTS data sets. The criteria for miRNA definition were proposed for both general organisms and plant species specifically [7], [8]. Although it is still difficult to guarantee the high accuracy of miRNA annotations based on these criteria, the major part of the settled principle was considered to be indispensable for miRNA definition. For plants, the miRNA precursors should be able to form compelling stem-loop structures, and the mature miRNAs should be easily detectable by HTS or other fine-scale experimental methods such as Northern blot. Ideally, both miRNA and the corresponding miRNA* should be cloned. And, when mapped to the predicted hairpin-structured precursor, they should form a short duplex with 2-nucleotide (nt) 3′ overhangs at both ends, which result from two sequential cleavages of Dicer-like 1 (DCL1) in the nucleus [3], [4]. Besides, miRNAs are sorted into Argonaute 1 (AGO1)-associated miRNA-induced silencing complexes (miRISCs) in most cases. Based on these well-established notions, the structure- and accumulation-based approach was widely adopted for new miRNA gene identification.

To date, hundreds of miRNAs belonging to various plant species have been registered in the famous miRNA database, miRBase (http://www.mirbase.org/index.shtml) [9], and more and more novel miRNA genes are being discovered. However, one question seems to be obvious that whether the sequences reverse complementary (RC) to the miRNA precursors could generate miRNA-like sRNAs since these RC sequences tend to form stem-loop structures more easily compared to the random genomic sequences. Supporting this scenario, a few solid evidences were found in animals. Almost at the same time, three groups reported the discovery of a pair of sense/antisense miRNA genes, *mir-iab-4* and *mir-iab-8* [10]–[12]. Both non-coding transcripts transcribed from the two miRNA genes could form canonical hairpin structures, and the mature miRNAs generated from both precursors (mir-iab-4-5p/-3p and mir-iab-8-5p/-3p) could be detected by Northern analysis. Furthermore, Stark *et al.* (2008) and Tyler *et al.* (2008) also indicated that transcription and production of functional miRNAs from the antisense strands of the annotated miRNA genes could be widespread in both invertebrates and vertebrates. In plants, however, there is no exact hint supporting the antisense transcription of miRNA genes. Instead, only a few related cases were reported. A genome-wide identification of rice miRNAs carried out by Lu and his colleagues (2008) revealed a new class of miRNAs, natural antisense microRNAs (nat-miRNAs). Different from the antisense miRNA genes identified in animals, nat-miRNAs are produced from the transcripts antisense to protein-coding genes such as MADS box genes. Although the processing of mature miRNAs from their precursors are also DCL1-dependent, a distinguishable feature of these miRNA genes is the existence of introns resided within their primary transcripts, which must be removed for subsequent hairpin formation [13].

To our best knowledge, no systemic identification of the plant antisense miRNA genes has been performed. To address this issue, we carried out a genome-wide survey of the potential miRNA genes antisense to the miRNA loci registered in miRBase (Release 17) [9]. Sixteen plant species with available sRNA HTS data sets were investigated. After HTS data- and structure-based search and filtering, 59 RC sequences with great potential to form hairpin structures and produce miRNA-like sRNA molecules were identified in ten plant species, which were defined as RC-miRNA genes in order to make them distinguishable to the previously reported natural antisense miRNAs [13]. Similar to the canonical miRNAs, the sequence length of the identified mature RC-miRNAs is highly enriched from 20 to 22 nt. However, different from the miRNAs predominantly starting with 5′ U (uridine) [4], large portions of the RC-miRNAs begin with 5′ A (adenosine) and C (cytosine). The mature RC-miRNAs with high accumulation levels in *Arabidopsis* (*Arabidopsis thaliana*), rice (*Oryza sativa*) and soybean (*Glycine max*) were selected for further functional analyses. Only a few targets were identified in *Arabidopsis* and rice by using degradome sequencing data. Intriguingly, a large portion of these RC-miRNAs were indicated to mediate target site-specific DNA methylation in both plants. On the other hand, several investigated RC-miRNAs are not enriched in AGO1, but in *Arabidopsis* AGO2 and rice AGO4 clade proteins instead. Taken together, we reported a novel class of miRNAs transcribed from the opposite strands of the currently annotated miRNA genes in the plant kingdom. The potential involvement of these RC-miRNAs in the target-specific DNA methylation pathway needs further investigations.

## Results

### Accumulation- and Structure-based Identification of RC-miRNAs

All the miRBase-registered (release 17) miRNA precursors belonging to 16 different plant species (Arabidopsis lyrata, Arabidopsis thaliana, Carica papaya, Citrus sinensis, Glycine max, Gossypium arboretum, Gossypium hirsutum, Hordeum vulgare, Medicago truncatula, Oryza sativa, Populus trichocarpa, Solanum lycopersicum, Sorghum bicolor, Triticum aestivum, Vitis vinifera and Zea mays) with publicly available sRNA HTS data were included to generate RC sequences. All the sRNA HTS short sequences were mapped to the RC sequences of each plant species, and the perfectly matched sequences were retained to generate sRNA distribution patterns for all the RC sequences. To avoid the interference of the stem-loop structures capable of generating enormous siRNA populations [14], [15], and to obtain RC-miRNA precursor candidates with simplified sRNA distribution patterns, the RC sequences with continuously distributed short sequences were discarded, and only the ones with two or more isolated sRNA clusters were retained. According to the recently proposed principle for HTS-based miRNA identification [16], these retained RC sequences were further filtered by the criterion that two or more short sequences with identical 5′ ends should appear in at least one isolated sRNA cluster on each RC sequence. These isolated sRNA clusters served as the database for further identification of mature RC-miRNA candidates (see details in **Materials and Methods**).

**Figure 1:**
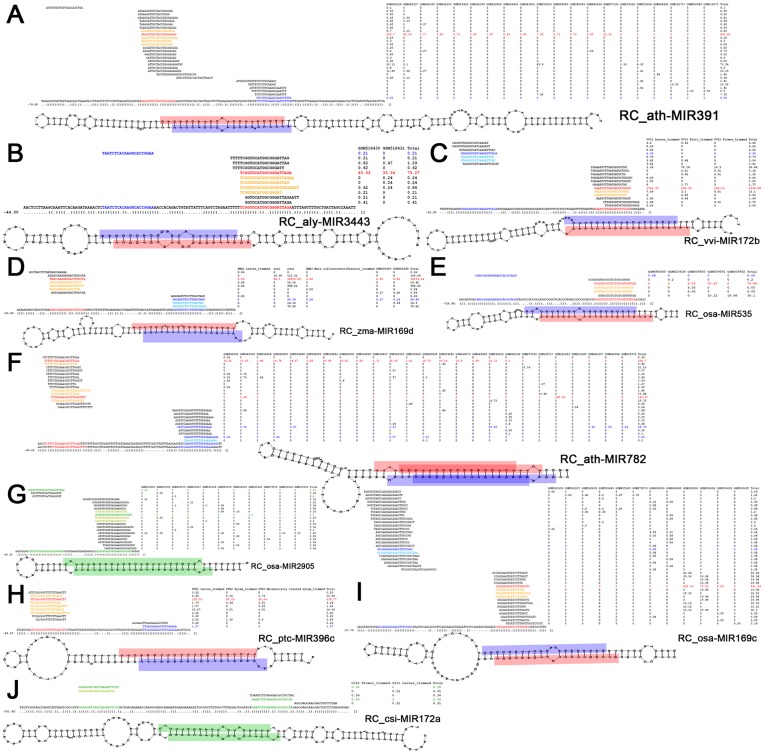
High-throughput sequencing (HTS) data- and structure-based identification of reverse complementary microRNAs (RC-miRNAs). (A) RC-ath-MIR391. (B) RC-aly-MIR3443. (C) RC-vvi-MIR172b. (D) RC-zma-MIR169d. (E) RC-osa-MIR535. (F) RC-ath-MIR782. (G) RC-osa-MIR2905. (H) RC-ptc-MIR396c. (I) RC-osa-MIR169c. (J) RC-csi-MIR172a. For all the panels, the short sequences perfectly mapped to the RC-miRNA precursors along with their normalized read counts in RPM (reads per million) are shown (see Table S1 for the small RNA HTS data sources and see **Materials and Methods** for read count normalization). The mature RC-miRNAs with significantly higher accumulation levels compared to the RC-miRNA* coordinates were highlighted in dark red color, and the other short sequences with identical 5′ ends supporting the RC-miRNAs were in yellow. The RC-miRNA*s were in dark blue, and the other supporting sequences in light blue. For the RC precursors generating RC-miRNAs with indistinguishable accumulation levels on both arms, their mature RC-miRNAs were highlighted in green color and named as miRNA-5p (5′ arm) and miRNA-3p (3′ arm), respectively. The corresponding supporting sequences were in light green color. The RC-miRNAs and the star species were also indicated in the stem-loop structures of their precursors. The parenthesis-dot formed secondary structure expression along with the free energy, and the stem-loop structures were all predicted and generated by RNAshapes [17]. aly: *Arabidopsis lyrata*. ath: *Arabidopsis thaliana*. csi: *Citrus sinensis*. osa: *Oryza sativa*. ptc: *Populus trichocarpa*. vvi: *Vitis vinifera*. zma: *Zea mays*.

Then, the filtered RC sequences were subjected to secondary structure-based manual check. Secondary structure prediction was carried out by using RNAshapes [17]. All the predicted results were manually checked, and only the RC sequences capable of forming compelling hairpin structures were retained. The RC-miRNA and RC-miRNA* candidates were selected from the sRNA clusters on the RC sequences (RC-miRNA candidates should be supported by two or more short sequences with identical 5′ ends), and mapped to the hairpin-structured precursors for manual check. Considering the featured structure of miRNA/miRNA* duplex with 2-nt 3′ overhangs [3], [4], only those RC sequences generating RC-miRNA/RC-miRNA* duplexes with 0- to 3-nt 3′ overhangs were finally considered to be the RC-miRNA precursor candidates. As a result, 59 candidates were identified in ten plant species (Figure S1 and Table S3). For most precursor candidates, both RC-miRNAs and RC-miRNA*s were cloned by HTS, and the RC-miRNAs were supported by two or more different short sequences (Figure 1 and Figure S1). For a larger portion of the precursors (45 out of the 59 precursors), the accumulation levels of the RC-miRNAs were significantly higher than those of the RC-miRNA*s. For the remaining precursors (14 out of 59), the accumulation levels of the RC-miRNAs generated from the 5′ and the 3′ arms were quite similar, and thus were named as RC-miRNA-5p and RC-miRNA-3p respectively. Interestingly, more than one RC-miRNA/RC-miRNA* pairs were identified on some of the precursors (Figure 1F and Figure S1). Notably, these RC-miRNA/RC-miRNA* pairs generated from the same precursors always largely overlap with each other. This observation may result from the wobble effect of DCL-mediated cleavages.

**Figure 2:**
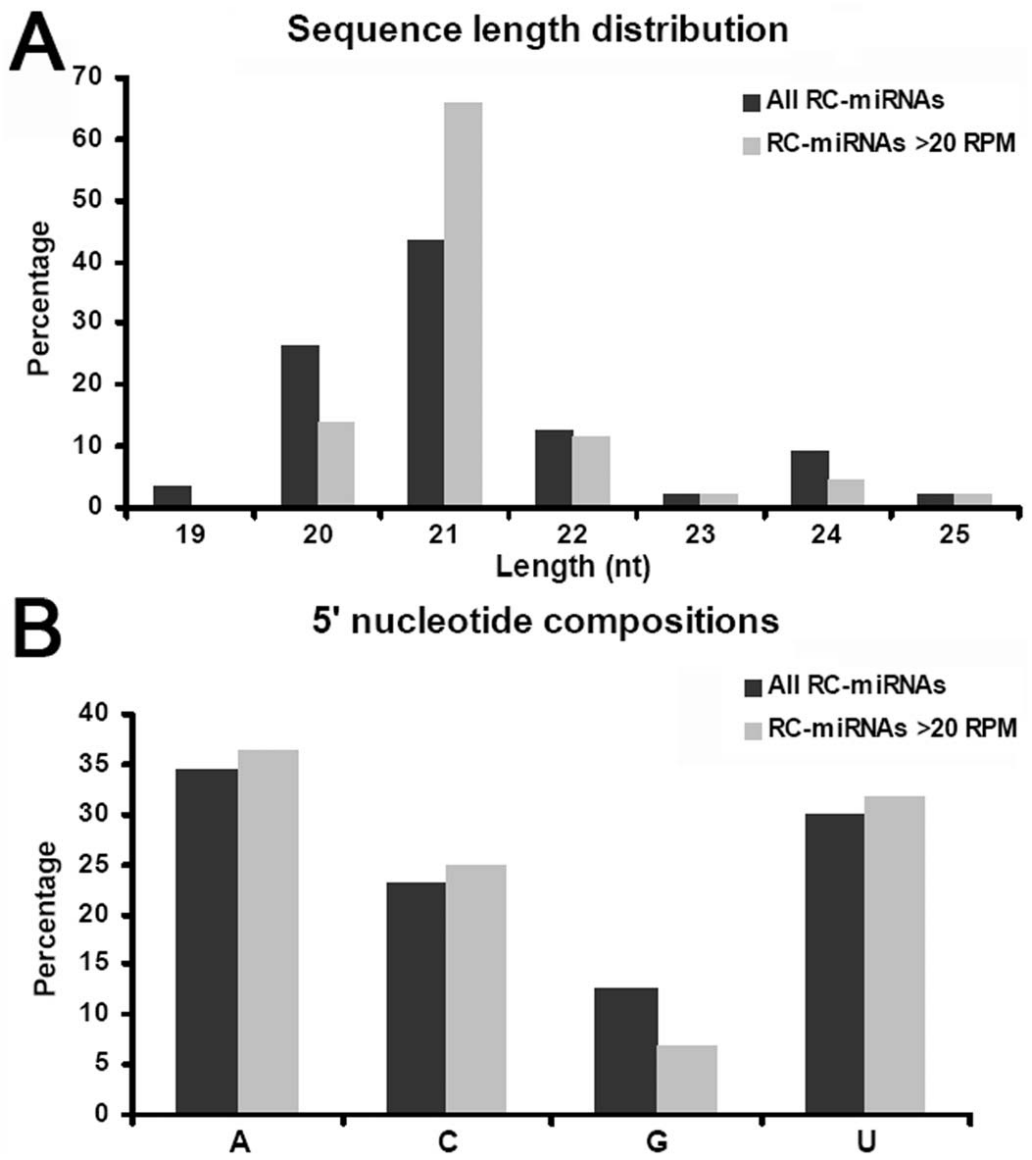
Sequence characteristics of the reverse complementary microRNAs (RC-miRNAs) identified in nine plant species. (A) Sequence length distribution patterns. (B) 5’ terminal nucleotide compositions. For both (A) and (B), all the identified RC-miRNAs (black bars) and the ones with normalized total read counts larger than 20 RPM (reads per million) (gray bars) were analyzed separately.

All the RC-miRNAs (87 sequences) were recruited for sequence feature characterization. The statistical results showed that the sequence length of the RC-miRNAs was enriched from 20 to 22 nt (Figure 2A), which was similar to the plant miRNAs. However, quite different from the miRNAs that predominantly starting with 5′ U [4], nearly equal portions of the RC-miRNAs initiate with A (34.48%), C (22.99%), and U (29.89%) respectively, but much less with 5′ G (guanine; 12.64%) (Figure 2B). Considering the notion that accumulation level is a good indication for the biological relevance of a miRNA, the RC-miRNAs with relatively high accumulation levels based on the HTS data [with normalized total read counts larger than 20 RPM (reads per million)] were analyzed again. The result showed that both sequence length distribution and 5′ nucleotide composition were highly consistent with those of the total RC-miRNA population (see gray bars in Figure 2).

To address the concern whether a random genomic sequence could form a hairpin structure and produce RC-miRNA/RC-miRNA*-like short duplex, we set out to create a control set in both *Arabidopsis* and rice. To make the control sets more comparable to the RC sequences of the miRNA genes, 1,000 sequences antisense to the protein-coding genes were randomly collected in both plants. The length of the random sequences was determined by the average length of the discovered RC-miRNA precursors, i.e. 159 nt in *Arabidopsis* and 132 nt in rice (see sequence information in Data S1 and S2). Then, we set out to identify potential RC-miRNAs by using a similar analytical workflow with slight differences. First, 1,000 random sequences in each plant were subjected to secondary structure prediction by using RNAshapes, and only the antisense sequences capable of forming hairpin structures were retained for the following sRNA HTS data-based analysis. This step greatly reduced the computational time cost during the next step, since only 106 sequences in *Arabidopsis* and 210 in rice passed the first-step filtering (Data S3 and S4). Then, all the sRNA short sequences from the HTS data sets were mapped to the retained random sequences. As a result, only 34 sequences in *Arabidopsis* and 111 sequences in rice possess perfectly-matched sRNAs (Data S5 and S6). Finally, manual inspection was carried out to search for the potential miRNA/miRNA* duplex with 2-nt 3′ overhangs at both ends. Notably, no ideal hairpin structure capable of producing miRNA/miRNA* duplex was discovered. This discovery rate (0/1000) was significantly low compared to those in *Arabidopsis* (5/232) and rice (26/491). Thus, it is reasonable to conclude that the RC-miRNA genes are not randomly distributed on the genomic sequences antisense to the annotated genes.

### Degradome Data-based Target Identification

**Figure 3:**
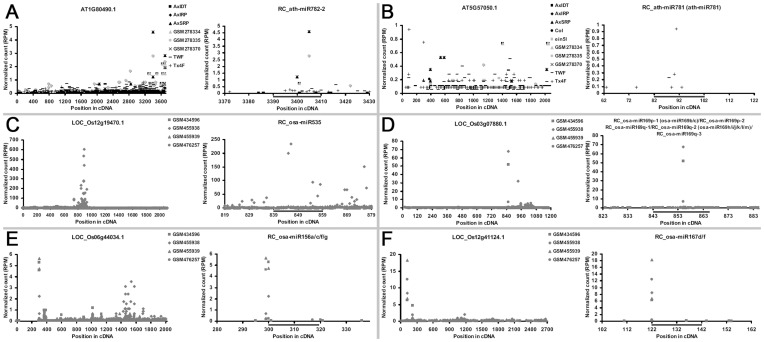
Degradome sequencing data-based identification of the targets of the highly accumulated reverse complementary microRNAs (RC-miRNAs) in *Arabidopsis* and rice. For all the sub-figures (A to F), the left panels depict the degradome signals all along the target transcripts, and the other panels provide detailed views of the cleavage signals within the regions surrounding the target recognition sites (denoted by gray horizontal lines). The transcript IDs are shown in the left panels, and the RC-miRNA names are listed in the other panels. The *x* axes measure the positions of the signals along the transcripts, and the *y* axes measure the signal intensities based on normalized counts (in RPM, reads per million), allowing cross-library comparison. See Table S2 for the degradome data sets used in this analysis.

The canonical plant miRNAs were incorporated into AGO-associated (AGO1 normally) miRISCs to guide the silencing complexes to the target transcripts for cleavage-based post-transcriptional regulation [2]–[4]. Here, the highly accumulated RC-miRNAs (with total read counts more than 20 RPM) of *Arabidopsis*, rice and soybean were recruited for degradome sequencing data-based target identification. First, according to the gene model annotations from TAIR (Release 10) [18], TIGR rice (Release 6.1) [19], and GmGDB belonging to PlantGDB (version Glyma1) [20], transcriptome-wide target prediction was performed for the three plants by using miRU algorithm [21], [22]. Then, the predicted targets were subjected to degradome data-based (see Table S2 for data sources) validation by generating t-plots (target plots) [23], [24] for manual check following the rules proposed previously [25]. Strikingly, only a few targets were confirmed to be cleaved at the target binding sites in *Arabidopsis* and rice (Figure 3, Figure S2 and S3, and Table S5), and none was identified in soybean. For rice, a large portion of the confirmed targets with compelling cleavage signals in the middle of the binding sites were recognized by the mature RC-miRNAs produced from the precursors RC-osa-MIR169p and RC-osa-MIR169q, which share the identical sequences with osa-miR169b/c and osa-miR169h/i/j/k/l/m (Figure 3, Figure S3 and Table S4). The targets were annotated as *Nuclear Factor Y* (*NFY*), which were previously shown to be targeted by miR169 in *Arabidopsis* [26], [27]. However, it is hard to tell that either RC-osa-miR169 or osa-miR169, or both exert their cleavage-based regulatory roles.

### RC-miRNA-guided DNA Methylation through a Target Site-specific Manner

**Figure 4:**
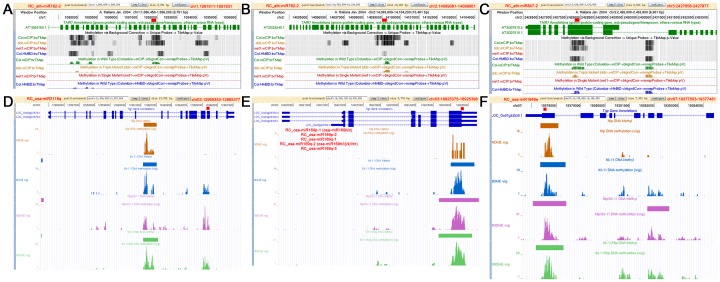
Site-specific DNA methylation mediated by the highly accumulated reverse complementary microRNAs (RC-miRNAs) in *Arabidopsis* and rice. (A) and (B) RC-ath-miR782-2-mediated DNA methylation on the region encoding *AT1G04160* and *AT2G33240*. (C) RC-ath-miR847-2-mediated DNA methylation on the region encoding *AT3G07610*. (D) RC-oas-miR2118q-mediated DNA methylation on the region encoding *LOC_Os03g22570*. (E) RC-oas-miR169p/q-mediated DNA methylation on the region encoding *LOC_Os03g29760*. (F) RC-oas-miR1846e-mediated DNA methylation on the region encoding *LOC_Os07g32530*. The genomic positions of the target binding sites of the RC-miRNAs are shown on the top right corners of the screenshots of the public available epigenome browsers of *Arabidopsis* and rice [29], [30].

It is questionable that the RC-miRNAs with high accumulation levels only possess subtle regulatory roles in gene expression. A recent study by Qi’s group (2010) reminds us a potential role of miRNAs and miRNA-like sRNAs in transcriptional gene silencing through a DNA methylation pathway [28]. To address this possibility, the genomic locations of all the predicted target binding sites of the highly accumulated RC-miRNAs in *Arabidopsis* and rice were retrieved (see **Materials and Methods**, and Figure S4) for DNA methylation profiling queries by using the currently released epigenome browsers [29], [30]. Intriguingly, several coding regions generating transcripts targeted by specific RC-miRNAs were shown to be highly methylated. In many cases, the positions of the methylated genomic regions were highly correlated with the binding sites of the RC-miRNAs on the target transcripts (Figure 4, and Figure S5 and S6). These results indicate a potential role of certain RC-miRNAs in mediating DNA methylation in both *Arabidopsis* and rice. More interestingly, among the 19 observed methylated target genes in *Arabidopsis*, three genes were annotated to be involved in DNA or histone methylation, and two were implicated in root hair development (Table S6).

**Figure 5:**
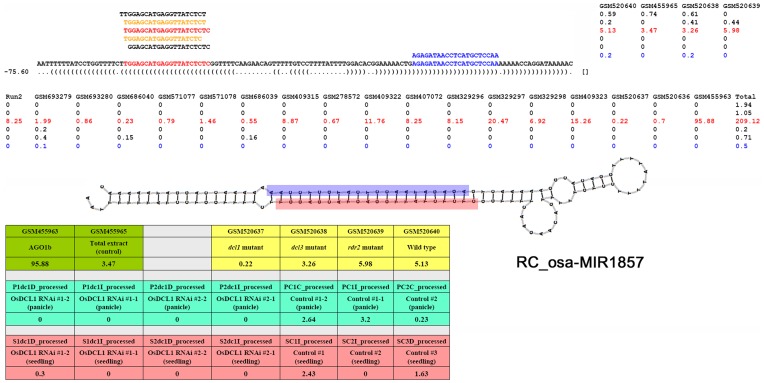
Dependence of RC-osa-miR1857 on DCL1, but not DCL3 and RDR2, and its enrichment in Argonaute 1. Secondary structure and the accumulation levels of the mature RC-miRNA (indicated by red color) and the RC-miRNA* (blue color) are shown on the top of the figure. The table at the bottom shows the accumulation levels of RC-osa-miR1857 in various biological samples, such as wild type seedling, *dcl1* mutant, *DCL1* RNAi (RNA interference) transgenic lines, *dcl3* mutant, *rdr2* mutant, and the sequencing data from Argonaute 1 (AGO1)-enriched small RNA population. Please note, the expression data (normalized in RPM, reads per million) was more comparable for the high-throughput sequencing data sets belonging to the same group (indicated by the same background color).

The recently reported lmiRNAs (long miRNAs) that mediating DNA methylation were confirmed to be DCL3-dependent, and were associated with AGO4 clade proteins, indicating a distinct regulatory pathway that they were involved in compared to the canonical miRNAs in plants [28]. In this regard, the DCL dependence and the AGO sorting patterns of the highly accumulated RC-miRNAs were investigated. Although the HTS data used for this interrogation were not sufficient to tell the biogenesis and the AGO sorting patterns for all the investigated RC-miRNAs (data not shown), some clear patterns were observed for certain RC-miRNAs (Table 1). In *Arabidopsis*, RC-ath-miR391 and RC-ath-miR2112 were enriched in AGO2 but not in AGO1, and the later one was also detected in AGO4. In rice, RC-osa-miR1857 and RC-osa-miR169p-1 were highly enriched in AGO1, whereas RC-osa-miR2118q was incorporated into AGO4 clade proteins, i.e. AGO4 and AGO16. RC-osa-miR1857, RC-osa-miR169p-1, and RC-osa-miR169q-2 showed dependence on DCL1 but not DCL3 for their biogenesis, and all of them were RDR2-independent. However, no evident dependence of the *Arabidopsis* RC-miRNAs on DCLs was observed which was like due to the small number (only five, see Figure S1) of the RC-miRNA precursors identified in this plant. Notably, in rice, clear dependence of the accumulation of RC-osa-miR1857 on DCL1, but not DCL3 and RDR2, and its enrichment in AGO1 protein complex were observed (see Figure 5 and Data S7), which strongly indicates that RC-osa-miR1857 share a common biogenesis pathway with the canonical miRNA genes [4]. Similar evidences were obtained for RC-osa-miR169p and RC-osa-miR169q (Data S7). Taken together, the RC-miRNAs likely form a novel miRNA-like population in addition to the canonical ones.

**Table 1:** High-throughput sequencing data-based biogenesis and Argonaute (AGO) sorting analysis of the highly accumulated reverse complementary microRNAs (RC-miRNAs) in *Arabidopsis* and rice.

Arabidopsis thaliana											
	GSE10036				GSE12037						
RC-miRNAs	AGO1 (GSM253622)	AGO2 (GSM253623)	AGO4 (GSM253624)	AGO5 (GSM253625)	AGO2 Input (GSM304282)	AGO2 Co-IP (GSM304283)					
RC-ath-miR2112	0	21.95	1.41	0	0	2.04					
RC-ath-miR391	0	7.32	0	0	0	2.73					
**Oryza sativa**											
	**GSE18250**				**GSE20748**				**GSE20748**		
**RC-miRNAs**	**AGO1a (GSM455962)**	**AGO1b (GSM455963)**	**AGO1c (GSM455964)**	**Total (GSM455965)**	**AGO4a (GSM520634)**	**AGO4b (GSM520635)**	**AGO16 (GSM520636)**	**Total_2 (GSM520640)**	**dcl1 (GSM520637)**	**dcl3 (GSM520638)**	**rdr2 (GSM520639)**
RC-osa-miR1857	0	95.88	0	3.47	0	0	0.7	5.13	0.22	3.26	5.98
RC-osa-miR2118q	0	0	0	0.74	1.47	2.8	61.02	0.39	0.22	0	0
RC-osa-miR169p-1 (osa-miR169b/c)	484.57	381.91	280.6	110.19	0	1.97	3.27	229.29	6.99	112.37	518.47
RC-osa-miR169q-2 (osa-miR169h/i/j/k/l/m)	7.39	0.81	0	15.14	0	0	0	209.54	0	57.61	48.92

**Note:** the accumulation data shown here were normalized read counts in RPM (reads per million).

## Discussion

### Sequences Antisense to the miRNA Precursors Possess Great Potential for Generating miRNA-like sRNAs

In this study, by using accumulation- and secondary structure-based screening, 59 RC-miRNA precursor candidates were identified in ten plant species (Figure S1 and Table S3). All the RC-miRNA precursors are capable of forming compelling hairpin-like structures. And, the mature RC-miRNAs along with the RC-miRNA*s were cloned by HTS, which were often supported by two or more distinct short sequences. Besides, the RC-miRNAs and the corresponding RC-miRNA*s could often form short duplexes with 2-nt 3′ overhangs at both ends, which were considered to be catalytic cleavage feature of the DCLs [3], [4]. Compared to the star species, the mature RC-miRNAs were usually accumulated much higher (Figure 1 and Figure S1). However, the accumulation levels of some RC-miRNAs generated from both arms of the same precursors were indistinguishable, and they were always accumulated at relatively low levels.

For the RC-miRNAs with total read counts higher than 20 RPM, we made an accumulation level-based comparison between these RC-miRNAs and the corresponding mature miRNAs. Although a portion of the miRNAs were cloned at much higher frequency than the RC-miRNAs identified on the antisense strands, many RC-miRNAs with accumulation levels comparable to the miRNAs or even much higher than the miRNAs were observed, such as those in *Glycine max*, *Medicago truncatula*, *Triticum aestivum*, *Vitis vinifera* and *Zea mays* (Table S7). The result indicates that the RC-miRNAs, at least some of them, accumulated at biologically relevant levels. On the other hand, for the RC-miRNAs with relatively low accumulation levels, we hypothesis that the RC-miRNA precursors may be newly born in specific plant species considering the factor that the newly evolved, less conserved miRNA genes were hardly detectable [31]. It was also observed that a large portion of the RC precursors were antisense to the less conserved plant miRNA genes. These evolutionary implications need to be investigated.

### RC-miRNAs, with a Role in Transcriptional Regulation through a DNA Methylation Pathway?

Although only a few cleavage targets of the highly accumulated RC-miRNAs were detected, several RC-miRNAs were shown to possess great potential to guide DNA methylation in both *Arabidopsis* (RC_ath-miR2112, RC_ath-miR391, RC_ath-miR781, RC_ath-miR782, and RC_ath-miR847) and rice (RC_osa-miR156, RC_osa-miR159, RC_osa-miR169, RC_osa-miR1846, RC_osa-miR2118, and RC_osa-miR535) (Figure 4 and Table S6). Except for the transposable element genes that were evenly methylated along their gene bodies, most protein-coding genes were specifically methylated at the target recognition sites of these RC-miRNAs, implying a sequence-specific mechanism. Interestingly, several target genes are suggested to be implicated in DNA or histone methylation in *Arabidopsis* according to the TAIR (Release 10) GO (Gene Ontology) annotations (Table S6). Moreover, previous study in rice uncovered the interplay between DNA methylation, histone methylation, and gene expression [32]. Thus, the scenario that RC-miRNAs repress genes with methylation-related functions through a DNA methylation pathway to form a feedback circuit maintaining a normal chromatin status *in planta* needs to be further investigated.

As mentioned above, a recent study discovered a new class of miRNAs called lmiRNAs. This miRNA species are DCL3-dependent, and are loaded into AGO4 clade protein-associated miRISCs to guide DNA methylation in rice [28]. In this study, the DCL-dependence and the AGO sorting patterns were analyzed for the highly accumulated RC-miRNAs in *Arabidopsis* and rice (Table 1). Three rice RC-miRNAs (RC-osa-miR1857, RC-osa-miR169p-1, and RC-osa-miR169q-2) showed clear evidences that their biogenesis was DCL1-dependent but DCL3- and RDR2-independent. Notably, RC-osa-miR2118q targeting three genes for site-specific DNA methylation (Figure S6 and Table S6) was indicated to be highly enriched in AGO4 and AGO16 (Table 1). Consistent with its DNA methylation role, the rice AGO4 clade proteins including AGO16 were confirmed to play an important role in transcriptional gene silencing in plants [28], [33]. Interestingly, in *Arabidopsis*, the only two RC-miRNAs showing clear AGO enrichment patterns were indicated to be associated with AGO2 (Table 1). Although some evidences pointed to the roles of AGO2 in antiviral defense [34]–[36], the mechanisms of AGO2-mediated gene silencing remain to be uncovered [33].

The sequence characteristics of all the RC-miRNAs and the highly accumulated ones identified in nine plant species were analyzed separately (Figure 2). Previous results show us that AGO1 preferentially recruits miRNAs with 5′ U, AGO2 and AGO4 harbor sRNAs started with 5′ A, and AGO5 predominantly binds sRNA with 5′ C in *Arabidopsis* [37]. Accordingly, the various 5′ nucleotide compositions are consistent with the diverse AGO sorting patterns of RC-miRNAs observed in this study (Table 1), and indicate the multiple manners for them to exert regulatory roles in gene expression control.

Taken together, whether the RC-miRNAs could be generated by a specific DCL-dependent pathway, and exert target-specific repressive regulation by guiding certain AGO-associated silencing complexes need experimental confirmation. The results obtained in this study could serve as the basis for further studies on this research topic.

## Materials and Methods

### Data Sets Used in this Study

The sRNA HTS data sets of Arabidopsis lyrata, Arabidopsis thaliana, Carica papaya, Citrus sinensis, Glycine max, Gossypium arboretum, Gossypium hirsutum, Hordeum vulgare, Medicago truncatula, Oryza sativa, Populus trichocarpa, Solanum lycopersicum, Sorghum bicolor, Triticum aestivum, Vitis vinifera and Zea mays were retrieved from GEO (Gene Expression Omnibus; http://www.ncbi.nlm.nih.gov/geo/) [38], CSPSR (Comparative Sequencing of Plant Small RNAs; http://smallrna.udel.edu/project_data.php), CSRDB (Cereal Small RNAs Database; http://sundarlab.ucdavis.edu/smrnas/) [39], and TomFuncDB (Tomato Functional Genomics Database; http://ted.bti.cornell.edu/cgi-bin/TFGD/sRNA/download.cgi) [40]–[42]. See Table S1 for the accession numbers. The degradome sequencing data sets of Arabidopsis thaliana, Oryza sativa and Glycine max were retrieved from GEO or NGSDBs (Next-Gen Sequence Databases; http://mpss.udel.edu/) [43]. See Table S2 for the accession numbers. The miRNAs and their precursor sequences of the 16 plant species were downloaded from miRBase (Release 17; http://www.mirbase.org/) [9]. The cDNAs, full-length genomic sequences, and the gene annotations of Arabidopsis, rice and soybean were retrieved from the FTP sites of The Arabidopsis Information Resource (TAIR, Release 7 and 10; ftp://ftp.arabidopsis.org/home/tair/Sequences/blast_datasets/) [18], the rice genome annotation project established by The Institute for Genome Research (currently named the J. Craig Venter institute) (TIGR rice, Release 5 and 6.1; ftp://ftp.plantbiology.msu.edu/pub/data/Eukaryotic_Projects/o_sativa/annotation_dbs/pseudomolecules/) [19], and GmGDB belonging to PlantGDB (version Glyma1) [20] (ftp://ftp.plantgdb.org/download/Genomes/GmGDB/), respectively.

### HTS Data-based Identification of RC-miRNA Candidates

First, the sequences reverse complementary to the miRNA precursors were generated for further analyses. In order to allow cross-library comparison, the normalized read count (in RPM, reads per million) of a short sequence from a specific library was calculated by dividing the raw count of this sequence by the total counts of the library, and then multiplied by 10^6^. Then, all the short sequences were mapped onto the RC sequences by BLAST algorithm [44], and all the perfectly matched ones were retained. Finally, manual identification of the RC-miRNA precursor candidates was carried out as follows: (1) there should be two or more isolated short-sequence clusters on the RC sequences, and the RC sequences with numerous continuously distributed (ladder-like) sequences without clear-cut boundaries were discarded. (2) according to the previously proposed criterion for miRNA locus identification [16], and considering that the sRNA HTS data of *Arabidopsis* and rice were much more abundant than the other plants’, three or more distinct short sequences with identical 5′ ends should appear in at least one isolated short-sequence clusters on each RC sequence for *Arabidopsis* and rice, and two or more short sequences with identical 5′ ends should appear in at least one isolated short sequence clusters for the other plant species. All these multi-sequences-supported sRNAs served as the mature RC-miRNA candidates for selection in the further analyses. The RC sequences met the above two filtering criteria were retained for further validation.

### Structure-based Validation of RC-miRNA Candidates

The retained RC sequences were subjected to secondary structure prediction by using RNAshapes in “Shape folding” mode with default parameters [17]. The simplest structure (with single stem-loop region in most cases) among all the predicted results for one RC sequence was selected for manual check. The RC sequences capable of forming stable hairpin-like structures were retained. The canonical miRNA/miRNA* short duplex possesses 2-nt 3′ overhangs [3], [4]. In this regard, the RC-miRNA candidates and the corresponding RC-miRNA*s were selected from the isolated short sequence clusters (as mention above, the RC-miRNA candidates should be supported by three or more sequences with identical 5′ ends in *Arabidopsis* and rice, and two or more sequence in the other plants analyzed; and the RC-miRNA* candidates were also supported by several sequences with identical 5′ ends in some cases), and were mapped onto the stem-loop-structured RC sequences and subjected to manual check. The hairpin-like RC sequences that could generate RC-miRNA/RC-miRNA* duplexes with 0- to 3-nt 3′ overhangs were considered to be the final RC-miRNA precursor candidates. The RC-miRNA candidates with normalized total read counts larger than 20 RPM in *Arabidopsis*, rice and soybean were selected for target prediction.

### Cleavage Target Prediction and Degradome Sequencing-based Verification

Target prediction was performed by using miRU algorithm [21], [22] with default parameters. The degradome sequencing data were utilized to validate the predicted RC-miRNA–target pairs. First, the read counts of all the degradome signatures from each library were normalized as described above. Then, two-step filtering was performed to extract the most likely RC-miRNA–target pairs. During the first step, the predicted RC-miRNA binding sites along with the 50-nt surrounding sequences at both ends were collected in order to reduce the BLAST time. For the BLAST, all the collected degradome data sets (eleven of *Arabidopsis*, four of rice and one of soybean; see Table S2) were utilized at the same time to do a comprehensive search. It was based on the scenario that a RC-miRNA–target pair was considered to be the candidate once the cleavage signal(s) existed in any data set(s). Two types of predicted targets were retained for further filtering: (1) there must be perfectly matched degradome signatures with their 5′ ends resided within 8–14 nt region away from the 5′ ends of the target binding sites; or (2) the target transcripts should possess degradome signatures at least partially located within the target binding sites, and their normalized counts should be significantly higher than the surrounding signals. These transcripts were subjected to a second BLAST, and the degradome signals along each transcript were obtained to provide a global view of the signal noise when compared to the signal intensity within a specific target binding site. Referring to our previous study [25], both the global and the local t-plots were drawn. Exhaustive manual filtering was performed, and only the transcripts with cleavage signals easy to be recognized were extracted as the potential RC-miRNA–target pairs.

### Analysis of RC-miRNA-guided Target DNA Methylation

According to the target prediction results produced by miRU, the TAIR 10- and TIGR rice 6.1-based genomic positions of the predicted target binding sites of the RC-miRNA candidates (with normalized total read counts larger than 20 RPM) were converted into TAIR 7- and TIGR rice 5-based genomic positions, respectively. Then, the binding sites were used for DNA methylation profile queries through (http://epigenomics.mcdb.ucla.edu/DNAmeth/) [30] and (http://159.226.118.31∶9311/cgi-bin/hgGateway) [29], which were built based on the annotated gene information of TAIR 7 and TIGR rice 5 respectively. Please see Figure S4 for the clear workflow.

## Supporting Information

Figure S1 **Structure- and accumulation level-based identification of plant RC-miRNAs.**
(PDF)

Figure S2 **Degradome data-based validation of RC-miRNA-regulated target transcripts in** ***Arabidopsis*** **.**
(PDF)

Figure S3 **Degradome data-based validation of RC-miRNA-regulated target transcripts in rice.**
(PDF)

Figure S4 **Schematic workflow of converting the genomic positions of target binding sites from TAIR 10 and TIGR rice 6.1 to TAIR 7 and TIGR rice 5 for DNA methylation profile queries in** ***Arabidopsis*** **and rice.**
(PDF)

Figure S5 **RC-miRNA-guided DNA methylation surrounding their target recognition sites in** ***Arabidopsis*** **.**
(PDF)

Figure S6 **RC-miRNA-guided DNA methylation surrounding their target recognition sites in rice.**
(PDF)

Table S1 **Plant small RNA high-throughput sequencing data sets used in this study.**
(PDF)

Table S2 **Plant degradome sequencing data sets used in this study.**
(PDF)

Table S3 Sequence information of identified mature RC-miRNAs, RC-miRNA*s, and RC-miRNA precursors.
(XLS)

Table S4 **List of mature RC-miRNAs used for cleavage target identification and DNA methylation analysis in** ***Arabidopsis*** **and rice, and the mature RC-miRNAs with normalized total read counts more than 20 RPM (reads per million) in other plant species (the mature RC-miRNAs of soybean with accumulation levels higher than 20 RPM were also used for degradome sequencing data-based target identification).**
(XLS)

Table S5 **List of identified RC-miRNA–cleavage target regulatory relationships in** ***Arabidopsis*** **and rice.**
(XLS)

Table S6 **RC-miRNA-guided DNA methylation and their target genes in** ***Arabidopsis*** **and rice.**
(XLS)

Table S7 **Comparison of the accumulation levels between RC-microRNAs and the corresponding microRNAs in the sixteen plant species (only the RC-miRNAs with accumulation levels higher than 20 RPM were included).**
(PDF)

Data S1 **Randomly selected 1000 sequences antisense to the protein-coding genes in** ***Arabidopsis*** **.**
(TXT)

Data S2 **Randomly selected 1000 sequences antisense to the protein-coding genes in rice.**
(TXT)

Data S3 **Retained random sequences after secondary structure prediction in** ***Arabidopsis*** **.**
(TXT)

Data S4 **Retained random sequences after secondary structure prediction in rice.**
(TXT)

Data S5 **Random sequences possessing perfectly-matched small RNAs in** ***Arabidopsis*** **.**
(TXT)

Data S6 **Random sequences possessing perfectly-matched small RNAs in rice.**
(TXT)

Data S7 Dependence of the accumulation levels of certain RC-miRNA(*)s on DCL1, but not DCL3 and RDR2, and their association with AGO1 protein.
(XLS)
