# Supplementary material for: Genome-Wide Identification of Reverse Complementary microRNA Genes in Plants
Source: PLoS One. 2012 Oct 23;7(10):e46991. doi: 10.1371/journal.pone.0046991 (PMC3479107; doi:10.1371/journal.pone.0046991)

	GSM518429	Total
CACCATGTTTAGACTCTGAA	0.25	0.25
ATTCAGAGTCTAAACATGGTG	0.74	0.74
TTCAGAGTCTAAACATGGTGTT	0.74	0.74
TTCAGAGTCTAAACATGGTGT	6.19	6.19
TCAGAGTCTAAACATGGTGTT	0.5	0.5

```
       AGACTGAAATTTTACATTTTTATACTAAACACCATGTTTAGACTCTGAATTTTTTTCCATATAATAAATAAGTCCTTGAGACCGATTTAGGAAAAAAGTCTTTGCCTTTTAAAATAAAATTATATGGAAAAAATTCAGAGTCTAAACATGGTGTTTAGTATAAAAATGTAAAATTTCAGTCT
-91.76 (((((((((((((((((((((((((((((((((((((((((((((((((.(((((((((((((((...((((.....(((((...............))))).....)))).......))))))))))))))))))))))))))))))))))))))))))))))))))))))))))))))))  []
```

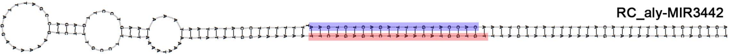

RC_aly-MIR3442

```
                                                                                                                                          GSM518430 GSM518431 Total
                                        TAATCTCACAAGGCACCGGAA                                                                             0.21      0         0.21
                                                                                            TTTTTCGGTGCATGGCGGGATTAG                      0.21      0         0.21
                                                                                             TTTTCGGTGCATGGCGGGATTAG                      0.82      0.47      1.29
                                                                                             TTTTCGGTGCATGGCGGGATT                        0.62      0         0.62
                                                                                                TCGGTGCATGGCGGGATTAGA                     45.93     33.34     79.27
                                                                                                TCGGTGCATGGCGGGATTAGAA                    0         0.24      0.24
                                                                                                TCGGTGCATGGCGGGATTAG                      0         0.24      0.24
                                                                                                TCGGTGCATGGCGGGATTAGAATT                  0.62      0.24      0.86
                                                                                                TCGGTGCATGGCGGGAT                         0.21      0         0.21
                                                                                                  GGTGCATGGCGGGATTAGAATT                  0.21      0         0.21
                                                                                                  GGTGCATGGCGGGATTAGA                     0.41      0         0.41
        AACTCCTTAAGCAAATTCACAAGATAAAACTCTAATCTCACAAGGCACCGGAAAACCACAGACTGTATTATTTCAGTCTAGAATTTTTTCGGTGCATGGCGGGATTAGAATTTAGTTTTGCTGATTAGCCAAATT
-44.00  ..........((.((((..((((((.(((.(((((((((.((..((((((((((((....((((((........)))))).....))))))))))).))..))))))))).))).))))))..)))).)).......   []
```

RC_aly-MIR3443

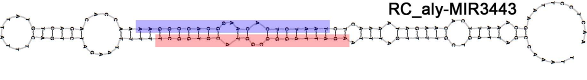

Sequence	GSM518429	GSM518430	GSM518431	GSM518392	Total
TTAAACCTCCTTGTTGCCATC	0.5	0	0	0	0.5
TCCTTGTTGCCATCGGTCTTG	0.74	0	0	0	0.74
TCCTTGTTGCCATCGGTCTTGGC	0.25	0	0	0	0.25
AGCGAGCGCCAAGAACGATGG	0	0.41	0.47	24.27	25.15
CCAAGAACGATGGCAACAC	0	0	0.24	0	0.24
CCAAGAACGATGGCAACACAA	0	0	0.24	0	0.24
CAAGAACGATGGCAACACAAAGGTCT	0	0	0.47	0	0.47
CAAGAACGATGGCAACACAAAG	0	0.41	0	0	0.41
AGAACGATGGCAACACAAAGG	0	0.21	0	0	0.21
TCTAACTGCCTTTATGACCTGAT	0	0.21	0	0	0.21
CTAACTGCCTTTATGACCTGA	0.25	0	0	0	0.25

```
      AAAAATCAGCTCAGAAGGCGGTTAAACCTCCTTGTTGCCATCGGTCTTGGCGCTTTTGGTGAAGCGAGCGCCAAGAACGATGGCAACACAAAGGTCTAACTGCCTTTATGACCTGATT
-67.90 ...((((((.(((((((((((((((.((((...(((((((((((.((((((((((..........)))))))))).)))))))))))...)))).)))))))))))).))).))))))   []
```

RC_aly-MIR4231

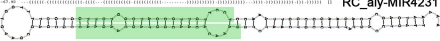

Sequence	GSM442932	GSM366865	GSM343005	GSM442934	GSM642338	GSM456945	GSM442935	GSM253624	GSM118372	GSM120717	GSM253623	GSM304283	GSM343004	GSM343000	Total
ATGATGCGCAAATGCGGATAT	0.55	0.58	0	0.26	0	4.46	1.09	1.41	4.86	0	21.95	2.04	0	0	37.2
ATGATGCGCAAATGCGGATA	0	0	0.72	0	0	0	0	0	0	0	0	0	0	0	0.72
ATGATGCGCAAATGCGGA	0	0	0	0	0.15	0	0	0	0	0	0	0	0	0	0.15
ATGATGCGCAAATGCGG	0	0	0	0	0	0	0	0	0	2.54	0	0	0	0	2.54
TGATGCGCAAATGCGGATAT	0	0	0	0	0	0.6	0	0	0	0	0	0	0	0	0.6
TGATGCGCAAATGCGGATA	0	0	0	0	0	0.15	0	0	0	0	0	0	0.3	0	0.45
ATTGATATCCGCATTTGCGCA	0	0	0	0	0	0	0	0	0	0	0	0	0	1.22	1.22
ATCCGCATTTGCGCATC	0	0	0	0	0	0	0	0	0	2.54	0	0	0	0	2.54

```
      GTCAGAGATGATGCGCAAATGCGGATATAAAGGTATATCATGACCCCAATGTTTCTGATAAACCCTTTTCACATTTTGTTGTTTTCGCTGAGCACCTCTATTGGTTTGTTGTCCTGATTTACATTGATATCCGCATTTGCGCATCATTTCATAC
-56.60 ....((((((((((((((((((((((....(((.((((.(((............(((((((...............(((((.....)))))........))))))))))).)))).)))....)))))))))))))))))))))))....  []
```

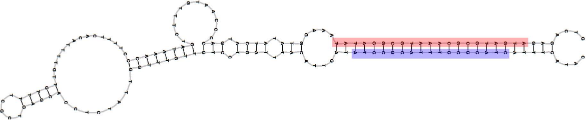

RC_ath-MIR2112

	GSM642338	GSM642337	GSM442935	GSM642336	GSM518432	GSM642335	GSM343005	GSM366866	GSM343001	GSM253623	GSM304283	GSM342999	GSM442934	GSM257236	GSM253622	GSM253624	GSM304282	GSM120717	GSM456945	GSM118373	Total
AGGGTGATTATTGAACAACTGA	0.1	0	0	0	0	0	0	0	0	0	0	0	0	0	0	0	0	0	0	0	0.1
ATAAGGATTGCTACGTAGGAG	2.65	0	0	0	0	0	0	0	0	0	0	0	0	0	0	0	0	0	0	0	2.65
ATAAGGATTGCTACGTAGG	0.05	0	0	0	0	0	0	0	0	0	0	0	0	0	0	0	0	0	0	0	0.05
ATAAGGATTGCTACGTAGGAGAGA	0.15	0.26	0	0	0	0	0	0	0	0	0	0	0	0	0	0	0	0	0	0	0.41
TAAGGATTGCTACGTAGGAGA	4.45	0.13	0	0	0	0	0	0	0	0	0	0	0	0	0	0	0	0	0	0	4.58
TAAGGATTGCTACGTAGGAG	0.65	0	0.27	0	0	0	0	0	0	0	0	0	0	0	0	0	0	0	0	0	0.92
TAAGGATTGCTACGTAGGAGAG	0.05	0	0	0	0	0	0	0	0	0	0	0	0	0	0	0	0	0	0	0	0.05
AAGGATTGCTACGTAGGAGA	0.7	0.13	0	0	0	0	0	0	0	0	0	0	0	0	0	0	0	0	0	0	0.83
AAGGATTGCTACGTAGGAGAG	295.7	82.06	2.17	3.05	0.72	0.05	1.07	0.43	0.81	7.32	2.73	0.76	2.65	21.16	0	0	0	0	0	0	420.68
AAGGATTGCTACGTAGGAGAGA	0.15	0	0	0	0	0	0	0	0	0	0	0	0	0	0	0	0	0	0	0	0.15
AAGGATTGCTACGTAGGA	0.3	0	0	0	0	0	0	0	0	0	0	0	0	0	0	0	0	0	0	0	0.3
AAGGATTGCTACGTAGGAG	0.05	0	0	0	0	0	0	0	0	0	0	0	0	0	0	0	0	0	0	0	0.05
AGGATTGCTACGTAGGAGAGA	0.25	0	0	0	0	0	0	0	0	0	0	0	0	0	0	0	0	0	0	0	0.25
AGGATTGCTACGTAGGAGAG	0.2	0	0.27	0	0	0	0	0	0	0	0	0	0.26	0	0	0	0	0	0	0	0.73
GGATTGCTACGTAGGAGAG	0.05	0	0	0	0	0	0	0	0	0	0	0	0	0	0	0	0	0	0	0	0.05
GATTGCTACGTAGGAGAG	0.3	0	0	0	0	0	0	0	0	0	0	0	0	0	0	0	0	0	0	0	0.3
ATTGCTACGTAGGAGAGAT	0.05	0	0	0	0	0	0	0	0	0	0	0	0	0	0	0	0	0	0	0	0.05
ATTGCTACGTAGGAGAGATAC	20.13	2.1	0	0	0	0	0	0	0	43.9	0	0	0	0	1.19	4.22	0	0	0	0	71.54
ATTGCTACGTAGGAGAGA	0.8	0	0	0	0	0	0	0	0	0	0	0	0	0	0	0	0	0	0	0	0.8
ATTGCTACGTAGGAGAGATA	11.89	1.58	0.27	0	0	0	0	0	0	0	0	0	0	0	0	1.41	0	0	0	0	15.15
TACGTAGGAGAGATACCGTCACCA	0	0	0	0	0	0	0	0	0	0	0	0	0	0	0	0	1.46	0	0	0	1.46
ATACCGTCACCACTAGTTAACT	0.05	0.13	0	0	0	0	0	0	0	0	0	0	0	0	0	0	0	0	0	0	0.18
ATGGCGCTATCTCTCCTGCGAAGC	0	0	0	0	0	0	0	0	0	0	0	0	0	0	0	1.41	0	0	0	0	1.41
TATCTCTCCTGCGAAGC	0	0	0	0	0	0	0	0	0	0	0	0	0	0	0	0	0	16.52	0	0	16.52
ATCTCTCCTGCGAAGCAAATCT	0	0	0	0	0	0	0	0	0	0	0	0	0	0	0	1.41	0	0	0	0	1.41
TCTCTCCTGCGAAGCAAATCT	0	0	0	0	0	0	0	0	0	0	0	0	0	0	0	0	0	0	0.15	5.35	5.5
TCTCCTGCGAAGCAAATCT	0	0	0	0	0	0	0	0	0	7.32	0	0	0	0	0	0	0	0	0	0	7.32
CTCCTGCGAAGCAAATCTTTA	0.25	0	0	0	0	0	0	0	0	0	0	0	0	0	0	0	0	0	0	0	0.25

TAGAGGGTGATTATTGAACAACTGAAATACCTTGATCTCTCCGTATTAAGATGCATATATAAGGATTGCTACGTAGGAGAGATACCGTCACCACTAGTTAACTTCTTAGAAGAGGTGATGGCGCTATCTCTCCTGCGAAGCAAATCTTTATTTGCAGTTTTAAAACCATAGAAGAAGCAAGACCACTTGTATGTGTAATCACTCTA

-74.80 ...((((((((((...((((.((......((((.(((((..((.((((((((((...((((((((((((.(((((((((((((((((((((.((..((((....)))).)).)))))))))...)))))))))))))).))))).))))))).)))).))))))).))...))).))..)))))..)).))))....))))))))))). []

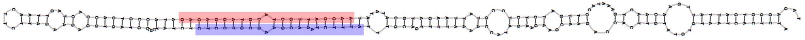

RC_ath-MIR391

	GSM366868	GSM366867	GSM366869	GSM442932	GSM366870	GSM366866	GSM343004	GSM343002	GSM343005	GSM366865	GSM442335	GSM518432	GSM442336
TTAGAGTTTTCTGGATACTTA	5.76	7.39	15.18	1.38	10.41	8.2	4.53	7.37	8.24	6.97	4.29	2.41	7.33
TTAGAGTTTTCTGGATACTTAA	0	0	0	0	0	0	0	0.57	0	0	0	0	0
TTAGAGTTTTCTGGATACTT	0	0	0	0	0	0	0	0	1.79	0	0	0	0
TTAGAGTTTTCTGGATAC	0	0	0	0	0	0	0	0	0	0	0	0	0
TTAGAGTTTTCTGGATACT	0	0	0	0	0	0	0	0	0	0	0	0	0
TTAGAGTTTTCTGGATA	0	0	0	0	0	0	0	0	0	0	0	0	0
AGAGTTTTCTGGATACTTA	0	0	0	0.28	0	0	0	0	0	0	0	0	0
AATAAGTATCCAGAAAACTCT	0	0	0	0	0	0	0	0	0	0	0	0	0
AGTATCCAGAAAACTCTAATTT	0	0	0	0	0	0	0	0	0	0	0	0	0

	GSM442338	GSM442337	GSM442935	GSM442933	GSM442934	GSM149080	GSM121457	GSM253622	GSM253625	GSM253623	GSM121456	GSM118375	GSM118374
TTAGAGTTTTCTGGATACTTA	6.79	10.64	2.99	1.44	2.12	19.73	93.79	4.75	3.24	21.95	59.5	13.08	10.58
TTAGAGTTTTCTGGATACTTAA	0	0	0	0	0	0	0	0	0	0	0	0	0
TTAGAGTTTTCTGGATACTT	0	0	0	0	0	0	0	0	0	0	0	0	0
TTAGAGTTTTCTGGATAC	0	0	0	0.29	0.26	0	0	0	0	0	0	0	0
TTAGAGTTTTCTGGATACT	0	0	0.27	0	0	0	0	0	0	0	0	0	0
TTAGAGTTTTCTGGATA	0	0	0	0	0	0	0	0	0	0	0	0	0
AGAGTTTTCTGGATACTTA	0	0	0	0	0	0	0	0	0	0	0	0	0
AATAAGTATCCAGAAAACTCT	0	0	0	0	0.26	0	0	0	0	0	0	0	0
AGTATCCAGAAAACTCTAATTT	0	0.13	0	0	0	0	0	0	0	0	0	0	0

	GSM120717	GSM121455	GSM121453	GSM257235	GSM342999	GSM343001	GSM343000	GSM304283	GSM304284	GSM304282	GSM257237	Total
TTAGAGTTTTCTGGATACTTA	0	66.39	140.69	13.31	7.96	11.69	8.56	1.36	7.34	1.46	18.72	637.53
TTAGAGTTTTCTGGATACTTAA	0	0	0	0	0	0	0	0	0	0	0	0.57
TTAGAGTTTTCTGGATACTT	0	0	0	13.31	1.14	0	0	0	0	0	0	16.24
TTAGAGTTTTCTGGATAC	0	0	0	0	0	0	0	0	0	0	0	0.55
TTAGAGTTTTCTGGATACT	0	0	0	0	0.38	0	0	0	0	0	0	0.65
TTAGAGTTTTCTGGATA	97.87	0	0	0	0	0	0	0	0	0	0	97.87
AGAGTTTTCTGGATACTTA	0	0	0	0	0	0	0	0	0	0	0	0.28
AATAAGTATCCAGAAAACTCT	0	0	0	0	0	0	0	0	0	0	0	0.26
AGTATCCAGAAAACTCTAATTT	0	0	0	0	0	0	0	0	0	0	0	0.13

TCAAATTTAGAGTTTTCTGGATACTTAAAAGTTAATATCGAAGAAGCCCTCTCTTTGTTAGTAACTAATAAGTATCCAGAAAACTCTAATTTGA

-43.20 ((((.((((((((((((((((((((..(((((....((((((.......))))))....))))).))))))))))))))))))))))))) []

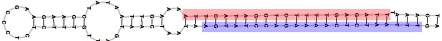

RC_ath-MIR781

	GSM343005	GSM342999	GSM366868	GSM343000	GSM304284	GSM304285	GSM304283	GSM343001	GSM366865	GSM366867	GSM343002	GSM518432	GSM343004	GSM304282	GSM149079	GSM149080	GSM154336	GSM642338	GSM257236	GSM120717	GSM121453	GSM253625	GSM642337	GSM442934	GSM118374	Total
CTCTTTCTGCAAACGCCTTGGA	0.36	0	0	0	0	0	0	0	0	0	0	0	0	0	0	0	0	0	0	0	0	0	0	0	0	0.36
TCTTTCTGCAAACGCCTTGGA	32.61	13.65	1.44	18.74	14.67	6.08	29.98	24.16	0.58	1.71	30.05	1.21	18.73	26.19	19.5	6.58	12.72	0.1	0	0	0	0	0	0	0	258.7
TCTTTCTGCAAACGCCTTGG	0	0	0	0	0	0	0	0	0	0	0	0	0	1.46	0	0	0	0	0	0	0	0	0	0	0	1.46
CTTTCTGCAAACGCCTTGGAT	0	0	0	0	0	0	0	0	0	0	0	0	0	0	0	0	0	0	21.16	0	0	0	0	0	0	21.16
CTTTCTGCAAACGCCTTGGATT	0	0	0	0	0	0	0	0	0	0	0.57	0	0	0	0	0	0	0	0	0	0	0	0	0	0	0.57
CTTTCTGCAAACGCCTTGGA	0.36	0.76	0	0	0	0	0	0	0	0	0	0	0	0	0	0	0	0	0	0	0	0	0	0	0	1.12
TTTCTGCAAACGCCTTGGA	0.36	0.76	1.44	0	0	0	0	0	0	0	0.57	0	0.3	0	0	0	0	0	0	0	0	0	0	0	0	3.43
TTTCTGCAAACGCCTTGGATT	1.43	0	0	0	0	0	0	0.4	0	0	0	0	0	0	0	0	0	0	0	0	0	0	0	0	0	1.83
TTTCTGCAAACGCCTTG	0	0	0	0	0	0	0	0	0	0	0	0	0	0	0	0	0	0	0	1.27	0	0	0	0	0	1.27
TTCTGCAAACGCCTTGGA	0.36	0	0	0	0	0	0	0	0	0	0	0	0	0	0	0	0	0	0	0	0	0	0	0	0	0.36
TCTGCAAACGCCTTGGATTTTCTT	0	0	0	0	0	0	0	0	0	0	0	0	0	1.46	0	0	0	0	0	0	0	0	0	0	0	1.46
TCTGCAAACGCCTTGGA	0	0	0	0	0	0	0	0	0	0	0	0	0	0	0	0	0	0	0	11.44	0	0	0	0	0	11.44
TCTGCAAACGCCTTGGATTTT	0	0.38	0	0	0	0	0	0	0	0	0	0	0	0	0	0	0	0	0	0	140.69	0	0	0	0	141.07
TCTGCAAACGCCTTGGATTT	0	0	0	0	0	0	0	0	0	0	0	0	0	0	0	0	12.72	0	0	0	0	0	0	0	0	12.72
GCAAACGCCTTGGATTTTCTT	0	0	0	0	0	0	0	0	0	0	0	0.24	0	0	0	0	0	0	0	0	0	0	0	0	0	0.24
CAAACGCCTTGGATTTTCTTT	0	0	0	0	0	0	0	0	0	0	0	0	0	0	0	0	0	0	0	0	0	3.24	0	0	0	3.24
GAACATCCAAGGTGTTTGTAGAAA	0	0.38	0	0	0	0	0	0	0	0	0	0	0	1.46	0	0	0	0	0	0	0	0	0	0	0	1.84
AACATCCAAGGTGTTTGTAGA	0	0	0	0	0	0	0	0	0	0	0	0	0	0	0	0	0	0.25	0	0	0	0	0	0	0	0.25
AACATCCAAGGTGTTTGTAGAAAA	0	0	0	0	0	0	0	0	0	0	0	0	0	0	0	0	0	0.05	0	0	0	0	0	0	0	0.05
ACATCCAAGGTGTTTGTAGAA	0	0	0	0	0	0	0	0	0	0	0	0	0	0	0	0	0	0.15	0	0	0	0	0	0	0	0.15
ACATCCAAGGTGTTTGTAGAAA	0	0	0	0	0	0	0	0	0	0	0.28	0	0	0	0	0	0	0.4	0	0	0	0	0	0	0	0.68
CATCCAAGGTGTTTGTAGAAA	0	0.76	0	0	3.67	0	0	0	0	0	1.13	0	0	0	0	0	25.45	1	0	0	0	0	0.92	0.53	5.29	38.75
ATCCAAGGTGTTTGTAGAA	0	0	0	0	0	0	0	0	0	0	0	0	0	0	0	0	0	0.05	0	0	0	0	0	0	0	0.05
ATCCAAGGTGTTTGTAGAAA	0	0	0	0	0	0	0	0	0	0	0	0	0	0	0	0	0	0.1	0	0	0	0	0	0	0	0.1
CAAGGTGTTTGTAGAAAAAGG	0.36	0	0	0.81	0	0	0	0	0	0	0.57	0	1.51	0	0	0	0	0	0	0	0	0	0	0	0	3.25
CAAGGTGTTTGTAGAAAAAG	0	0	0	0	0	0	0	0	0	0	0	0	0.3	0	0	0	0	0	0	0	0	0	0	0	0	0.3

AACCTCTTTCTGCAAACGCCTTGGATTTTCTTTTGGCTTCATGTTGTTCATTAGAATAATAGCCTTTTCATCTTATTTAAAGAACATCCAAGGTGTTTGTAGAAAAAGGTT

AACCTCTTTCTGCAAACGCCTTGGATTTTCTTTTGGCTTCATGTTGTTCATTAGAATAATAGCCTTTTCATCTTATTTAAAGAACATCCAAGGTGTTTGTAGAAAAAGGTT

-49.66 ((((.((((((((((((((((((.((((((.((((....(((((((....))))))))))..............)))))).)))))))))))))))))).))))) []

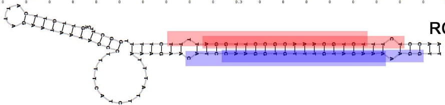

RC_ath-MIR782

Sequence	GSM642338	GSM642337	GSM442934	GSM442935	GSM118372	GSM253624	GSM343001	GSM366867	GSM118375	GSM118373	GSM518432	GSM366865	GSM366866	GSM120717	GSM342999	GSM343005	GSM642336	GSM642335	Total
AGAAGAGGAGTGAAAGGCCGAG	0.25	0	0.79	0.27	0	0	0	0	0	0	0	0	0	0	0	0	0	0	1.31
AGAAGAGGAGTGAAAGGCC	0.4	0.13	0	0	0	0	0	0	0	0	0	0	0	0	0	0	0	0	0.53
AGAAGAGGAGTGAAAGGC	0.15	0	0	0	0	0	0	0	0	0	0	0	0	0	0	0	0	0	0.15
AGAAGAGGAGTGAAAGGCCGA	2.95	1.18	7.67	5.7	4.86	1.41	0	0	0	0	0	0	0	0	0	0	0	0	23.77
AGAAGAGGAGTGAAAGGCCGAGA	0	0	0.53	0	0	0	0	0	0	0	0	0	0	0	0	0	0	0	0.53
AGAGGAGTGAAAGGCCGAGAT	0.95	0.13	0	0	0	0	0	0	0	0	0	0	0	0	0	0	0	0	1.08
AGTGAAAGGCCGAGATACAGATCG	0	0	0	0.27	0	0	0	0	0	0	0	0	0	0	0	0	0	0	0.27
GGCCGAGATACAGATCGAAACATA	0	0	0	0	0	1.41	0	0	0	0	0	0	0	0	0	0	0	0	1.41
GGCCGAGATACAGATCGAAACA	0	0	0	0.27	0	1.41	0	0	0	0	0	0	0	0	0	0	0	0	1.68
TGTTTCAGTTTAGATTTCGGC	0	0	0	0	0	0	0.4	0	0	0	0	0	0	0	0	0	0	0	0.4
TTTCAGTTTAGATTTCGGCTTC	0.05	0	1.06	0.27	0	0	0	1.71	3.27	10.7	4.34	2.32	4.31	0	0	0	0	0	28.03
TTTCAGTTTAGATTTCGGCTT	0	0	0	0	0	0	0	0	0	0	0.72	0	0	0	0	0	0	0	0.72
TTCAGTTTAGATTTCGGCTTC	0	0	0.26	0.27	0	0	0	0.57	0	0	0	0.58	0	0	0.38	0	0	0	2.06
TTCAGTTTAGATTTCGG	0	0	0	0	0	0	0	0	0	0	0	0	0	3.81	0	0	0	0	3.81
TTCAGTTTAGATTTCGGCTTCC	0	0	0.26	0	0	0	0	0	0	0	0	0	0	0	0	0	0	0	0.26
ATTTCGGCTTCCCATTCCTCT	0.05	0	0	0	0	0	0	0	0	0	0	0	0	0	0	0	0	0	0.05
TTTCGGCTTCCCATTCCTCTT	0.45	1.18	0	0	0	0	0	0.57	0	0	0	0	0.43	0	0	0.36	0.84	0.05	3.88
TTCGGCTTCCCATTCCTCTTC	0	0	0	0	0	0	0	0	0	0	0.24	0	0	0	0	0	1.38	3.88	5.5
GGCTTCCCATTCCTCTTCAT	0	0	0.26	0	0	0	0	0	0	0	0	0	0	0	0	0	0	0	0.26

```
ATATCTAACAGATTTTTCACTCCATCTAATGTGTCGAATAACAAATTCTTACATCAAGAAGAAGAGGAGTGAAAGGCCGAGATACAGATCGAAACATAGATATCACTTACCAATTGATAATTGGGTGACGCCTCTGTTTCAGTTTAGATTTCGGCTTCCCATTCCTCTTCATCAAGATGTAAGAATCTTATTCTCTTCTTAACTTAAGATACTTTGAATACAAGAAAATG
ATATCTAACAGATTTTTCACTCCATCTAATGTGTCGAATAACAAATTCTTACATCAAGAAGAAGAGGAGTGAAAGGCCGAGATACAGATCGAAACATAGATATCACTTACCAATTGATAATTGGGTGACGCCTCTGTTTCAGTTTAGATTTCGGCTTCCCATTCCTCTTCATCAAGATGTAAGAATCTTATTCTCTTCTTAACTTAAGATACTTTGAATACAAGAAAATG
-71.63 .............(((((......((....((((((((((...(((((((((((..((.(((((((((((..((((((((((..((((.((((((.((...((((...(((((.....)))))))))...)).))))))..))))..))))))))))..)))))))))))).))..))))))))))).))))))..............)))))...)).......)))))..   []
```

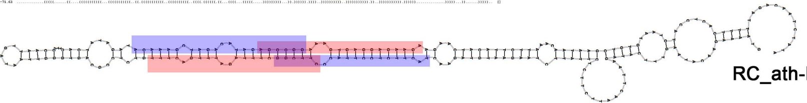

RC_ath-MIR847

	CSI2	Flower_trimmed	CSI1	Leaves_trimmed	Total
GCAGCATCATCAAGATTCTC	0.36		0		0.36
GCAGCATCATCAAGATTC	0		0.31		0.31
TGAATCTTGAGGACGCTGCTAC	0.36		0		0.36
GAATCTTGAGGACGCTGCTA	0.36		0		0.36
AGCGAGCAACGACTGTTTTAA	0		0.31		0.31

TTGTTGGTAGCTAGTCATTAATCGCCGTTGCAGCATCATCAAGATTCTCACTGACAAAAACCAAGGCAAGCAAAAATGAAGAAAAATTGCTGCTTTTGCCTTTAGACTGGATGTGAATCTTGAGGACGCTGCTACAGCGAGCAACGACTGTTTTAAGGTGAC

-50.40 ...((((.(((.((((.((..((((.((.(((((.((.(((((((((.((....((......((((((((((..(((........)))..))))..))))))).....))..)).))))))))).)).))))).)).))))..)).)))))))))))...... []

RC_csi-MIR172a

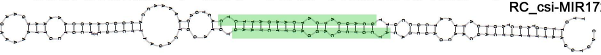

CSI2	Flower_trimmed	CSI3	Fruit_trimmed	Total
0.36		0		0.36
0.72		0		0.72
0		0.41		0.41

```
                              CCCACAGCTTTATTGAAC
                              CCCACAGCTTTATTGAACC
                                                                                               TTCAAGAAAGCTGTGGAAAAA
       TTGAAATTTTATTGAACCTGTTTGTATCTTCCCACAGCTTTATTGAACCGCAACAAATGCGATGCCAAGTCTGTGGCCTCATGCAAAGATGATGCAGTTCAAGAAAGCTGTGGAAAAATACAAAGAGGATCATA
-51.40 ............(((.(((.((((((((.((.((((((((((.((((((.(((.((..(((((.((((......)))).))..)))....)).))).)))))).)))))))))).)).))))))).))).)))..  []
```

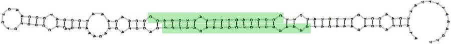

RC_csi-MIR396b

Sequence	GSM643816	GSM643815	Total
TGTCAAACAACAGGACTCAAGCA	0.12	0	0.12
TCAAACAACAGGACTCAAGCAT	0.12	0.12	0.24
CAAACAACAGGACTCAAGCAT	0.12	0.12	0.24
CAAACAACAGGACTCAAGCATT	0.12	0.49	0.61
CAAACAACAGGACTCAAGCATTTC	0.12	0.24	0.36
AAACAACAGGACTCAAGCATT	0.12	0.49	0.61
AAACAACAGGACTCAAGC	0	0.12	0.12
AAACAACAGGACTCAAGCATTTCG	0.24	0.49	0.73
AACAACAGGACTCAAGCATTTCGC	2.52	3.05	5.57
AACAACAGGACTCAAGCATTTCG	0	0.12	0.12
ACAACAGGACTCAAGCATTTCGC	0.12	0.37	0.49
ACAACAGGACTCAAGCATTTCGCA	0.12	0.49	0.61
ACAACAGGACTCAAGCATTTC	0.48	0.24	0.72
CAACAGGACTCAAGCATT	1.44	1.83	3.27
CAACAGGACTCAAGCATTTCGC	14.49	18.94	33.43
CAACAGGACTCAAGCATTT	0.12	0.12	0.24
CAACAGGACTCAAGCAT	0.12	0.12	0.24
CAACAGGACTCAAGCATTTCGCA	0.12	0	0.12
CAACAGGACTCAAGC	0.36	0	0.36
CAACAGGACTCAAGCATTTCGCAA	0.12	0	0.12
CAACAGGACTCAAGCATTTCG	11.14	18.33	29.47
AACAGGACTCAAGCATTTCG	0.24	0	0.24
AACAGGACTCAAGCATTTCGC	7.19	9.41	16.6
AACAGGACTCAAGCATTTCGCAAG	0.12	0.12	0.24
ACAGGACTCAAGCATT	0.12	0	0.12
ACAGGACTCAAGCATTTCGC	0	0.12	0.12
CAGGACTCAAGCATTTCGCAAGCA	0	0.12	0.12
AGGACTCAAGCATTTCGCAAGCAC	0.72	0.12	0.84
ATGCTGGTTTAGATGAAGGTA	0.24	0.12	0.36
TGCTGGTTTAGATGAAGGTA	0.12	0.12	0.24
TTTAGATGAAGGTATTAAGTG	0.12	0	0.12
TTAGATGAAGGTATTAAGTGTCAA	0	0.12	0.12
AAGGATTGAACCTCAATTCGGTGG	0.12	0.12	0.24
AAGGATTGAACCTCAATTCGG	0.12	0	0.12
AGGATTGAACCTCAATTCGGTGGC	0	0.24	0.24
AGGGAAAACATGAGGTGTTTGGCG	0.24	0	0.24
AAACATGAGGTGTTTGGCGAAAT	0	0.12	0.12
GAAATGCTTGAGTCCTGTTGT	0.12	0	0.12
AAATGCTTGAGTCCTGTTGTT	2.99	2.57	5.56
AAATGCTTGAGTCCTG	0.12	0	0.12

```
        AGGTAATTCAAACTTGTCAAACAACAGGACTCAAGCATTTCGCAAGCACCTCATGTTTCTCCTTTGAAAATATTAAATTTTTTTTATGCTGGTTTAGATGAAGGTATTAAGTGTCAAGGATTGAACCTCAATTCGGTGGCACTTGAATACCATCATCTAAAGCAAGCCTCACAAAAATTTATTATTTTCAAAGGGAAAACATGAGGTGTTTGGCGAAATGCTTGAGTCCTGTTGTTTTGCAGGTTTGAATTTGCCCCA
        AGGTAATTCAAACTTGTCAAACAACAGGACTCAAGCATTTCGCAAGCACCTCATGTTTCTCCTTTGAAAATATTAAATTTTTTTTATGCTGGTTTAGATGAAGGTATTAAGTGTCAAGGATTGAACCTCAATTCGGTGGCACTTGAATACCATCATCTAAAGCAAGCCTCACAAAAATTTATTATTTTCAAAGGGAAAACATGAGGTGTTTGGCGAAATGCTTGAGTCCTGTTGTTTTGCAGGTTTGAATTTGCCCCA
-152.40 .((((((((((((((.((((((((((((((((((((((((((((((((((..((((((((((((.((((((((((.....((((.((((((((((.((((((((((((((.((((((.....))))))..)))))))))))).)))).)))))))))).)).)).....)))))))))).)))))))))))))..))))))))))))))))).))))))))))))))))))))))))).))))))))))))))).)))...   []
```

RC_mtr-MIR2592o

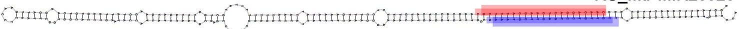

Sequence	GSM643816	GSM643815	Total
TGTCAAACAACAGGACTCAAGCA	0.12	0	0.12
TCAAACAACAGGACTCAAGCAT	0.12	0.12	0.24
CAAACAACAGGACTCAAGCATTTC	0.12	0.24	0.36
CAAACAACAGGACTCAAGCAT	0.12	0.12	0.24
CAAACAACAGGACTCAAGCATT	0.12	0.49	0.61
AAACAACAGGACTCAAGCATTTCG	0.24	0.49	0.73
AAACAACAGGACTCAAGC	0	0.12	0.12
AAACAACAGGACTCAAGCATT	0.12	0.49	0.61
AACAACAGGACTCAAGCATTTCG	0	0.12	0.12
AACAACAGGACTCAAGCATTTCGC	2.52	3.05	5.57
ACAACAGGACTCAAGCATTTCGC	0.12	0.37	0.49
ACAACAGGACTCAAGCATTTCGCA	0.12	0.49	0.61
ACAACAGGACTCAAGCATTTC	0.48	0.24	0.72
CAACAGGACTCAAGCAT	0.12	0.12	0.24
CAACAGGACTCAAGCATTT	0.12	0.12	0.24
CAACAGGACTCAAGCATT	1.44	1.83	3.27
CAACAGGACTCAAGCATTTCGCAA	0.12	0	0.12
CAACAGGACTCAAGCATTTCGCA	0.12	0	0.12
CAACAGGACTCAAGC	0.36	0	0.36
CAACAGGACTCAAGCATTTCGC	14.49	18.94	33.43
CAACAGGACTCAAGCATTTCG	11.14	18.33	29.47
AACAGGACTCAAGCATTTCGC	7.19	9.41	16.6
AACAGGACTCAAGCATTTCGCAAG	0.12	0.12	0.24
AACAGGACTCAAGCATTTCG	0.24	0	0.24
ACAGGACTCAAGCATT	0.12	0	0.12
ACAGGACTCAAGCATTTCGC	0	0.12	0.12
AAGGATTGAACCTCAATTCGGTGG	0.12	0.12	0.24
AAGGATTGAACCTCAATTCGG	0.12	0	0.12
AGGATTGAACCTCAATTCGGTGGC	0	0.24	0.24
AGGGAAAACATGAGGTGTTTGGCG	0.24	0	0.24
AAACATGAGGTGTTTGGCGAAAT	0	0.12	0.12
GAAATGCTTGAGTCCTGTTGT	0.12	0	0.12
AAATGCTTGAGTCCTGTTGTT	2.99	2.57	5.56
AAATGCTTGAGTCCTG	0.12	0	0.12

GGCTAATTCAAACTTGTCAAACAACAGGACTCAAGCATTTCGCAAGCGCCTCATGTTTCTCCTTTGAAAGTATTAAATTTTTGTTATGCTGGTTTAGATGAATGTATTAAGTGTCAAGGATTGAACCTCAATTCGGTGGCACTTGAATACCATCATCCAAAGCTAGCCTCACAGAAATTTATTATTTTCAAAGGGAAAACATGAGGTGTTTGGCGAAATGCTTGAGTCCTGTTGTTTTGCAGGTTTGAATTTATCCC

GGCTAATTCAAACTTGTCAAACAACAGGACTCAAGCATTTCGCAAGCGCCTCATGTTTCTCCTTTGAAAGTATTAAATTTTTGTTATGCTGGTTTAGATGAATGTATTAAGTGTCAAGGATTGAACCTCAATTCGGTGGCACTTGAATACCATCATCCAAAGCTAGCCTCACAGAAATTTATTATTTTCAAAGGGAAAACATGAGGTGTTTGGCGAAATGCTTGAGTCCTGTTGTTTTGCAGGTTTGAATTTATCCC

-149.50 ((..((((((((((((.((((((((((((((((((((((((((((((((((((..((((((((((((.((((((((((((...((((((((.(...((((((((.(((((..((((((((((((.((((((.....))))))..)))))))))).)))..)))))..))))))))...)))))))))))).)))))))))))).))))))))))))..)))))))))))))))))).)))))))))))))))))))))))))).)))))))))))))))...)). []

RC_mtr-MIR2592q

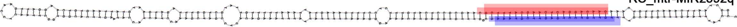

Sequence	GSM643816	GSM643815	Total
TGTCAAACAACAGGACTCAAGCA	0.12	0	0.12
TCAAACAACAGGACTCAAGCAT	0.12	0.12	0.24
CAAACAACAGGACTCAAGCAT	0.12	0.12	0.24
CAAACAACAGGACTCAAGCATT	0.12	0.49	0.61
CAAACAACAGGACTCAAGCATTTC	0.12	0.24	0.36
AAACAACAGGACTCAAGCATT	0.12	0.49	0.61
AAACAACAGGACTCAAGC	0	0.12	0.12
AAACAACAGGACTCAAGCATTTCG	0.24	0.49	0.73
AACAACAGGACTCAAGCATTTCGC	2.52	3.05	5.57
AACAACAGGACTCAAGCATTTCG	0	0.12	0.12
ACAACAGGACTCAAGCATTTCGC	0.12	0.37	0.49
ACAACAGGACTCAAGCATTTCGCA	0.12	0.49	0.61
ACAACAGGACTCAAGCATTTC	0.48	0.24	0.72
CAACAGGACTCAAGCATT	1.44	1.83	3.27
CAACAGGACTCAAGCATTTCGC	14.49	18.94	33.43
CAACAGGACTCAAGCATTT	0.12	0.12	0.24
CAACAGGACTCAAGCAT	0.12	0.12	0.24
CAACAGGACTCAAGCATTTCGCA	0.12	0	0.12
CAACAGGACTCAAGC	0.36	0	0.36
CAACAGGACTCAAGCATTTCGCAA	0.12	0	0.12
CAACAGGACTCAAGCATTTCG	11.14	18.33	29.47
AACAGGACTCAAGCATTTCG	0.24	0	0.24
AACAGGACTCAAGCATTTCGC	7.19	9.41	16.6
AACAGGACTCAAGCATTTCGCAAG	0.12	0.12	0.24
ACAGGACTCAAGCATT	0.12	0	0.12
ACAGGACTCAAGCATTTCGC	0	0.12	0.12
CAGGACTCAAGCATTTCGCAAGCA	0	0.12	0.12
AGGACTCAAGCATTTCGCAAGCAC	0.72	0.12	0.84
ATGCTGGTTTAGATGAAGGTA	0.24	0.12	0.36
TGCTGGTTTAGATGAAGGTA	0.12	0.12	0.24
TTTAGATGAAGGTATTAAGTG	0.12	0	0.12
TTAGATGAAGGTATTAAGTGTCAA	0	0.12	0.12
AAGGATTGAACCTCAATTCGGTGG	0.12	0.12	0.24
AAGGATTGAACCTCAATTCGG	0.12	0	0.12
AGGATTGAACCTCAATTCGGTGGC	0	0.24	0.24
AGGGAAAACATGAGGTGTTTGGCG	0.24	0	0.24
AAACATGAGGTGTTTGGCGAAAT	0	0.12	0.12
GAAATGCTTGAGTCCTGTTGT	0.12	0	0.12
AAATGCTTGAGTCCTGTTGTT	2.99	2.57	5.56
AAATGCTTGAGTCCTG	0.12	0	0.12

```
        AGGTAATTCAAACTTGTCAAACAACAGGACTCAAGCATTTCGCAAGCACCTCATGTTTCTCCTTTGAAAATATTAAATTTTTGTTATGCTGGTTTAGATGAAGGTATTAAGTGTCAAGGATTGAACCTCAATTCGGTGGCACTTGAATACCATCATCTAAAGCAAGCCTCACAAAAATTTATTATTTTCAAAGGGAAAACATGAGGTGTTTGGCGAAATGCTTGAGTCCTGTTGTTTTGCAGGTTTGAATTTATCCCA
        AGGTAATTCAAACTTGTCAAACAACAGGACTCAAGCATTTCGCAAGCACCTCATGTTTCTCCTTTGAAAATATTAAATTTTTGTTATGCTGGTTTAGATGAAGGTATTAAGTGTCAAGGATTGAACCTCAATTCGGTGGCACTTGAATACCATCATCTAAAGCAAGCCTCACAAAAATTTATTATTTTCAAAGGGAAAACATGAGGTGTTTGGCGAAATGCTTGAGTCCTGTTGTTTTGCAGGTTTGAATTTATCCCA
-155.50 .((.((((((((((((.((((((((((((((((((((((((((((((((((((((..((((((((((((.(((((((((((...((((.((((...((((.(((((((((.(((((((((((((.((((((.....)))))))..))))))))))).)))).))))))))).)).))...)))))))))).)))))))))))...))))))))))..)))))))))))))))))).))))))))))))))))))))))))))).)))))))))))))...))..  []
```

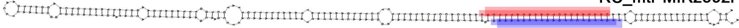

	GSM693279	GSM520637	GSM309693	GSM455965	GSM309691	GSM309692	Total
TGACAGAGAGAGAAGTGAGCAC	0.1	0	0	0	0	0	0.1
CGTGTGCTCACTCTCTTCTGT	0	0.22	0	0	0	0	0.22
TGTGCTCACTCTCTTCTGTCA	0	0	6.05	0	40.48	0	46.53
TGTGCTCACTCTCTTCTGTC	0	0	0	0.25	0	0	0.25
GTGCTCACTCTCTTCTGTC	0	0	6.05	0	0	19.98	26.03
GTGCTCACTCTCTTCTGT	0	0	6.05	0	0	19.98	26.03
GTGCTCACTCTCTTCTGTCA	0	0	181.63	0	1264.9	459.6	1906.13
TGCTCACTCTCTTCTGTCA	0	0	18.16	0	0	0	18.16
TGCTCACTCTCTTCTGTC	0	0	6.05	0	0	0	6.05
TGCTCACTCTCTTCTGT	0	0	6.05	0	0	19.98	26.03
GCTCACTCTCTTCTGTCA	0	0	6.05	0	0	19.98	26.03
CTCACTCTCTTCTGTCA	0	0	0	0	0	19.98	19.98

```
      GGAGGTGACAGAGAGAGAAGTGAGCACGCGTAGCTCCAAGCATAGGCATCATTTATGCAAGGAAACAACCACGTGTGCTCACTCTCTTCTGTCACCCTCC
-54.20 ((((((((((.((((.((((((((((((...(((..(((((((.....)))))))..))).......)))))))))))))))))))))))))).)))  []
```

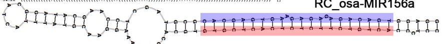

RC_osa-MIR156a

	GSM693279	GSM309692	GSM309691	GSM571078	GSM309693	GSM455965	Total
GGCTGACAGAGAGAGAAGTGAGCAC	0.1	0	0	0	0	0	0.1
GCTGACAGAGAGAGAAGTGAGC	0	19.98	30.36	0	0	0	50.34
TGACAGAGAGAGAAGTGAGCAC	0.1	0	0	0	0	0	0.1
TGTGTGCTCACTCTCTTCTG	0	0	0	0.24	0	0	0.24
TGTGCTCACTCTCTTCTGTCA	0	0	40.48	0	6.05	0	46.53
TGTGCTCACTCTCTTCTGTC	0	0	0	0	0	0.25	0.25
GTGCTCACTCTCTTCTGT	0	19.98	0	0	6.05	0	26.03
GTGCTCACTCTCTTCTGTCA	0	459.6	1264.9	0	181.63	0	1906.13
GTGCTCACTCTCTTCTGTCAG	0	0	0	0	6.05	0	6.05
GTGCTCACTCTCTTCTGTC	0	19.98	0	0	6.05	0	26.03
TGCTCACTCTCTTCTGTCA	0	0	0	0	18.16	0	18.16
TGCTCACTCTCTTCTGT	0	19.98	0	0	6.05	0	26.03
TGCTCACTCTCTTCTGTC	0	0	0	0	6.05	0	6.05
GCTCACTCTCTTCTGTCA	0	19.98	0	0	6.05	0	26.03
CTCACTCTCTTCTGTCA	0	19.98	0	0	0	0	19.98

```
      GGAGAAAGAGAGAAAGAGAGATCAAATGGCTGACAGAGAGAGAAGTGAGCACACATAGCTTCAAGCATGAGTCCATTCAGCATGCAAGAAAGTCACCATGTGTGCTCACTCTCTTCTGTCAGCCTCACCTCTCACCCCTCTCTTCCTCC
-73.60 ((((.(((((((...(((((.......((((((((((.((((.(((((((((((((.(((((..(((((...........)))))..))).))....))))))))))))))))))))))))))....)))))....))))))).))))  []
```

RC_osa-MIR156c

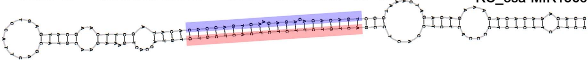

Sequence	GSM693280	GSM693279	GSM309691	GSM309692	GSM278571	GSM686039	GSM686040	GSM571077	GSM520637	GSM455965	GSM520639	GSM571078	GSM309693	Total
GCTGACAGAAAGAGAAGTGAGCAC	0.32	0.2	0	0	0	0	0	0	0	0	0	0	0	0.52
GCTGACAGAAAGAGAAGTGAGC	0	0	20.24	19.98	0	0	0	0	0	0	0	0	0	40.22
GCTGACAGAAAGAGAAGTGAGCA	0.11	0	0	0	0	0	0	0	0	0	0	0	0	0.11
TGACAGAAAGAGAAGTGAGCAC	0	0	0	0	0.31	0	0	0	0	0	0	0	0	0.31
CAGAAAGAGAAGTGAGCACGCAG	0.11	0.1	0	0	0	4.45	21.7	0.26	0	0	0	0	0	26.62
CAGAAAGAGAAGTGAGCA	0	0	0	0	0	0	0.15	0	0	0	0	0	0	0.15
CAGAAAGAGAAGTGAGCACGC	0	0	0	0	0	0.31	0.53	0	0	0	0	0	0	0.84
CAGAAAGAGAAGTGAGCACG	0	0	0	0	0	0	0.08	0	0	0	0	0	0	0.08
CAGAAAGAGAAGTGAGCACGCAGCGG	0	0	0	0	0	0	0.08	0	0	0	0	0	0	0.08
CAGAAAGAGAAGTGAGCACGCA	0	0	0	0	0	0	0.38	0	0	0	0	0	0	0.38
AGAAAGAGAAGTGAGCACGCAG	0	0	0	0	0	0.08	0.75	0	0	0	0	0	0	0.83
AGAAAGAGAAGTGAGCACGCAGC	0	0	0	0	0	0	0.08	0	0	0	0	0	0	0.08
GAAAGAGAAGTGAGCACGCAGCGG	0	0.1	0	0	0	0	0	0	0	0	0	0	0	0.1
CATCTCTCTCTCTCTCTCTCT	0	0	0	0	0	0	0	0	0.22	0	0	0	0	0.22
TCTCTCTCTCTCTCTCTCT	0	0	0	0	0	0	0	0	0	0.25	0.22	0	0	0.47
CGCCGACGGCCTCTCTTTCTT	0	0	0	0	0	0	0.08	0	0	0	0	0	0	0.08
TGTGTGCTCACTCTCTTCTG	0	0	0	0	0	0	0	0	0	0	0	0.24	0	0.24
TGTGCTCACTCTCTTCTGTC	0	0	0	0	0	0	0	0	0	0.25	0	0	0	0.25
TGTGCTCACTCTCTTCTGTCA	0	0	40.48	0	0	0	0	0	0	0	0	0	6.05	46.53
GTGCTCACTCTCTTCTGTCA	0	0	1264.9	459.6	0	0	0	0	0	0	0	0	181.63	1906.13
GTGCTCACTCTCTTCTGT	0	0	0	19.98	0	0	0	0	0	0	0	0	6.05	26.03
GTGCTCACTCTCTTCTGTC	0	0	0	19.98	0	0	0	0	0	0	0	0	6.05	26.03
GTGCTCACTCTCTTCTGTCAA	0	0	0	0	0	0	0	0	0	0	0	0	6.05	6.05
TGCTCACTCTCTTCTGTCAA	0	0	10.12	0	0	0	0	0	0	0	0	0	6.05	16.17
TGCTCACTCTCTTCTGTCA	0	0	0	0	0	0	0	0	0	0	0	0	18.16	18.16
TGCTCACTCTCTTCTGT	0	0	0	19.98	0	0	0	0	0	0	0	0	6.05	26.03
TGCTCACTCTCTTCTGTC	0	0	0	0	0	0	0	0	0	0	0	0	6.05	6.05
GCTCACTCTCTTCTGTCA	0	0	0	19.98	0	0	0	0	0	0	0	0	6.05	26.03
CTCACTCTCTTCTGTCA	0	0	0	19.98	0	0	0	0	0	0	0	0	0	19.98
CTCACTCTCTTCTGTCAA	0	0	10.12	0	0	0	0	0	0	0	0	0	0	10.12

AGCTGACAGAAAGAGAAGTGAGCACGCAGCGGCAGCTGTATCATCATCATCATTTCATCTCTCTCTCTCTCTCTCTTCCATGGCCGCCGACGGCCTCTCTTTCTTTCTATCTAGCTATCCTGTCGAAGGGAAGATTGATAGATCGATGCAGTCTGGCCGCTGTGTGCTCACTCTCTTCTGTCAACT

-80.36 ((.((((((.(((((.((((((((((((((((((.(((((((((((((((.................(((.((((((((...(((((....)))))............................)))))))))).)))).))).))))))))...))))))))))))))))))))))))))))))).)) []

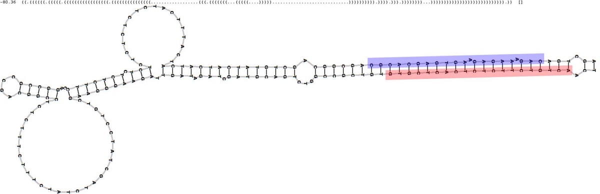

RC_osa-MIR156f

	GSM309692	GSM309691	GSM693279	GSM278571	GSM520635	GSM571078	GSM309693	GSM455965	Total
GCTGACAGAGAGAGAAGTGAGC	19.98	30.36	0	0	0	0	0	0	50.34
TGACAGAGAGAGAAGTGAGCAC	0	0	0.1	0	0	0	0	0	0.1
CAGAGAGAGAAGTGAGCACGCA	0	0	0	0.31	0	0	0	0	0.31
GAAGTGAGCACGCAACGGCAAGCT	0	0	0	0	0.33	0	0	0	0.33
TGTGTGCTCACTCTCTTCTG	0	0	0	0	0	0.24	0	0	0.24
TGTGCTCACTCTCTTCTGTCA	0	40.48	0	0	0	0	6.05	0	46.53
TGTGCTCACTCTCTTCTGTC	0	0	0	0	0	0	0	0.25	0.25
GTGCTCACTCTCTTCTGTC	19.98	0	0	0	0	0	6.05	0	26.03
GTGCTCACTCTCTTCTGTCA	459.6	1264.9	0	0	0	0	181.63	0	1906.13
GTGCTCACTCTCTTCTGTCAG	0	0	0	0	0	0	6.05	0	6.05
GTGCTCACTCTCTTCTGT	19.98	0	0	0	0	0	6.05	0	26.03
TGCTCACTCTCTTCTGT	19.98	0	0	0	0	0	6.05	0	26.03
TGCTCACTCTCTTCTGTCA	0	0	0	0	0	0	18.16	0	18.16
TGCTCACTCTCTTCTGTC	0	0	0	0	0	0	6.05	0	6.05
GCTCACTCTCTTCTGTCA	19.98	0	0	0	0	0	6.05	0	26.03
CTCACTCTCTTCTGTCA	19.98	0	0	0	0	0	0	0	19.98

```
       AGCTGACAGAGAGAGAAGTGAGCACGCAACGGCAAGCTGCGTCCGTCGTCTTCTTCGTCGCAACAGGTAATTCAGATGCAGTCTGCCCGCTGTGTGCTCACTCTCTTCTGTCAGCC
-62.83 .((((((((.((((.((((((((((((.(((((..(((((((((.((.......)).))..............)))))))..))).)).)))))))))))))))))))))))))).  []
```

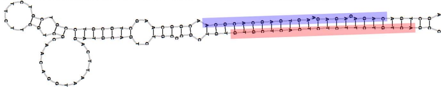

RC_osa-MIR156g

Sequence	GSM309693	GSM309691	GSM409321	GSM309692	GSM520637	GSM571077	GSM686040	Total
CAGAGCTCCCTTCAATCCAA	6.05	10.12	0	0	0	0	0	16.17
CAGAGCTCCCTTCAATCCAAA	6.05	212.5	7.33	19.98	0	0	0	245.86
CAGAGCTCCCTTCAATC	6.05	0	0	0	0	0	0	6.05
CAGAGCTCCCTTCAATCCA	6.05	10.12	0	0	0	0	0	16.17
AGAGCTCCCTTCAATCCAA	6.05	10.12	0	19.98	0	0	0	36.15
AGAGCTCCCTTCAATCCAAA	6.05	141.67	0	19.98	0	0	0	167.7
AGAGCTCCCTTCAATCCAAAG	0	10.12	0	0	0	0	0	10.12
GAGCTCCCTTCAATCCA	6.05	0	0	0	0	0	0	6.05
AGCTCCCTTCAATCCAAA	0	0	0	19.98	0	0	0	19.98
ATGCAGCTCCTGGGGCATG	6.05	0	0	0	0	0	0	6.05
GCAGCTCCTGGGGCATGCAA	6.05	70.83	0	19.98	0	0	0	96.86
GCAGCTCCTGGGGCATGCAAG	0	10.12	0	0	0	0	0	10.12
CAGCTCCTGGGGCATGCAA	0	10.12	0	0	0	0	0	10.12
TCAGATCTCGAGCTGAATGAT	0	0	0	0	0.44	0	0	0.44
TGATGAACATGGAGTAGGCTAA	0	0	0	0	0	0.26	0	0.26
CATGGAGTAGGCTAACGGGATC	0	0	0	0	0	0	0.08	0.08
CCCAGCAGCTCAATCGACCC	0	10.12	0	0	0	0	0	10.12
TTTTTGGACCGAAAGGAGCTCA	0	10.12	0	19.98	0	0	0	30.1
TTTGGACCGAAAGGAGCTCAA	6.05	10.12	0	0	0	0	0	16.17

```
        ACAAAAGATGCAGAGCTCCCTTCAATCCAAAGAGGGTCCATGTAGGGTTGATGCAGCTCCTGGGGCATGCAAGCCATAAGCCTATCCCAACAACGAGTACTCCATCATTTTCCTATCTACACACATACTATTAGTATAAATTGGAGTAGTTTCTTTCAGATCTCGAGCTGAATGATGAACATGGAGTAGGCTAACGGGATCCATGACCCAGCAGCTCAATCGACCCACAGCAACACCCCTTTTTGGACCGAAAGGAGCTCAACGTCCACAAC
-96.09  ......((((..(((((((.(((..(((((((((((....(((.((((((((..((((.(((((.((((....((...((((((..(((............((((((((..((((((........((((((..((.....)).))))))........))))....))..))))))))....)))..))))))....))...)))).)))))).))))..)))))))))))).......)))))))))))..))).)))))))..))))......  []
```

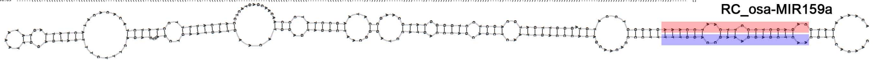

```
                 AGTGAAACTGTCACAGCATGATC
                  TGAAACTGTCACAGCATGATCT
                  TGAAACTGTCACAGCATGATCTA
                  TGAAACTGTCACAGCATGA
                   GAAACTGTCACAGCATGATCTA
                                          ATCACGTACTCAGAAGAAGCC
                                                                                                       AGATCATGCTGGCAGCTTCA
                                                                                                         ATCATGCTGGCAGCTTCACG
                                                                                                         ATCATGCTGGCAGCTTCA
       GTGCCCAACAGGGAAGGAGTGAAACTGTCACAGCATGATCTAACCATCACGTACTCAGAAGAAGCCTCAGCTAGCCGCCTTCCTGTTCGAAACCAGCGAGCTGAGCTGATTTCTTGTCTGCAAGTTAGATCATGCTGGCAGCTTCACGCTTTCTCTTGGGCAC
-73.94 ((((((..(((((((.(((((.((((..((((((((((((((...........((((((((..((((((((((((.(((......)))....))).))).)))))))))))..))))...)))))))))))))))))))).)))).)))))))))))))))) []
```

GSM571078	GSM686040	GSM693280	GSM693279	GSM686039	GSM520640	GSM309693	GSM309691	Total
0.24	0	0	0	0	0	0	0	0.24
0	0.08	0	0.1	0.08	0	0	0	0.26
0	0.3	0.11	0.4	0.86	0	0	0	1.67
0	0	0	0	0	0.2	0	0	0.2
0	0	0	0.1	0	0	0	0	0.1
0	0.08	0	0	0	0	0	0	0.08
0	0	0	0	0	0	6.05	50.6	56.65
0	0	0	0	0.08	0	0	0	0.08
0	0	0	0	0	0	6.05	0	6.05

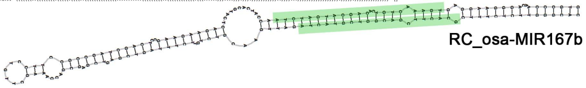

RC_osa-MIR167b

Sequence	GSM455965	GSM686040	GSM693279	GSM309691	GSM309693	GSM309692	Total
TGAAACTGCACAGCATGATCTGA	0.25	0.08	0	0	0	0	0.33
TGAAACTGCACAGCATGATCTGAT	0	0.08	0	0	0	0	0.08
TGAAACTGCACAGCATGATCTG	0	0.08	0	0	0	0	0.08
AAGCACTCATCAGATCATGCTGGC	0	0	0.1	0	0	0	0.1
ATCAGATCATGCTGGCAGCTT	0	0	0	10.12	0	0	10.12
ATCAGATCATGCTGGCAGCTTCA	0	0	0	60.72	6.05	19.98	86.75
ATCAGATCATGCTGGCAGCTTCAGC	0	0	0	0	0	19.98	19.98
TCAGATCATGCTGGCAGCTTCA	0	0	0	0	6.05	0	6.05
CAGATCATGCTGGCAGCTTCA	0	0	0	465.48	6.05	19.98	491.51
CAGATCATGCTGGCAGCTTC	0	0	0	10.12	0	0	10.12
AGATCATGCTGGCAGCTTCA	0	0	0	50.6	6.05	0	56.65
ATCATGCTGGCAGCTTCA	0	0	0	0	6.05	0	6.05

```
      CACAAGCAGATGAAACTGCACAGCATGATCTGATCTTTGTTTTGGCAGTCAATTCTGCCCTCACCTAATAAGCACTCATCAGATCATGCTGGCAGCTTCAGCTCCTAATG
-44.50 ....((.((.((((.((((.(((((((((((((...(((((.(((((.....)))))..........)))))...))))))))))))))).)))).)).))....  []
```

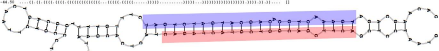

RC_osa-MIR167d

	GSM520635	GSM693280	GSM309692	GSM309693	GSM309691	Total
GATGAAACTGCCAGATGATCTGAT	0.16	0	0	0	0	0.16
TGAAACTGCCAGATGATCTGA	0	0.11	0	0	0	0.11
ATCAGATCATGCTGGCAGCTTCA	0	0	19.98	6.05	60.72	86.75
ATCAGATCATGCTGGCAGCTT	0	0	0	0	10.12	10.12
TCAGATCATGCTGGCAGCTTCA	0	0	0	6.05	0	6.05
CAGATCATGCTGGCAGCTTCAT	0	0	0	6.05	0	6.05
CAGATCATGCTGGCAGCTTCA	0	0	19.98	6.05	465.48	491.51
CAGATCATGCTGGCAGCTTC	0	0	0	0	10.12	10.12
AGATCATGCTGGCAGCTTCA	0	0	0	6.05	50.6	56.65
AGATCATGCTGGCAGCTTCAT	0	0	0	6.05	0	6.05
ATCATGCTGGCAGCTTCATCC	0	0	0	6.05	0	6.05
ATCATGCTGGCAGCTTCAT	0	0	0	6.05	0	6.05
ATCATGCTGGCAGCTTCA	0	0	0	6.05	0	6.05

CATGAGAAGATGAAACTGCCAGATGATCTGATGCATGTCTCTAGGTTTTGTAAATCATCATCAGCTAGAGAACTACTGTGATCAGATCATGCTGGCAGCTTCATCCACTTGTG

-49.60 ((..((..((((((.((((((((((((((((((((..(((((((....((........))....)))))))......))).)))))))))).)))))))).))))))..))..)) []

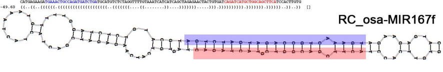

AGCCAAGGACAACTTGCCGGA

	GSM520639	GSM309692	GSM309691	GSM309693	Total
	0.44	0	0	0	0.44
ACCGGCAAGTCATCCTTGG	0	19.98	10.12	0	30.1
ACCGGCAAGTCATCCTTGGC	0	19.98	0	0	19.98
CCGGCAAGTCATCCTTGGCT	0	19.98	30.36	0	50.34
CCGGCAAGTCATCCTTG	0	19.98	0	0	19.98
CCGGCAAGTCATCCTTGGC	0	19.98	0	0	19.98
CGGCAAGTCATCCTTGGCT	0	19.98	0	0	19.98
CGGCAAGTCATCCTTGGCTG	0	79.93	505.96	6.05	591.94
CGGCAAGTCATCCTTGG	0	19.98	0	0	19.98
CGGCAAGTCATCCTTGGCTGC	0	0	10.12	0	10.12
GGCAAGTCATCCTTGGCTG	0	19.98	0	0	19.98
GGCAAGTCATCCTTGGC	0	0	10.12	0	10.12
GGCAAGTCATCCTTGGCTGCA	0	0	10.12	0	10.12
GCAAGTCATCCTTGGCT	0	0	0	6.05	6.05
GCAAGTCATCCTTGGCTG	0	19.98	10.12	0	30.1
CAAGTCATCCTTGGCTG	0	0	0	6.05	6.05

GAACAAGACGTAGCCAAGGACAACTTGCCGGAAAATGTCAGCATGGATCTAAAACAGTTGGGGGCACATATAGTACCTTGAAACATGCATACACGTACCGGCAAGTCATCCTTGGCTGCATTCCGTTC

-52.12 ((((..((.((((((((((..((((((((....(((..(((((............((.((((.((.....)).)))).)).)))))..)))....))))))))..)))))))))).))..)))) []

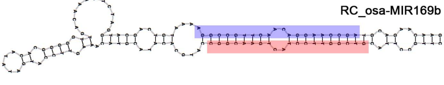

	GSM520639	GSM520637	GSM520638	GSM520640	GSM455965	GSM278572	GSM686039	GSM686040	GSM309691	GSM309692	GSM309693	GSM520635	Total
TAGGTGTAGCCAAGGACAGACT	0.66	0	0	0	0	0	0	0	0	0	0	0	0.66
AGGTGTAGCCAAGGACAGACTT	0	0.22	0.41	0.2	0.25	0.33	0	0	0	0	0	0	1.41
AGGTGTAGCCAAGGACAGACT	0	0	0	0.2	0	0	0	0	0	0	0	0	0.2
GGTGTAGCCAAGGACAGACTTGC	0	0	0	0	0	0	0.31	0.08	0	0	0	0	0.39
GGTGTAGCCAAGGACAGACTTGCC	0	0	0	0	0	0	0.08	0	0	0	0	0	0.08
TGTAGCCAAGGACAGACTTGCC	0	0	0.2	0.2	0.25	0	0.23	0	0	0	0	0	0.88
TGTAGCCAAGGACAGACTTGC	0	0	0.81	0.39	0	0	0	0	0	0	0	0	1.2
TGTAGCCAAGGACAGACTTG	0	0	0	0	0	0	0	0.08	0	0	0	0	0.08
TGTAGCCAAGGACAGACTTGCCGGC	0	0	0	0	0	0	0	0.08	0	0	0	0	0.08
TAGCCAAGGACAGACTTGCCGGCT	0	0	0	0.2	0	0	0	0	0	0	0	0	0.2
TAGCCAAGGACAGACTTGCCGG	0	0	0.2	0	0	0	0	0	0	0	0	0	0.2
TAGCCAAGGACAGACTTGCCG	0.66	0	0	0	0	0	0	0	0	0	0	0	0.66
AGCCAAGGACAGACTTGCCGGC	0	0	0	0	0	0	4.53	2.11	0	0	0	0	6.64
AGCCAAGGACAGACTTGCCGG	0	0	0	0	0	0	0.16	0.08	0	0	0	0	0.24
AGCCAAGGACAGACTTGCC	0	0	0	0	0	0	0	0.08	0	0	0	0	0.08
AGCCAAGGACAGACTTGCCG	0	0	0	0	0	0	0	0.08	0	0	0	0	0.08
AGCCAAGGACAGACTTGCCGGCT	0	0	0	0	0	0	0.08	0	0	0	0	0	0.08
GCCAAGGACAGACTTGCCGGC	0	0	0	0	0	0	0.08	0	0	0	0	0	0.08
GCCAAGGACAGACTTGCCGGCTAA	0	0	0	0	0	0	0.08	0	0	0	0	0	0.08
CCAAGGACAGACTTGCCGGCTAAC	0	0	0	0	0	0	0.08	0	0	0	0	0	0.08
CAAGGACAGACTTGCCGGCTAA	0	0	0	0	0	0	0	0.08	0	0	0	0	0.08
AGGACAGACTTGCCGGCTAA	0	0	0	0	0	0	0.08	0	0	0	0	0	0.08
GACAGACTTGCCGGCTAACT	0	0	0	0	0	0	0	0.08	0	0	0	0	0.08
TGCCGGCTAACTCCAAGGCGC	0	0	0	0	0	0	0.08	0	0	0	0	0	0.08
GCCGGCAAGTCATCCTTGGCT	0	0	0	0	0	0	0	0	20.24	0	0	0	20.24
CCGGCAAGTCATCCTTGGCT	0	0	0	0	0	0	0	0	30.36	19.98	0	0	50.34
CCGGCAAGTCATCCTTGGC	0	0	0	0	0	0	0	0	0	19.98	0	0	19.98
CCGGCAAGTCATCCTTG	0	0	0	0	0	0	0	0	0	19.98	0	0	19.98
CGGCAAGTCATCCTTGGCTG	0	0	0	0	0	0	0	0	505.96	79.93	6.05	0	591.94
CGGCAAGTCATCCTTGG	0	0	0	0	0	0	0	0	0	19.98	0	0	19.98
CGGCAAGTCATCCTTGGCTGC	0	0	0	0	0	0	0	0	10.12	0	0	0	10.12
CGGCAAGTCATCCTTGGCT	0	0	0	0	0	0	0	0	0	19.98	0	0	19.98
GGCAAGTCATCCTTGGCTG	0	0	0	0	0	0	0	0	0	19.98	0	0	19.98
GGCAAGTCATCCTTGGCTGCA	0	0	0	0	0	0	0	0	10.12	0	0	0	10.12
GGCAAGTCATCCTTGGC	0	0	0	0	0	0	0	0	10.12	0	0	0	10.12
GCAAGTCATCCTTGGCTG	0	0	0	0	0	0	0	0	10.12	19.98	0	0	30.1
GCAAGTCATCCTTGGCT	0	0	0	0	0	0	0	0	0	0	6.05	0	6.05
CAAGTCATCCTTGGCTGCATC	0	0	0	0	0	0	0.08	0	0	0	0	0	0.08
CAAGTCATCCTTGGCTG	0	0	0	0	0	0	0	0	0	0	6.05	0	6.05
GTCATCCTTGGCTGCATCCCGT	0	0	0	0	0	0	0	0	0	0	0	0.16	0.16

```
       GAGCTAGGTGTAGCCAAGGACAGACTTGCCGGCTAACTCCAAGGCGCTTCAGCAAAGCTGAGATTCCCCAATACCAGGAGCCGGCAAGTCATCCTTGGCTGCATCCCGTTC
-57.75 ((((..(((((((((((((..(((((((((((........((...((((((...))))))...))............))))))))))).)))))))))))))..))))   []
```

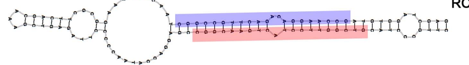

RC_osa-MIR169c

Sequence	GSM693279	GSM309691	GSM309693	GSM309692	GSM329297	GSM686039	GSM409324	Total
ATCACTCAGGCTAGCCAAGGAGAC	0.1	0	0	0	0	0	0	0.1
AGGCTAGCCAAGGAGACTGCC	0	10.12	0	0	0	0	0	10.12
GCTAGCCAAGGAGACTGC	0	0	6.05	19.98	0	0	0	26.03
GCTAGCCAAGGAGACTGCC	0	10.12	0	0	0	0	0	10.12
GCTAGCCAAGGAGACTGCCCAT	0	10.12	0	0	0	0	0	10.12
TAGCCAAGGAGACTGCCCA	0	0	0	0	16.38	0	0	16.38
AGCCAAGGAGACTGCCCAT	0	10.12	0	0	0	0	0	10.12
AGGAGACTGCCCATGAAACCTGCT	0	0	0	0	0	0.08	0	0.08
ACAGGCAAGTCATCCTTGG	0	10.12	6.05	0	0	0	0	16.17
ACAGGCAAGTCATCCTTGGCT	0	131.55	6.05	19.98	0	0	0	157.58
ACAGGCAAGTCATCCTTGGC	0	20.24	6.05	0	0	0	0	26.29
CAGGCAAGTCATCCTTGGCT	0	10.12	0	0	0	0	0	10.12
CAGGCAAGTCATCCTTGGCTA	0	0	0	0	0	0	9.04	9.04
AGGCAAGTCATCCTTGG	0	0	6.05	0	0	0	0	6.05
AGGCAAGTCATCCTTGGCT	0	0	6.05	19.98	0	0	0	26.03
AGGCAAGTCATCCTTGGCTA	0	1801.22	205.84	279.75	0	0	0	2286.81
AGGCAAGTCATCCTTGGCTACC	0	10.12	0	0	0	0	0	10.12
GGCAAGTCATCCTTGGC	0	10.12	0	0	0	0	0	10.12
GGCAAGTCATCCTTGGCTA	0	0	0	19.98	0	0	0	19.98
GCAAGTCATCCTTGGCTAC	0	0	0	19.98	0	0	0	19.98
GCAAGTCATCCTTGGCT	0	0	6.05	0	0	0	0	6.05
CAAGTCATCCTTGGCTA	0	10.12	6.05	19.98	0	0	0	36.15

ATCACTCAGGCTAGCCAAGGAGACTGCCCATGAAACCTGCTGAAAGGATCATTTTCTGAATAATAATTAATGATCCTTACACACAAGGACACAGGCAAGTCATCCTTGGCTACCAGAGCTAA

-47.86 ....(((.((.((((((((((((((((..((...(((..((.((((((((((...............)))))))))).))....))).))..)))).))).))))))))))).))).... []

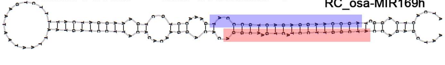

RC_osa-MIR169h

Sequence	GSM309691	GSM309692	GSM309693	GSM329297	GSM409324	Total
ACTAGCCAAGGAGACTGCCT	40.48	19.98	6.05	0	0	66.51
CTAGCCAAGGAGACTGCCT	0	19.98	6.05	0	0	26.03
TAGCCAAGGAGACTGCCT	0	0	0	45.03	0	45.03
AGCCAAGGAGACTGCCT	0	19.98	0	0	0	19.98
ACAGGCAAGTCATCCTTGGC	20.24	0	6.05	0	0	26.29
ACAGGCAAGTCATCCTTGG	10.12	0	6.05	0	0	16.17
ACAGGCAAGTCATCCTTGGCT	131.55	19.98	6.05	0	0	157.58
CAGGCAAGTCATCCTTGGCTA	0	0	0	0	9.04	9.04
CAGGCAAGTCATCCTTGGCT	10.12	0	0	0	0	10.12
AGGCAAGTCATCCTTGG	0	0	6.05	0	0	6.05
AGGCAAGTCATCCTTGGCTA	1801.22	279.75	205.84	0	0	2286.81
AGGCAAGTCATCCTTGGCT	0	19.98	6.05	0	0	26.03
AGGCAAGTCATCCTTGGCTACC	10.12	0	0	0	0	10.12
GGCAAGTCATCCTTGGCTA	0	19.98	0	0	0	19.98
GGCAAGTCATCCTTGGC	10.12	0	0	0	0	10.12
GCAAGTCATCCTTGGCTAC	0	19.98	0	0	0	19.98
GCAAGTCATCCTTGGCT	0	0	6.05	0	0	6.05
CAAGTCATCCTTGGCTA	10.12	19.98	6.05	0	0	36.15

```
       CGACTCAGACTAGCCAAGGAGACTGCCTATGAACCAGTTCAAGGCTCATTTTCTGAATGATCCTCTACACAAGGACACAGGCAAGTCATCCTTGGCTACCAGAGCTAC
-38.10 ...(((....(((((((((((((((((.((..((.((...(((.((((((...)))))).)))..))....)).)).))))).))).))))))))))...)))....   []
```

RC_osa-MIR169i

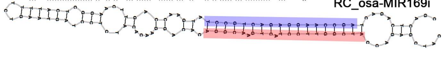

Sequence	GSM309691	GSM309692	GSM309693	GSM329297	GSM409324	Total
AGGCTAGCCAAGGAGACTGCC	10.12	0	0	0	0	10.12
AGGCTAGCCAAGGAGACTGCCT	10.12	0	0	0	0	10.12
GCTAGCCAAGGAGACTGCCT	80.95	19.98	6.05	0	0	106.98
GCTAGCCAAGGAGACTGCC	10.12	0	0	0	0	10.12
GCTAGCCAAGGAGACTGC	0	19.98	6.05	0	0	26.03
CTAGCCAAGGAGACTGCCT	0	19.98	6.05	0	0	26.03
TAGCCAAGGAGACTGCCT	0	0	0	45.03	0	45.03
AGCCAAGGAGACTGCCT	0	19.98	0	0	0	19.98
ACAGGCAAGTCATCCTTGG	10.12	0	6.05	0	0	16.17
ACAGGCAAGTCATCCTTGGCT	131.55	19.98	6.05	0	0	157.58
ACAGGCAAGTCATCCTTGGC	20.24	0	6.05	0	0	26.29
CAGGCAAGTCATCCTTGGCT	10.12	0	0	0	0	10.12
CAGGCAAGTCATCCTTGGCTA	0	0	0	0	9.04	9.04
AGGCAAGTCATCCTTGGCTA	1801.22	279.75	205.84	0	0	2286.81
AGGCAAGTCATCCTTGGCT	0	19.98	6.05	0	0	26.03
AGGCAAGTCATCCTTGGCTACC	10.12	0	0	0	0	10.12
AGGCAAGTCATCCTTGG	0	0	6.05	0	0	6.05
GGCAAGTCATCCTTGGCTA	0	19.98	0	0	0	19.98
GGCAAGTCATCCTTGGC	10.12	0	0	0	0	10.12
GCAAGTCATCCTTGGCTAC	0	19.98	0	0	0	19.98
GCAAGTCATCCTTGGCT	0	0	6.05	0	0	6.05
CAAGTCATCCTTGGCTA	10.12	19.98	6.05	0	0	36.15

```
       GCCGCTCAGGCTAGCCAAGGAGACTGCCTGTGAGATCACATATGCAGAAAGCAGCTAGAAATGCATCTTCTTCTACTGCACATGAGCAGAGACACAGGCAAGTCATCCTTGGCTACCAGATGCGA
-53.50 ..((((.((.((((((((((((((((((((...((.(((.((((..((.((..(((......))).)).)).))))).)))....))..))))))))).))).))))))))))).)).))).  []
```

RC_osa-MIR169j

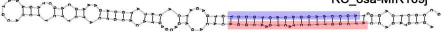

	GSM309691	GSM309693	GSM309692	GSM329297	GSM409324	Total
AGGCTAGCCAAGGAGACTGCC	10.12	0	0	0	0	10.12
GCTAGCCAAGGAGACTGCCCAT	10.12	0	0	0	0	10.12
GCTAGCCAAGGAGACTGCC	10.12	0	0	0	0	10.12
GCTAGCCAAGGAGACTGC	0	6.05	19.98	0	0	26.03
TAGCCAAGGAGACTGCCCA	0	0	0	16.38	0	16.38
AGCCAAGGAGACTGCCCAT	10.12	0	0	0	0	10.12
ACAGGCAAGTCATCCTTGGCT	131.55	6.05	19.98	0	0	157.58
ACAGGCAAGTCATCCTTGG	10.12	6.05	0	0	0	16.17
ACAGGCAAGTCATCCTTGGC	20.24	6.05	0	0	0	26.29
CAGGCAAGTCATCCTTGGCTA	0	0	0	0	9.04	9.04
CAGGCAAGTCATCCTTGGCT	10.12	0	0	0	0	10.12
AGGCAAGTCATCCTTGGCT	0	6.05	19.98	0	0	26.03
AGGCAAGTCATCCTTGGCTA	1801.22	205.84	279.75	0	0	2286.81
AGGCAAGTCATCCTTGG	0	6.05	0	0	0	6.05
GGCAAGTCATCCTTGGCTAT	30.36	0	19.98	0	0	50.34
GGCAAGTCATCCTTGGCTA	0	0	19.98	0	0	19.98
GGCAAGTCATCCTTGGC	10.12	0	0	0	0	10.12
GCAAGTCATCCTTGGCT	0	6.05	0	0	0	6.05
GCAAGTCATCCTTGGCTAT	0	6.05	19.98	0	0	26.03
CAAGTCATCCTTGGCTA	10.12	6.05	19.98	0	0	36.15
CAAGTCATCCTTGGCTAT	0	6.05	0	0	0	6.05

CCACTCAGGCTAGCCAAGGAGACTGCCCATGAACCAGCTTAAAGGATCATTAAATTGCTAATCCTTCAGGGAGGACACAGGCAAGTCATCCTTGGCTATCAGAGATAA

-40.20 ...(((.((.(((((((((((((((((..((..((..(((.((((((((......))...)))))).)))..)).))..)))).))).))))))))))))))).))).... []

RC_osa-MIR169l

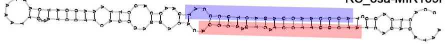

	GSM309693	GSM309691	GSM309692	GSM329297	GSM686039	GSM693279	GSM409324	Total
GCTAGCCAAGGAGACTGC	6.05	0	19.98	0	0	0	0	26.03
GCTAGCCAAGGAGACTGCC	0	10.12	0	0	0	0	0	10.12
GCTAGCCAAGGAGACTGCCT	6.05	80.95	19.98	0	0	0	0	106.98
CTAGCCAAGGAGACTGCCT	6.05	0	19.98	0	0	0	0	26.03
TAGCCAAGGAGACTGCCT	0	0	0	45.03	0	0	0	45.03
AGCCAAGGAGACTGCCTGTACATA	0	0	0	0	0.08	0	0	0.08
AGCCAAGGAGACTGCCT	0	0	19.98	0	0	0	0	19.98
AGGAGACTGCCTGTACATATATAT	0	0	0	0	0	0.1	0	0.1
ACAGGCAAGTCATCCTTGGC	6.05	20.24	0	0	0	0	0	26.29
ACAGGCAAGTCATCCTTGGCT	6.05	131.55	19.98	0	0	0	0	157.58
ACAGGCAAGTCATCCTTGG	6.05	10.12	0	0	0	0	0	16.17
CAGGCAAGTCATCCTTGGCT	0	10.12	0	0	0	0	0	10.12
CAGGCAAGTCATCCTTGGCTA	0	0	0	0	0	0	9.04	9.04
AGGCAAGTCATCCTTGGCTACC	0	10.12	0	0	0	0	0	10.12
AGGCAAGTCATCCTTGGCTA	205.84	1801.22	279.75	0	0	0	0	2286.81
AGGCAAGTCATCCTTGGCT	6.05	0	19.98	0	0	0	0	26.03
AGGCAAGTCATCCTTGG	6.05	0	0	0	0	0	0	6.05
GGCAAGTCATCCTTGGCTA	0	0	19.98	0	0	0	0	19.98
GGCAAGTCATCCTTGGC	0	10.12	0	0	0	0	0	10.12
GCAAGTCATCCTTGGCT	6.05	0	0	0	0	0	0	6.05
GCAAGTCATCCTTGGCTAC	0	0	19.98	0	0	0	0	19.98
CAAGTCATCCTTGGCTA	6.05	10.12	19.98	0	0	0	0	36.15

[illegible]ACATATATATATAAATATACAGGCAAGTCATCCTTGGCTACCAGGACTCA

-50.60 [illegible] []

RC_osa-MIR169m

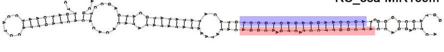

	GSM571077	GSM693279	GSM520638	GSM571078	GSM520639	GSM693280	GSM520640	GSM409324	GSM520635	GSM455965	GSM329297	GSM407072	GSM407071	Run1	GSM309692	Run2	GSM686040	GSM686039	GSM278533	GSM455964	GSM520637	GSM520636	GSM455963	GSM409316	GSM409317	GSM409313	GSM409315	GSM409318	GSM278572	GSM329296	GSM409322	GSM409321	GSM329298	GSM329299	GSM309691	GSM278534	GSM409323	GSM309693	GSM278571	GSM455962	GSM278535	GSM409320	GSM409314	GSM520634	Total
AGCAGGATGCAGCCAAGGATG	0.26	0	0	0	0	0	0	0	0	0	0	0	0	0	0	0	0	0	0	0	0	0	0	0	0	0	0	0	0	0	0	0	0	0	0	0	0	0	0	0	0	0	0	0	0.26
GCAGGATGCAGCCAAGGATGA	0	0.5	0	0	0	0	0	0	0	0	0	0	0	0	0	0	0	0	0	0	0	0	0	0	0	0	0	0	0	0	0	0	0	0	0	0	0	0	0	0	0	0	0	0	0.5
CAGGATGCAGCCAAGGATGACT	0	0	0.2	0	0	0	0	0	0	0	0	0	0	0	0	0	0	0	0	0	0	0	0	0	0	0	0	0	0	0	0	0	0	0	0	0	0	0	0	0	0	0	0	0	0.2
CAGGATGCAGCCAAGGATGAC	0.53	0.3	0	0.24	0	0	0	0	0	0	0	0	0	0	0	0	0	0	0	0	0	0	0	0	0	0	0	0	0	0	0	0	0	0	0	0	0	0	0	0	0	0	0	0	1.07
AGGATGCAGCCAAGGATGACT	0.53	0.4	0.81	0.73	13.5	0.11	0.79	18.07	0	0.99	0	0	0	0	0	0	0	0	0	0	0	0	0	0	0	0	0	0	0	0	0	0	0	0	0	0	0	0	0	0	0	0	0	0	35.93
AGGATGCAGCCAAGGATGAC	0	0	0	0	0	0	0	9.04	0	0	0	0	0	0	0	0	0	0	0	0	0	0	0	0	0	0	0	0	0	0	0	0	0	0	0	0	0	0	0	0	0	0	0	0	9.04
AGGATGCAGCCAAGGATGACTTGCC	0	0	0	0	0	0	0	0	0.16	0	0	0	0	0	0	0	0	0	0	0	0	0	0	0	0	0	0	0	0	0	0	0	0	0	0	0	0	0	0	0	0	0	0	0	0.16
GGATGCAGCCAAGGATGACT	0	0	0.41	0	0	0	0.2	0	0	0	0	0	0	0	0	0	0	0	0	0	0	0	0	0	0	0	0	0	0	0	0	0	0	0	0	0	0	0	0	0	0	0	0	0	0.61
GGATGCAGCCAAGGATGAC	0.26	0	0	0	0	0	0	0	0	0	0	0	0	0	0	0	0	0	0	0	0	0	0	0	0	0	0	0	0	0	0	0	0	0	0	0	0	0	0	0	0	0	0	0	0.26
ATGCAGCCAAGGATGACTTGC	0	0	0	0	0.44	0	0.2	0	0	0	0	0	0	0	0	0	0	0	0	0	0	0	0	0	0	0	0	0	0	0	0	0	0	0	0	0	0	0	0	0	0	0	0	0	0.64
ATGCAGCCAAGGATGACTTGCCGG	0	0	0	0	0	0	0.2	0	0	0	0	0	0	0	0	0	0	0	0	0	0	0	0	0	0	0	0	0	0	0	0	0	0	0	0	0	0	0	0	0	0	0	0	0	0.2
TGCAGCCAAGGATGACTT	0	0	0	0	0	0	0	0	0	0	4.09	0	0	0	0	0	0	0	0	0	0	0	0	0	0	0	0	0	0	0	0	0	0	0	0	0	0	0	0	0	0	0	0	0	4.09
TGCAGCCAAGGATGACTTGCC	0.26	0.1	0.41	0.49	0.44	0	0.2	0	0	0.74	0	16.5	28.16	28.16	19.98	16.5	0	0	0	0	0	0	0	0	0	0	0	0	0	0	0	0	0	0	0	0	0	0	0	0	0	0	0	0	111.94
TGCAGCCAAGGATGACTTGCCG	0.26	0	0	0	0	0	0	0	0	0	0	0	0	0	0	0	0	0	0	0	0	0	0	0	0	0	0	0	0	0	0	0	0	0	0	0	0	0	0	0	0	0	0	0	0.26
GCAGCCAAGGATGACTTGCCG	1.58	0.1	0	0.98	0	0	0	0	0	0	0	8.25	0	0	0	8.25	0.83	0.23	0	0	0	0	0	0	0	0	0	0	0	0	0	0	0	0	0	0	0	0	0	0	0	0	0	0	20.22
GCAGCCAAGGATGACTTGCCGGC	0	0	0	0	0	0	0	0	0	0.25	0	0	0	0	0	0	0	0	0	0	0	0	0	0	0	0	0	0	0	0	0	0	0	0	0	0	0	0	0	0	0	0	0	0	0.25
CAGCCAAGGATGACTTGCCGG	274.46	32.29	112.37	480.43	518.47	5.28	229.29	6127.15	1.97	110.19	13019.07	1016.88	1436.34	1436.34	0	1016.88	17.26	25.46	19.37	280.6	6.99	3.27	381.91	13.04	70.18	30.16	1356.36	1358.3	5.32	4.07	294.07	569.83	38.06	10.91	0	105.97	91.87	0	0.92	484.57	99.09	0	0	0	31020.69
CAGCCAAGGATGACTTGCC	1.06	0.1	1.22	1.71	0.22	0.11	1.78	0	0	0	167.86	8.25	0	0	19.98	8.25	0.3	0.23	0	0	0	0	0	0	0	0	0	0	0	8.15	0	0	3.46	0	0	0	0	6.05	0	0	0	0	0	0	228.73
CAGCCAAGGATGACTTGCCGGC	1.58	0.1	0	5.12	1.77	0	0.99	9.04	0.16	1.99	0	0	0	0	0	0	1.21	0.62	0	0	0	0	0	0	0	0	0	0	0	0	0	0	0	0	0	0	0	0	0	0	0	0	0	0	22.58
CAGCCAAGGATGACTTGC	0.79	0.3	0	1.46	0	0.32	0	0	0	0	32.75	0	0	0	0	0	0.15	0.47	0	0	0	0	0	0	0	0	0	0	0	4.07	0	0	6.92	0	0	0	0	6.05	0	0	0	0	0	0	53.28
CAGCCAAGGATGACTTGCCG	1.85	0.2	0	1.22	0	0.22	0.79	0	0	0.25	208.8	8.25	0	0	19.98	8.25	0.6	0.62	0	0	0	0	0	0	0	0	17.73	0	0	0	0	0	6.92	43.64	425.01	0	0	6.05	0	6.07	0	0	0	0	756.45
CAGCCAAGGATGACTTGCCGGCCG	0	0	0	0	0.22	0	0	0	0	0.25	0	0	0	0	0	0	0	0	0	0	0	0	0	0	0	0	0	0	0	0	0	0	0	0	0	0	0	0	0	0	0	0	0	0	0.47
AGCCAAGGATGACTTGCCGG	20.32	1	2.04	25.38	9.52	0.22	3.95	0	0	1.24	843.37	8.25	0	0	19.98	8.25	2.11	1.61	0	0	0	0	0	0	0	0	0	0	0	0	0	0	0	0	91.07	0	0	6.05	0.31	0	0	0	0	0	1044.47
AGCCAAGGATGACTTGCCGGC	5.01	0.6	8.96	10.74	31.66	0	7.31	18.07	0	3.97	0	24.75	28.16	28.16	19.98	24.75	0.15	0.16	0	0	0.22	0	0	0	0	0	8.87	0	0	0	0	29.32	0	0	0	0	0	0	0.31	0	0	0	0	0	251.15
AGCCAAGGATGACTTGC	0	0	0	0	0	0	0	0	0	0	0	0	0	0	0	0	0	0	0	0	0	0	0	0	0	0	0	0	0	0	0	0	0	0	10.12	0	0	6.05	0	0	0	0	0	0	16.17
AGCCAAGGATGACTTGCCG	0	0.1	0	0.24	0	0	0	0	0	0	65.5	8.25	0	0	19.98	8.25	0.15	0	0	0	0	0	0	0	0	0	0	0	0	0	0	0	0	0	0	0	0	6.05	0	0	0	0	0	0	108.52
AGCCAAGGATGACTTGCC	0	0	0	0	0	0	0	0	0	0	8.19	0	0	0	0	0	0	0	0	0	0	0	0	0	0	0	0	0	0	0	0	0	0	0	0	0	0	0	0	0	0	0	0	0	8.19
GCCAAGGATGACTTGCCG	0	0	0	0	0	0	0	0	0	0	12.28	0	0	0	0	0	0	0	0	0	0	0	0	0	0	0	0	0	0	0	0	0	0	0	0	0	0	0	0	0	0	0	0	0	12.28
GCCAAGGATGACTTGCCGG	0	0	0.41	0	47.15	0	3.55	0	0	2.23	712.36	0	0	0	0	0	0	0.08	0	0	0.22	0	26	0	0	0	0	0	0	0	0	0	0	0	0	0	0	0	0	0	0	0	0	0	792
GCCAAGGATGACTTGCC	0	0	0	0	0	0	0	0	0	0	0	0	0	0	0	0	0	0	0	0	0	0	0	0	0	0	0	0	0	0	0	0	0	0	0	0	0	6.05	0	0	0	0	0	0	6.05
GCCAAGGATGACTTGCCGGC	0	0.1	0.2	0.24	0	0	0.2	0	0	0	0	0	0	0	0	0	0	0	0	0	0	0	0	0	0	0	0	0	0	0	0	0	0	0	0	0	0	0	0	0	0	0	0	0	0.74
CCAAGGATGACTTGCCGG	0	0	0	0	0	0	0	0	0	0	188.33	0	0	0	0	0	0	0	0	0	0	0	0	0	0	0	0	0	0	0	0	0	0	0	0	0	0	0	0	0	0	0	0	0	188.33
CAAGGATGACTTGCCGG	0	0	0	0	0	0	0	0	0	0	0	0	0	0	0	0	0	0	0	0	0	0	0	0	0	0	0	0	0	0	0	0	0	0	10.12	0	0	6.05	0	0	0	0	0	0	16.17
CGGCAAGTTTGTCCTTGGCTAC	1.06	1	0.2	2.93	0	0.22	0	54.22	0	0	0	41.26	225.31	225.31	0	41.26	0	0	0	0	0	0	0	0	28.07	0	0	0	0.33	0	11.76	0	0	0	0	0	167.89	0	0	0	0	61.45	16.03	0	878.3
CGGCAAGTTTGTCCTTGGCTA	0	0	0	0	0.22	0	0.39	0	0	0	0	0	0	0	0	0	0	0	0	0	0	0	0	0	0	0	0	0	0	0	0	0	0	0	0	0	0	0	0	0	0	0	0	0	0.61
CGGCAAGTTTGTCCTTGGCT	0	0	0	0.24	0	0	0	0	0	0	0	0	0	0	0	0	0	0	0	0	0	0	0	0	0	0	0	0	0	0	0	0	0	0	0	0	0	0	0	0	0	0	0	0	0.24
GGCAAGTTTGTCCTTGGCTACA	0.53	1	0.2	0.98	0	0.11	0.39	0	0	0	0	24.75	28.16	28.16	0	24.75	0	0	0	0	0	0	0	0	0	0	8.87	0	0.33	0	23.53	0	0	0	0	0	15.26	0	0	0	0	0	0	0	157.02
GGCAAGTTTGTCCTTGGCTAC	1.32	1	0	2.2	0	0.11	0.99	18.07	0	0	0	0	28.16	28.16	0	0	0	0	19.37	0	0	0	0	0	14.04	0	0	0	0	0	11.76	7.33	0	0	0	0	45.79	0	0	0	0	0	0	0	178.3
GGCAAGTTTGTCCTTGGCTA	0	0	1.02	0.24	0.22	0	0.79	0	0	0.25	0	8.25	0	0	0	8.25	0	0	0	0	0	0	0	0	0	0	0	0	0	0	0	0	0	0	0	0	0	0	0	0	0	0	0	0	19.02
GGCAAGTTTGTCCTTGGCT	0	0	0	0	0	0	0	0	0	0.25	0	0	0	0	0	0	0	0	0	0	0	0	0	0	0	0	0	0	0	0	0	0	0	0	0	0	0	0	0	0	0	0	0	0	0.25
GGCAAGTTTGTCCTTGGC	0.26	0	0	0	0	0	0	0	0	0	0	0	0	0	0	0	0	0	0	0	0	0	0	0	0	0	0	0	0	0	0	0	0	0	0	0	0	0	0	0	0	0	0	0	0.26
GCAAGTTTGTCCTTGGCTAC	0	0	0	0.24	0	0	0	0	0	0	0	0	0	0	0	0	0	0	0	0	0	0	0	0	0	0	0	0	0	0	0	0	0	0	0	0	0	0	0	0	0	0	0	0	0.24
CAAGTTTGTCCTTGGCTACACCTT	0.26	0	0	0	0	0	0	0	0	0	0	0	0	0	0	0	0	0	0	0	0	0	0	0	0	0	0	0	0	0	0	0	0	0	0	0	0	0	0	0	0	0	0	0	0.26
AAGTTTGTCCTTGGCTACACCTTGC	0	0	0	0.24	0	0	0	0	0	0	0	0	0	0	0	0	0	0	0	0	0	0	0	0	0	0	0	0	0	0	0	0	0	0	0	0	0	0	0	0	0	0	0	0.37	0.61
AAGTTTGTCCTTGGCTACACCTTG	0.26	0.1	0	0.49	0	0	0	0	1.32	0	0	0	0	0	0	0	0	0	0	0	0	0	0	0	0	0	0	0	0	0	0	0	0	0	0	0	0	0	0	0	0	0	0	0.73	2.9
AAGTTTGTCCTTGGCTACACCTT	0	0	0	0.24	0	0	0	0	0	0	0	0	0	0	0	0	0	0	0	0	0	0	0	0	0	0	0	0	0	0	0	0	0	0	0	0	0	0	0	0	0	0	0	0	0.24
AGTTTGTCCTTGGCTACACCTTGC	0	0	0	0	0	0	0	0	0.16	0	0	0	0	0	0	0	0	0	0	0	0	0	0	0	0	0	0	0	0	0	0	0	0	0	0	0	0	0	0	0	0	0	0	0	0.16
TTTGTCCTTGGCTACACCTTGC	0	0	0	0	0	0	0	0	0.33	0	0	0	0	0	0	0	0	0	0	0	0	0	0	0	0	0	0	0	0	0	0	0	0	0	0	0	0	0	0	0	0	0	0	0	0.33
TTTGTCCTTGGCTACACCTTG	0	0	0	0	0.22	0	0	0	0	0	0	0	0	0	0	0	0	0	0	0	0	0	0	0	0	0	0	0	0	0	0	0	0	0	0	0	0	0	0	0	0	0	0	0	0.22
TTGTCCTTGGCTACACCTTGC	0	0	0	0.24	0	0	0	0	0	0	0	0	0	0	0	0	0	0	0	0	0	0	0	0	0	0	0	0	0	0	0	0	0	0	0	0	0	0	0	0	0	0	0	0	0.24
TGTCCTTGGCTACACCTTGCT	0	0	0.2	0	0	0	0.2	0	0	0	0	0	0	0	0	0	0	0	0	0	0	0	0	0	0	0	0	0	0	0	0	0	0	0	0	0	0	0	0	0	0	0	0	0	0.4
GTCCTTGGCTACACCTTGCT	0	0	0	0.24	0	0	0	0	0	0	0	0	0	0	0	0	0	0	0	0	0	0	0	0	0	0	0	0	0	0	0	0	0	0	0	0	0	0	0	0	0	0	0	0	0.24

GAGCAGGATGCAGCCAAGGATGACTTGCCGGCCGGCGATGGCCGACGGCGAGGTTAATTAATTGGCCGGAGACTGGCAGTCCTTCTCTGTTGATCCGGCAAGTTTGTCCTTGGCTACACCTTGCTC

GAGCAGGATGCAGCCAAGGATGACTTGCCGGCCGGCGATGGCCGACGGCGAGGTTAATTAATTGGCCGGAGACTGGCAGTCCTTCTCTGTTGATCCGGCAAGTTTGTCCTTGGCTACACCTTGCTC

-60.51 (((((((.((.((((((((((((((((((((.(((((((.((((.((((..((((....)))).)))).....)))).)))........))))..))))))))))).))))))))))).)).))))))) []

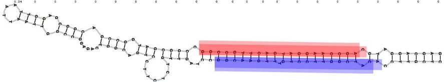

RC_osa-MIR169p

	GSM520635	GSM309692	GSM407071	GSM329297	GSM520638	GSM309693	GSM407072	GSM409324	Run1	Run2	GSM409321	GSM520640	GSM409322	GSM309691	GSM520637	GSM455965	GSM520639	GSM409323	GSM409320	GSM455963	GSM455962	GSM686040	GSM693279	GSM686039	GSM693280	GSM571077	GSM571078	GSM409319	GSM329296	GSM329298	GSM329299	GSM409315	GSM409318	GSM409314	GSM409317	GSM409316	GSM278535	GSM409313	GSM278572	Total
GATAGCCAAGGATGACTTGCCTGTGT	0.16	0	0	0	0	0	0	0	0	0	0	0	0	0	0	0	0	0	0	0	0	0	0	0	0	0	0	0	0	0	0	0	0	0	0	0	0	0	0	0.16
ATAGCCAAGGATGACTTGCC	0	19.98	0	0	0	6.05	0	0	0	0	0	0	0	40.48	0	0	0	0	0	0	0	0	0	0	0	0	0	0	0	0	0	0	0	0	0	0	0	0	0	66.51
ATAGCCAAGGATGACTTGCCTGT	0	0	28.16	0	0	0	0	0	28.16	0	0	0	0	0	0	0	0	0	0	0	0	0	0	0	0	0	0	0	0	0	0	0	0	0	0	0	0	0	0	56.32
ATAGCCAAGGATGACTTG	0	0	0	49.13	0	0	0	0	0	0	0	0	0	0	0	0	0	0	0	0	0	0	0	0	0	0	0	0	0	0	0	0	0	0	0	0	0	0	0	49.13
ATAGCCAAGGATGACTTGC	0	19.98	0	0	0	6.05	0	0	0	0	0	0	0	0	0	0	0	0	0	0	0	0	0	0	0	0	0	0	0	0	0	0	0	0	0	0	0	0	0	26.03
ATAGCCAAGGATGACTTGCCTG	0	0	619.6	0	0.41	0	66.01	45.19	619.6	66.01	14.66	3.16	35.29	0	0	0	0	0	0	0	0	0	0	0	0	0	0	0	0	0	0	0	0	0	0	0	0	0	0	1469.93
ATAGCCAAGGATGACTTGCCT	0	0	56.33	0	0	0	0	0	56.33	0	0	0	0	0	0	0	0	0	0	0	0	0	0	0	0	0	0	0	0	0	0	0	0	0	0	0	0	0	0	112.66
TAGCCAAGGATGACTTGCCTGT	0	0	1915.12	0	5.7	0	33	72.3	1915.12	33	21.99	20.93	35.29	0	0.44	0.5	6.2	198.41	12.29	0	0	0.15	0	0	0	1.06	0.49	0	0	0	0	8.87	8.76	48.09	0	0	0	0	0	4337.71
TAGCCAAGGATGACTTGCCTGTG	0	0	112.65	0	0.81	0	8.25	0	112.65	8.25	0	3.55	0	0	0	0.74	0.44	15.26	0	0	0	0.08	0	0	0	0	0.98	0	0	0	0	8.87	0	80.15	0	0	0	10.05	0	362.73
TAGCCAAGGATGACTTGCCT	0	339.7	140.82	802.43	15.27	84.76	107.26	72.3	140.82	107.26	0	17.18	11.76	1932.77	0.22	2.48	15.72	0	0	81.26	17.94	0	0	0	0	0.53	1.46	0	0	0	0	17.73	0	0	0	0	0	0	0	3909.67
TAGCCAAGGATGACTTGCCTG	0	0	24586.7	144974.35	57.61	0	5924.29	8874.43	24586.7	5924.29	1627.49	209.54	2387.81	0	0	15.14	48.92	213.67	49.16	0.81	7.39	9.49	1.4	4.84	0.43	44.07	81.5	41.13	109.97	110.71	130.92	1329.76	201.55	288.55	154.39	91.29	623.63	251.34	0	222963.27
TAGCCAAGGATGACTTGCC	0	19.98	0	7705	1.63	6.05	0	0	0	0	0	2.76	0	0	0	0	0.66	0	0	0	0	0.3	0.1	0.08	0	1.85	0.98	0	4.07	0	0	8.87	0	0	0	0	0	0	0	7752.33
TAGCCAAGGATGACTTGC	0	0	0	61.41	0	0	0	0	0	0	0	0	0	0	0	0	0	0	0	0	0	0.15	0.1	0	0	0.26	0.49	0	0	0	0	0	0	0	0	0	89.09	0	0	151.5
TAGCCAAGGATGACTTG	0	19.98	0	0	0	0	0	0	0	0	0	0	0	0	0	0	0	0	0	0	0	0	0	0	0	0	0	0	0	0	0	0	0	0	0	0	0	0	0	19.98
AGCCAAGGATGACTTGCCTG	0	0	0	1056.26	1.02	0	33	9.04	0	33	0	1.58	0	10.12	0	0.25	0	0	0	0	0	0.15	0.1	0.08	0	3.17	4.15	0	0	0	0	0	0	0	0	0	0	0	0	1151.92
AGCCAAGGATGACTTGCCTGT	0	19.98	0	0	0	6.05	0	0	0	0	0	0	0	70.83	0	0	0	0	0	0	0	0	0	0	0	0	0.24	0	0	0	0	0	0	0	0	0	0	0	0	97.1
AGCCAAGGATGACTTGCCTGTGT	0	0	0	0	0	6.05	0	0	0	0	0	0	0	10.12	0	0	0	0	0	0	0	0	0	0	0	0	0	0	0	0	0	0	0	0	0	0	0	0	0	16.17
AGCCAAGGATGACTTGCCT	0	19.98	0	4.09	0.61	6.05	8.25	0	0	8.25	0	0	0	0	0	0	0	0	0	0	0	0	0	0	0	0	0	0	0	0	0	0	0	0	0	0	0	0	0	47.23
AGCCAAGGATGACTTGC	0	0	0	0	0	6.05	0	0	0	0	0	0	0	10.12	0	0	0	0	0	0	0	0	0	0	0	0	0	0	0	0	0	0	0	0	0	0	0	0	0	16.17
AGCCAAGGATGACTTGCCTGTGTCC	0	0	0	0	0	0	0	0	0	0	0	0	0	0	0	0	0	0	0	0	0	0	0	0	0	0	0	0	0	0	0	0	0	16.03	0	0	0	10.05	0	26.08
AGCCAAGGATGACTTGCC	0	0	0	8.19	0	0	0	0	0	0	0	0	0	0	0	0	0	0	0	0	0	0	0	0	0	0	0	0	0	0	0	0	0	0	0	0	0	0	0	8.19
GCCAAGGATGACTTGCCTG	0	0	28.16	3467.66	0	0	8.25	0	28.16	8.25	0	0.39	0	0	0	0	0	0	0	0	0	0.23	0	0	0	0.26	1.22	0	4.07	0	0	0	0	0	0	0	0	0	0	3546.65
GCCAAGGATGACTTGCC	0	0	0	0	0	6.05	0	0	0	0	0	0	0	0	0	0	0	0	0	0	0	0	0	0	0	0	0	0	0	0	0	0	0	0	0	0	0	0	0	6.05
GCCAAGGATGACTTGCCT	0	0	0	40.94	0	0	0	0	0	0	0	0	0	0	0	0	0	0	0	0	0	0	0	0	0	0	0	0	0	0	0	0	0	0	0	0	0	0	0	40.94
CCAAGGATGACTTGCCTGT	0	0	0	0	0	0	0	0	0	0	0	0	0	10.12	0	0	0	0	0	0	0	0	0	0	0	0	0	0	0	0	0	0	0	0	0	0	0	0	0	10.12
CCAAGGATGACTTGCCTG	0	0	0	3770.62	0	0	0	0	0	0	0	0	0	0	0	0	0	0	0	0	0	0	0	0	0	0	0	0	4.07	0	0	0	0	0	0	0	0	0	0	3774.69
CAAGGATGACTTGCCTGTG	0	0	0	0	0	0	0	0	0	0	0	0	0	0	0	0.25	0	0	0	0	0	0	0	0	0	0	0	0	0	0	0	0	0	0	0	0	0	0	0	0.25
CAAGGATGACTTGCCTGT	0	0	0	4.09	0	0	0	0	0	0	0	0	0	0	0	0	0	0	0	0	0	0	0	0	0	0	0	0	0	0	0	0	0	0	0	0	0	0	0	4.09
AGGATGACTTGCCTGTGT	0	0	0	0	0	0	0	0	0	0	0	0	0	0	0	0	0	0	0	0	0	0	0	0	0	0	0.24	0	0	0	0	0	0	0	0	0	0	0	0	0.24
TGGGCAGTCTCCTTGGCTAGC	0	0	0	0	0	0	16.5	0	0	16.5	0	0.2	0	0	0	0	0	0	0	0	0	0	0	0	0	0.26	0	0	0	0	0	0	0	0	0	0	0	0	0	33.46
TGGGCAGTCTCCTTGGCTAGCC	0	0	0	0	0	0	0	0	0	0	7.33	0	0	0	0	0	0	0	0	0	0	0	0	0	0	0	0	0	0	0	0	0	0	0	0	0	0	0	0	7.33
GGCAGTCTCCTTGGCTAGC	0	0	366.12	176.04	1.83	0	0	0	366.12	0	0	8.1	0	0	0	1.49	1.99	0	0	0	0	1.21	2.29	0.23	0.22	7.13	9.03	0	0	0	0	0	0	0	0	0	0	0	0.33	942.13
GGCAGTCTCCTTGGCTAG	0	0	28.16	57.32	0	0	0	0	28.16	0	0	0	0	0	0	0	0	0	0	0	0	0.53	0.2	0.08	0	0	0.98	0	0	0	0	0	0	0	0	0	0	0	0	115.43
GGCAGTCTCCTTGGCTAGCC	0	0	816.74	32.75	0.81	0	24.75	9.04	816.74	24.75	7.33	3.36	0	0	0	0.74	0	0	0	0	0	0	0	0	0	0.53	0.24	0	0	0	0	0	0	0	0	0	0	0	0	1737.78
GGCAGTCTCCTTGGCTAGCCT	0	0	0	8.19	0	0	0	0	0	0	0	0	0	0	0	0	0	0	0	0	0	0	0	0	0	0	0	0	0	0	0	0	0	0	0	0	0	0	0	8.19
GCAGTCTCCTTGGCTAGC	0	0	0	4.09	0	0	0	0	0	0	0	0	0	0	0	0	0	0	0	0	0	0	0	0	0	0.26	1.71	0	0	0	0	0	0	0	0	0	0	0	0	6.06
CAGTCTCCTTGGCTAGCCTGA	0	0	0	0	0	0	0	0	0	0	0	0.2	0	0	0	0	0.66	0	0	0	0	0	0	0	0	0	0	0	0	0	0	0	0	0	0	0	0	0	0	0.86
CAGTCTCCTTGGCTAGCC	0	0	0	4.09	0	0	0	0	0	0	0	0	0	0	0	0	0	0	0	0	0	0	0	0	0	0	0	0	0	0	0	0	0	0	0	0	0	0	0	4.09
AGTCTCCTTGGCTAGCCTG	0	0	0	4.09	0	0	0	0	0	0	0	0	0	0	0	0	0	0	0	0	0	0	0	0	0	0	0	0	0	0	0	0	0	0	0	0	0	0	0	4.09

TTATCTCTGATAGCCAAGGATGACTTGCCTGTGTCCTCCCTGAAGGATTAGCAATTTAATGATCCTTTAAGCTGGTTCATGGGCAGTCTCCTTGGCTAGCCTGAGTGG
TTATCTCTGATAGCCAAGGATGACTTGCCTGTGTCCTCCCTGAAGGATTAGCAATTTAATGATCCTTTAAGCTGGTTCATGGGCAGTCTCCTTGGCTAGCCTGAGTGG
TTATCTCTGATAGCCAAGGATGACTTGCCTGTGTCCTCCCTGAAGGATTAGCAATTTAATGATCCTTTAAGCTGGTTCATGGGCAGTCTCCTTGGCTAGCCTGAGTGG

-48.70 ....(((...((((((((((.(((.((((((((.((....((((((((((.........))))))))))....))..))))))))))))))))))))....)))... []

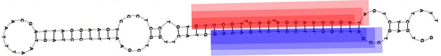

RC_osa-MIR169q

	GSM686039	GSM278572	GSM309691	GSM309692	GSM309693	Total
CCGGATCTGCCGGCGAGGAGGC	0.08	0	0	0	0	0.08
GGATCTGCCGGCGAGGAGGCC	0	0.33	0	0	0	0.33
CGGCGAGGAGGCCGGAGTCACCGGAT	0	0	10.12	0	0	10.12
GGCGAGGAGGCCGGAGTCACCGGAT	0	0	0	19.98	0	19.98
GGCGAGGAGGCCGGAGTCACCGG	0	0	10.12	0	6.05	16.17
GGCGAGGAGGCCGGAGTCACC	0	0	0	19.98	6.05	26.03
GGCGAGGAGGCCGGAGTCACCGGA	0	0	0	0	6.05	6.05
GCGAGGAGGCCGGAGTCACCGG	0	0	0	19.98	0	19.98
CGAGGAGGCCGGAGTCACC	0	0	0	19.98	0	19.98
CGAGGAGGCCGGAGTCACCGG	0	0	0	19.98	0	19.98
CGAGGAGGCCGGAGTCA	0	0	0	0	6.05	6.05
CGAGGAGGCCGGAGTCAC	0	0	0	0	6.05	6.05
ATCCGGTGGTCCCGGCCTCC	0	0	20.24	0	0	20.24
ATCCGGTGGTCCCGGCCTCCTCGTT	0	0	70.83	19.98	6.05	96.86
ATCCGGTGGTCCCGGCCTCCTCGT	0	0	10.12	0	6.05	16.17
CCGGTGGTCCCGGCCTCCTCGT	0	0	0	0	6.05	6.05
CCGGTGGTCCCGGCCTCCTCG	0	0	10.12	0	6.05	16.17
CCGGTGGTCCCGGCCTCCTCGTT	0	0	91.07	19.98	6.05	117.1
CCGGTGGTCCCGGCCTCC	0	0	10.12	19.98	6.05	36.15
CCGGTGGTCCCGGCCTCCTC	0	0	0	19.98	0	19.98
CGGTGGTCCCGGCCTCCTCGTT	0	0	0	19.98	6.05	26.03
GGTGGTCCCGGCCTCCTCGTT	0	0	10.12	19.98	0	30.1
GTGGTCCCGGCCTCCTCGTT	0	0	20.24	0	6.05	26.29
GGTCCCGGCCTCCTCGTT	0	0	0	0	6.05	6.05

CCGGATCTGCCGGCGAGGAGGCCGGAGTCACCGGATCCGGTGGTCCCGGCCTCCTCGTTGGCGGATGCG

-61.60 .((.((((((((((((((((((..((((((....))))))..)))))))))))))))))).)) []

RC_osa-MIR1846e

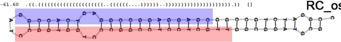

Sequence	GSM520640	GSM455965	GSM520638	GSM520639	Run2	GSM693279	GSM693280	GSM686040	GSM571077	GSM571078	GSM686039	GSM409315	GSM278572	GSM409322	GSM407072	GSM329296	GSM329297	GSM329298	GSM409323	GSM520637	GSM520636	GSM455963	Total
TTGGAGCATGAGGTTATCTCT	0.59	0.74	0.61	0	0	0	0	0	0	0	0	0	0	0	0	0	0	0	0	0	0	0	1.94
TGGAGCATGAGGTTATCTCT	0.2	0	0.41	0.44	0	0	0	0	0	0	0	0	0	0	0	0	0	0	0	0	0	0	1.05
TGGAGCATGAGGTTATCTCTC	5.13	3.47	3.26	5.98	8.25	1.99	0.86	0.23	0.79	1.46	0.55	8.87	0.67	11.76	8.25	8.15	20.47	6.92	15.26	0.22	0.7	95.88	209.12
TGGAGCATGAGGTTATCTC	0	0	0	0	0	0.2	0	0	0	0	0	0	0	0	0	0	0	0	0	0	0	0	0.2
GGAGCATGAGGTTATCTCTC	0	0	0	0	0	0.4	0	0.15	0	0	0.16	0	0	0	0	0	0	0	0	0	0	0	0.71
AGAGATAACCTCATGCTCCAA	0.2	0	0.2	0	0	0.1	0	0	0	0	0	0	0	0	0	0	0	0	0	0	0	0	0.5

AATTTTTTATCCTGGTTTTCTTGGAGCATGAGGTTATCTCTCGGTTTTCAAGAACAGTTTTTGTCCTTTTATTTTGGACACGGAAAAACTGAGAGATAACCTCATGCTCCAAAAAAACCAGGATAAAAAC

-75.60 ...((((((((((((((.((((((((((((((((((((((((((((.........((..(((((.........)))))..))))))))))))))))))))))))))))))).))))))))))))))). []

RC_osa-MIR1857

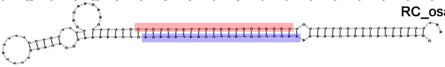

	GSM693279	GSM693280	GSM278572	GSM278571	GSM686039	GSM686040	GSM520635	GSM329298	Total
GTGCCGCAACAAGGAGAGGA	0.1	0	0	0	0	0	0	0	0.1
TGCCGCAACAAGGAGAGGAGGACG	0.1	0	0	0	0	0	0	0	0.1
CCGCAACAAGGAGAGGAGGAC	0.5	0	0	0	0	0	0	0	0.5
CCGCAACAAGGAGAGGAGGACGGA	0.1	0	0	0	0	0	0	0	0.1
CCGCAACAAGGAGAGGAGGACGG	0.1	0	0	0	0	0	0	0	0.1
CGCAACAAGGAGAGGAGGA	0.2	0	0	0	0	0	0	0	0.2
CGCAACAAGGAGAGGAGGACGG	0.1	0.11	0	0	0	0	0	0	0.21
CGCAACAAGGAGAGGAGGACG	0.1	0.11	0.33	0.31	0	0	0	0	0.85
GCAACAAGGAGAGGAGGACGGA	0.1	0	0	0	0	0	0	0	0.1
GCAACAAGGAGAGGAGGACGG	0.3	0	0	0	0	0	0	0	0.3
CAACAAGGAGAGGAGGACGGA	0.3	0	0	0	0	0	0	0	0.3
AACAAGGAGAGGAGGACGG	0.1	0	0	0	0	0	0	0	0.1
ACAAGGAGAGGAGGACGGA	0	0	0.33	0	0	0	0	0	0.33
ACAAGGAGAGGAGGACGG	0.2	0	0.33	0	0	0	0	0	0.53
GGAGGACGGATTGGGGCGAGTGGA	0	0	0	0	0.08	0	0	0	0.08
AGGACGGATTGGGGCGAGTGG	0	0	0	0	0	0.08	0	0	0.08
GGACGGATTGGGGCGAGTGGA	0.1	0	0	0	0	0	0	0	0.1
GCGAGTGGAGCATCGAAGGGG	0.1	0	0.33	0	0	0	0	0	0.43
GCGAGTGGAGCATCGAAGGG	0.1	0	0	0	0	0	0	0	0.1
GCGAGTGGAGCATCGAAGGGGATG	0	0	0	0	0	0	0.16	0	0.16
GTGGAGCATCGAAGGGGATGT	0.1	0	0	0	0	0	0	0	0.1
GGAGCATCGAAGGGGATGTCC	0.1	0	0	0	0	0	0	0	0.1
TCCTCCTCTCCTTGTTGC	0	0	0	0	0	0	0	3.46	3.46
TCCTCCTCTCCTTGTTGCGGCACC	0.1	0	0	0	0	0	0	0	0.1
TCCTCCTCTCCTTGTTGCGGC	0	0	0.33	0	0	0	0	0	0.33

GAGGCCGTCGTCGCTGCCAGCGAAGGGGGGTGCCGCAACAAGGAGAGGAGGACGGATTGGGGCGAGTGGAGCATCGAAGGGGATGTCCCACGAGCAGATTCGGCGGCGATGACATCCCCTTTGACGCTCCACTCGCCCCACTCCGTCCTCCTCTCCTTGTTGCGGCACCCCCCCTCCCTTTGCCGGCAGCGATGACGGGA

-177.70 ....((((((((((((.((((((((((((((((((((((((((((((((((((.((((((((((((((.((((((((((((((.((((....))))..))....))))))))))))).))))))))))))))).)))))))))))))))))))))))))))))))))).....))))).)))))))))))))).. []

RC_osa-MIR1858a

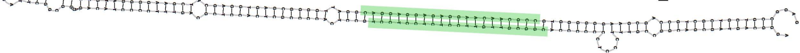

Sequence	GSM686039	GSM520634	GSM686040	GSM571077	GSM693279	GSM520638	GSM693280	Run2	GSM407072	GSM520637	GSM571078	GSM455965	GSM520635	Total
GATACTCCCTCCGTCCCAAAAT	0.08	0	0	0	0	0	0	0	0	0	0	0	0	0.08
ATACTCCCTCCGTCCCAAAATAAA	0.08	0	0	0	0	0	0	0	0	0	0	0	0	0.08
ACTCCCTCCGTCCCAAAATA	0	0.37	0	0	0	0	0	0	0	0	0	0	0	0.37
ACTAGCTTTGTTTATTTTG	0.08	0	0.15	0.26	0	0	0	0	0	0	0	0	0	0.49
ACTAGCTTTGTTTATTTTGG	0	0	0.08	0	0	0	0	0	0	0	0	0	0	0.08
ACTAGCTTTGTTTATTTT	0	0	0	0	0.1	0	0	0	0	0	0	0	0	0.1
CTAGCTTTGTTTATTTTGGG	0	0	0	0	0	0.2	0	0	0	0	0	0	0	0.2
TGTTTATTTTGGGACAGAGGG	0.08	0	0	0	0	0.2	0.11	0	0	0	0	0	0	0.39
GTTTATTTTGGGACAGAGGGAGT	0	0	0	0	0	0	0	8.25	8.25	0	0	0	0	16.5
GTTTATTTTGGGACAGAGGGAGTA	0	0	0	0	0	0	0	0	0	0.22	0	0	0	0.22
TTATTTTGGGACAGAGGGAGTA	0	0	0	0	0	0.2	0	0	0	0	0	0	0	0.2
TTATTTTGGGACAGAGGGAG	0	0	0.08	0	0	0	0	0	0	0	0	0	0	0.08
TATTTTGGGACAGAGGGAGTA	0	0	0	0.26	0.1	0	0.11	0	0	0	0.49	0	0	0.96
TATTTTGGGACAGAGGGAG	0	0	0	0	0	0	0.11	0	0	0	0	0	0	0.11
TATTTTGGGACAGAGGGA	0	0	0	0.26	0	0	0	0	0	0	0	0	0	0.26
ATTTTGGGACAGAGGGAGTA	0	0	0	0	0.3	0.2	0.22	0	0	0	0	0.25	0	0.97
ATTTTGGGACAGAGGGAGT	0	0	0	0	0	0.41	0	0	0	0	0	0	0	0.41
TTTGGGACAGAGGGAGTA	0.08	0	0	0	0	0	0	0	0	0	0	0	0	0.08
TTTGGGACAGAGGGAGTACCTAGC	0	0	0	0	0	0	0.11	0	0	0	0	0	0	0.11
TTTGGGACAGAGGGAGTAC	0	0	0	0	0	0	0	0	0	0	0	0	0.16	0.16

```
       TAGTAACCAGAGGAGTAACAAACTATGATACTCCCTCCGTCCCAAAATAAACAAACCTAGTACAGGACTAGGAAGTGCCACATCCCCGTACTAGCTTTGTTTATTTTGGGACAGAGGGAGTACCTAGCAAGCTACGAGACTAGCCAGTAG
       TAGTAACCAGAGGAGTAACAAACTATGATACTCCCTCCGTCCCAAAATAAACAAACCTAGTACAGGACTAGGAAGTGCCACATCCCCGTACTAGCTTTGTTTATTTTGGGACAGAGGGAGTACCTAGCAAGCTACGAGACTAGCCAGTAG
-57.46 (((......(((...............((((((((.((((((((((((((((.(((((((.(((...((.....))...)))..))))))).))))))))))))))))).)))))))))).....))))...............  []
```

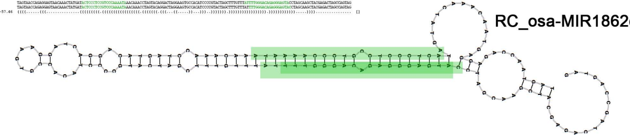

RC_osa-MIR1862d

Sequence	GSM693279	GSM686040	GSM520639	GSM571077	GSM686039	GSM571078	GSM693280	GSM278571	GSM278572	GSM520640	GSM520636	GSM520635	GSM409320	Total
AGGAAGGAGTATCTAAGGACA	0.2	0	0	0	0	0	0	0	0	0	0	0	0	0.2
GGAGTATCTAAGGACAATA	0	0.08	0	0	0	0	0	0	0	0	0	0	0	0.08
AGTATCTAAGGACAATAGGAA	0	0	0.44	0	0	0	0	0	0	0	0	0	0	0.44
CTAAGGACAATAGGAATGGGAGGC	0	0	0	0.26	0	0	0	0	0	0	0	0	0	0.26
CTAAGGACAATAGGAATGGGAGGCA	0	0	0	0	0.08	0	0	0	0	0	0	0	0	0.08
TAAGGACAATAGGAATGGGAGGCAC	1.49	0.6	0	0.26	1.64	0.49	0.22	0.61	0.33	0	0	0	0	5.64
TAAGGACAATAGGAATGGGAGGCA	1.1	2.19	0	0	3.2	0	0.22	0	0	0	0	0	0	6.71
TAAGGACAATAGGAATGGGAG	0	0.08	0	0	0.08	0	0	0	0	0	0	0	0	0.16
TAAGGACAATAGGAATGGGAGGC	0.5	0.9	0	0.26	0.7	0	0	0.31	0.33	0	0	0	0	3
TAAGGACAATAGGAATGGGAGG	0	0.08	0	0	0.08	0	0	0	0	0	0	0	0	0.16
TAAGGACAATAGGAATGGGA	0	0.08	0	0	0	0	0	0	0	0	0	0	0	0.08
AAGGACAATAGGAATGGGAGGCAC	0.5	0.38	0.66	0	0.39	0.24	0.11	0.31	0.33	0	2.1	0	0	5.02
AAGGACAATAGGAATGGGAGGCACT	1.59	0	0	0.26	0.23	0.73	0.32	0.31	0.33	0.39	2.1	0	0	6.26
AAGGACAATAGGAATGGGAGGCA	0.1	0.23	0	0.26	0.47	0.49	0	0	0	0	0	0	0	1.55
AAGGACAATAGGAATGGGAGGCACTA	0.1	0	0	0	0	0	0	0	0	0	0	0	0	0.1
AAGGACAATAGGAATGGGAGGC	0	0.23	0	0	0.31	0	0	0	0	0	0	0	0	0.54
AGGACAATAGGAATGGGAGGCA	0.1	0	0	0	0	0	0	0	0	0	0	0	0	0.1
GGACAATAGGAATGGGAGGC	0.1	0	0	0	0	0	0	0	0	0	0	0	0	0.1
GTTCCCATTCCGATTGCCCTTAGA	0	0	0	0	0	0	0	0	0	0	0	0.16	0	0.16
ATTCCGATTGCCCTTAGACAC	0	0	0	0	0	0	0	0	0	0	0	0	12.29	12.29

```
      GCAAGCTGAAAGGAAGAGGAAGGAGTATCTAAGGACAATAGGAATGGGAGGCACTAGGAAAACACAAAAACATTATCTTTACCCCCCACTTCTAGTGGACGAGAAAGACGCTGTAGGCTTTCCTCAATGTTCCCATTCCGATTGCCCTTAGACACTCAATTTCTTCCTCTTCCTTACTTGC
-73.90 (((((....((((((((((((((((.(((((((.((((.(((((((((.(((..((((((........(((...(((((.....((((.....)))).....)))))...)))....))))))...))))))))))))).)))).))))))).)))).....)))))))))))))).)))))   []
```

RC_osa-MIR2118i

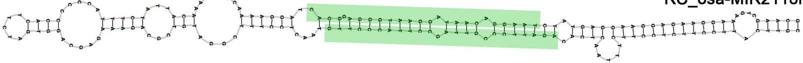

Sequence	GSM520639	GSM693279	GSM686039	GSM571077	GSM693280	GSM571078	GSM686040	GSM278572	GSM278571	GSM520635	GSM520634	GSM455965	GSM520640	GSM520636	GSM520637	Total
AAGAGGAAGGAGTACCTAAGA	0.22	0	0	0	0	0	0	0	0	0	0	0	0	0	0	0.22
ACCTAAGATCGGTAGGAATAGGAG	0	0.1	0	0	0	0	0	0	0	0	0	0	0	0	0	0.1
CCTAAGATCGGTAGGAATAGGAGGC	0	0	0.08	0.26	0	0	0	0	0	0	0	0	0	0	0	0.34
TAAGATCGGTAGGAATAGGAGGCA	0	0.8	0.94	0	0.11	0	0.83	0.67	0	0	0	0	0	0	0	3.35
TAAGATCGGTAGGAATAGGAGGCAT	0	0.9	3.83	0	0.65	0.24	1.51	1	0.61	0.16	0	0	0	0	0	8.9
TAAGATCGGTAGGAATAGGAGGC	0	0.1	0	0	0	0	0	0	0	0	0	0	0	0	0	0.1
TAAGATCGGTAGGAATAGGA	0	0	0	0	0	0	0	0	0.31	0	0	0	0	0	0	0.31
AAGATCGGTAGGAATAGGAGGCATC	0	0.7	5.39	0.79	0.11	0.73	6.1	1	0	2.8	1.47	0.74	0.39	61.02	0.22	81.46
AAGATCGGTAGGAATAGGAGGCAT	0	0.2	2.11	0.26	0	0.24	2.56	0.33	0	0	0	0	0	0.7	0	6.4
AAGATCGGTAGGAATAGGAGGCA	0	0	0.31	0	0	0	0.45	0	0	0	0	0	0	0	0	0.76
AAGATCGGTAGGAATAGGAGGC	0	0	0.08	0	0	0	0.08	0	0	0	0	0	0	0.23	0	0.39
AGATCGGTAGGAATAGGAGGCATC	0	6.38	2.65	0	0.75	0.24	4.67	0	0	0	0	0	0	0.47	0	15.16
AGATCGGTAGGAATAGGAGGCA	0	0.4	0.16	0	0	0	0.6	0	0	0	0	0	0	0	0	1.16
AGATCGGTAGGAATAGGAGGC	0	0	0.08	0	0	0	0.08	0	0	0	0	0	0	0	0	0.16
AGATCGGTAGGAATAGGAGGCAT	0	1.2	1.25	0	0.22	0.24	1.21	0	0	0	0	0	0	0	0	4.12
ATCGGTAGGAATAGGAGGCA	0	0.1	0	0	0	0	0	0	0	0	0	0	0	0	0	0.1
TCGGTAGGAATAGGAGGCATC	0	0.1	0	0	0	0	0	0	0	0	0	0	0	0	0	0.1
GGTAGGAATAGGAGGCATCGGGAAA	0	0	0	0	0	0	0	0.33	0	0	0	0	0	0	0	0.33
GGTAGGAATAGGAGGCATC	0	0.1	0.31	0	0.11	0	0.38	0	0	0	0	0	0	0.47	0	1.37
GGTAGGAATAGGAGGCAT	0	0	0.08	0	0	0	0	0	0	0	0	0	0	0	0	0.08
GGTAGGAATAGGAGGCATCGGGAA	0	0	0.08	0	0	0	0	0	0	0	0	0	0	0	0	0.08
GTAGGAATAGGAGGCATCGGG	0	0	0.08	0	0	0	0	0	0	0	0	0	0	0	0	0.08
GTAGGAATAGGAGGCATCGGGAA	0	0	0.08	0	0	0	0	0	0	0	0	0	0	0	0	0.08
GTAGGAATAGGAGGCATCGGGAAA	0	0.1	0.47	0	0	0	0.15	0.67	0	0	0	0	0	0	0	1.39
TAGGAATAGGAGGCATCGGGAAA	0	0	0.16	0	0	0	0.08	0	0	0	0	0	0	0	0	0.24
TAGGAATAGGAGGCATCGGGAAAA	0	0	0.08	0	0	0	0	0	0	0	0	0	0	0	0	0.08
TAGGAATAGGAGGCATCGGGAAAAC	0	0	0.08	0	0	0	0	0	0	0	0	0	0	0	0	0.08
AGGAATAGGAGGCATCGGGAAAAC	0	0.1	0	0	0	0	0.08	0	0	0.16	0	0	0	0	0	0.34
AGGCATCGGGAAAACGCAGAGCAA	0	0	0.08	0	0	0	0.08	0	0	0	0	0	0	0	0	0.16
TTTCCTCCATGTTCCTATTCCCA	0	0	0	0	0	0	0.08	0	0	0	0	0	0	0	0	0.08
TGTTCCTATTCCCATCGCTCTTAG	0	0	0	0	0	0	0	0	0	0.16	0	0	0	0	0	0.16
TCCTATTCCCATCGCTCTTAGAGG	0	0	0	0	0	0	0	0	0	0.16	0	0	0	0	0	0.16

ATGAGCTTAAAGGAAGAGGAAGGAGTACCTAAGATCGGTAGGAATAGGAGGCATCGGGAAAACGCAGAGCAAATTTCTAACTCCGATACTGGAGTAGAAAATTTAGCTTCAGGCTTTCCTCCATGTTCCTATTCCCATCGCTCTTAGAGGCTCTCTTTCTTCCTCTTCCCTGCTCAC

-80.30 .((((.....((((((((((((((..((((((.((((.(((((((((.((((.((((((..((.(((((((((((((.((((((....)))))))))))).))).))))...))))))))..)))))))))))))).)))).))))))..))))).....)))))))))))..))))). []

RC_osa-MIR2118q

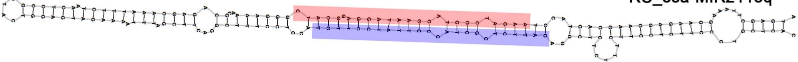

	GSM455965	GSM520635	GSM686040	GSM693279	GSM686039	GSM520637	GSM520638	GSM520640	GSM571078	GSM693280	GSM520636	GSM329298	GSM520634	Total
AGAACAACCGGGACTAAAGAT	0.25	0	0.15	0	0	0	0	0	0	0	0	0	0	0.4
AGAACAACCGGGACTAAAG	0	0.16	0	0	0	0	0	0	0	0	0	0	0	0.16
AGAACAACCGGGACTAAAGATC	0	0	0	0.1	0	0	0	0	0	0	0	0	0	0.1
GAACAACCGGGACTAAAGATC	0	0	0	0	0.08	0	0	0	0	0	0	0	0	0.08
GAACAACCGGGACTAAAGATCTTT	0	0	0	0	0	0.22	0	0	0	0	0	0	0	0.22
AACAACCGGGACTAAAGATCTT	0	0	0.23	0	0.08	0	0.2	0	0	0	0	0	0	0.51
AACAACCGGGACTAAAGATC	0	0	0	0	0.16	0	0.41	0.2	0	0	0	0	0	0.77
AACAACCGGGACTAAAGA	0	0	0.08	0	0.23	0	0	0	0.24	0.11	0	0	0	0.66
AACAACCGGGACTAAAGATCT	0	0	0	0	0	0.22	0.41	0	0	0	0.23	0	0	0.86
AACAACCGGGACTAAAGAT	0	0	0.45	0	0.08	0	0	0	0	0	0.23	0	0	0.76
ACAACCGGGACTAAAGATC	0	0	0.08	0	0	0	0.2	0	0	0	0	0	0	0.28
ACAACCGGGACTAAAGATCT	0	0	0	0	0	0.22	0.41	0.39	0	0	0	0	0	1.02
CAACCGGGACTAAAGATC	0	0	0.08	0	0	0	0	0	0	0.11	0	3.46	0	3.65
CAACCGGGACTAAAGATCT	0	0	0	0	0.08	0	0	0	0	0	0	0	0	0.08
CAACCGGGACTAAAGATCTT	0	0	0	0	0.08	0.22	0	0	0	0	0	0	0	0.3
GGGACTAAAGATCTTTTCTTTACT	0.5	0	0	0	0	0	0	0	0	0	0	0	0	0.5
ACTAAAGATCTTTTCTTTACTCCC	0.25	0	0	0	0	0	0	0	0	0	0	0	0	0.25
AAGATCTTTTCTTTACTCCCGGTT	0	0	0	0	0	0	0	0	0	0	0	0	0.37	0.37
AAGATCTTTTCTTTACTCCCGGT	0	0.16	0	0	0	0	0	0	0	0	0	0	0	0.16
GATATCAAGATATCTTTAGTCCCG	0.25	0	0	0	0	0	0	0	0	0	0	0	0	0.25
ATATCAAGATATCTTTAGTCCCGG	0	0.16	0	0	0	0	0	0	0	0	0	0	0	0.16
TATCAAGATATCTTTAGTCCCGGT	0	0	0.08	0	0	0	0	0	0	0	0	0	0	0.08
ATCAAGATATCTTTAGTCCCGGT	0	0.16	0	0	0	0	0	0	0	0	0	0	0	0.16
ATCAAGATATCTTTAGTCCCGGTT	0	0.16	0	0	0	0	0.2	0.2	0	0	0	0	0.73	1.29
AAGATATCTTTAGTCCCGGTTGTT	0	0.33	0	0	0	0	0	0	0	0	0	0	0	0.33
AGATATCTTTAGTCCCGGTTG	0	0	0.15	0	0.16	0	0	0	0	0	0	0	0	0.31
AGATATCTTTAGTCCCGGT	0	0.16	0	0	0	0	0	0	0	0	0	0	0	0.16

```
       AGAACAACCGGGACTAAAGATCTTTTCTTTACTCCCGGTTAAAGATATCAAGATATCTTTAGTCCCGGTTCTT
-34.60 ..((((((((((((((((((((.((((((.....)).)))))....))))..))))))))))))))))))) []
```

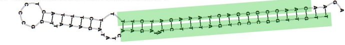

RC_osa-MIR2120

	GSM520635	GSM455965	GSM693279	GSM520637	GSM520638	GSM686039	GSM686040	GSM520640	GSM278572	GSM520636	GSM520634	Total
CGTGCCTTTGTCACTGACATGTAG	0.16	0	0	0	0	0	0	0	0	0	0	0.16
TGCCTTTGTCACTGACATGT	0	0.25	0	0	0	0	0	0	0	0	0	0.25
GCCTTTGTCACTGACATGT	0	0	0.1	0	0	0	0	0	0	0	0	0.1
ATGGGCCCACATGTCAGTGACAAA	0.16	0	0	0	0	0	0	0	0	0	0	0.16
CCACATGTCAGTGACAAAGGCACA	0	0	0	0.22	0	0	0	0	0	0	0	0.22
CACATGTCAGTGACAAAGGCAC	0	0	0.1	0	0.2	0.08	0	0	0	0	0	0.38
CACATGTCAGTGACAAAGGCA	0	0	0	0	0	0.08	0	0	0	0	0	0.08
ACATGTCAGTGACAAAGGCAC	0	0	0	0	0	0.08	0.15	0	0	0	0	0.23
ACATGTCAGTGACAAAGGCACAGT	0	0	0	0	0	0	0	0.2	0	0	0	0.2
ACATGTCAGTGACAAAGGCACA	0	0.25	0	0	0	0	0	0	0	0	0	0.25
ACATGTCAGTGACAAAGGCA	0	0	0	0	0.2	0	0	0	0	0	0	0.2
CATGTCAGTGACAAAGGCA	0	0	0	0	0	0	0.08	0	0	0	0	0.08
CATGTCAGTGACAAAGGCACAGTG	0	0	0	0.22	0	0	0	0	0	0	0	0.22
CATGTCAGTGACAAAGGCACAG	0	0	0.1	0	0	0	0	0	0	0	0	0.1
ATGTCAGTGACAAAGGCACAGTGC	0.16	0.25	0	0	0	0.08	0	0	0	0.23	0.37	1.09
ATGTCAGTGACAAAGGCACAG	0	0	0	0	0	0	0.08	0	0	0	0	0.08
ATGTCAGTGACAAAGGCAC	0	0	0	0	0	0	0	0	0.33	0	0	0.33
ATGTCAGTGACAAAGGCACAGT	0	0	0	0	0	0.08	0	0	0	0	0	0.08
TCAGTGACAAAGGCACAGTGC	0	0	0	0	0	0	0.08	0	0	0	0	0.08
CAGTGACAAAGGCACAGTGCA	0	0	0	0	0	0.08	0	0	0	0	0	0.08

```
       GACTGCATCGTGCCTTTGTCACTGACATGTAGGCCTGGAGATCAATGGGCCCACATGTCAGTGACAAAGGCACAGTGCAGT
-60.30 .(((((((.(((((((((((((((((((((.((((((........)))))).))))))))))))))))))))).))))))) []
```

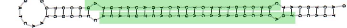

RC_osa-MIR2905

Sequence	GSM686040	GSM520638	GSM309693	GSM309691	GSM309692	Total
CAACGGGAGAAAGCACGCAGG	0.08	0	0	0	0	0.08
CCGGCGTGCTCTCTCTCGTTG	0	0.2	0	0	0	0.2
GCGTGCTCTCTCTCGTTGTCA	0	0	6.05	70.83	0	76.88
GCGTGCTCTCTCTCGTTGTC	0	0	6.05	0	0	6.05
GCGTGCTCTCTCTCGTTGT	0	0	6.05	0	0	6.05
GTGCTCTCTCTCGTTGTCA	0	0	0	10.12	19.98	30.1

GGCAGTGACAACGGGAGAAAGCACGCAGGTACGCCGCCGCGGGCCGGCTCACCGTGACCGCCGCACCGGCGTGCTCTCTCTCGTTGTCACCGCC

-59.90 (((.((((((((((((((.(((((((.(((.....((.((((..(((....)))...)))).)))))).))))))).))))))))))))))).))) []

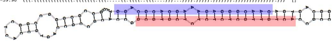

RC_osa-MIR535

Sequence	PTR1 Leaves_trimmed	PTR2 Xylem_trimmed	PTR3 Mechanically treated xylem_trimmed	Total
ATTCCACGGCTTTCTTGAACTT	0.22	0	0	0.22
TTCCACGGCTTTCTTGAAC	6.26	5.26	1.76	13.28
TTCCACGGCTTTCTTGAACTT	124.53	85.55	28.69	238.77
TTCCACGGCTTTCTTGAA	1.79	0.44	1.01	3.24
TTCCACGGCTTTCTTGAACTTC	1.57	0	0.25	1.82
TTCCACGGCTTTCTTGAACT	12.07	3.51	1.01	16.59
TTCCACGGCTTTCTTGAACTTCT	0.22	0	0	0.22
TCCACGGCTTTCTTGAACTT	0.22	0	0	0.22
CACGGCTTTCTTGAACTT	2.01	1.32	0.5	3.83
AGGAAGTTCAAGAAAGCTGTG	0.22	0	0	0.22
TTCAAGAAAGCTGTGGAAAA	1.57	0	0	1.57

```
       TCATGCTATTCCACGGCTTTCTTGAACTTCTTAAGTTATATGTATAGCTAGCAGCACCTCTAGGCTCTAGGAAGTTCAAGAAAGCTGTGGAAAAACATGGCA
-45.19 ((((...(((((((((((((((((((((((((((.........((.....)).......)))).)))))))))))))))))))))))...))))).. []
```

RC_ptc-MIR396c

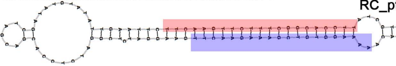

Sequence	TAE1 Leaves_trimmed	TAE2 Spikelet_trimmed	Total
TACTCCCTCCGTCTCATAATA	0.28	0	0.28
TCCCTCCGTCTCATAATATAAG	0.28	0	0.28
TTCTTATATTATGGGACGGAGGG	0.56	0	0.56
TTCTTATATTATGGGACGGAG	19.33	15.77	35.1
TCTTATATTATGGGACGGAGG	13.44	4.8	18.24
TCTTATATTATGGGACGGAGGG	0.28	0	0.28
TCTTATATTATGGGACGGAGGGAG	0.56	1.37	1.93
TCTTATATTATGGGACGGAG	1.4	1.03	2.43
TCTTATATTATGGGACGGA	0	0.34	0.34
CTTATATTATGGGACGGAGGG	6.44	0.69	7.13
CTTATATTATGGGACGGAGGGAGT	0.28	1.71	1.99
CTTATATTATGGGACGGAGGGAG	0.28	0	0.28
CTTATATTATGGGACGGAGG	0	0.69	0.69
TTATATTATGGGACGGAGGG	0.28	0	0.28
TTATATTATGGGACGGAGG	0.28	0	0.28
TTATATTATGGGACGGAGGGA	5.32	0	5.32
TTATATTATGGGACGGAGGGAGT	0.28	0	0.28
TTATATTATGGGACGGAGGGAG	2.52	0	2.52
TATATTATGGGACGGAGG	0	0.34	0.34
TATATTATGGGACGGAGGGAG	16.53	1.37	17.9
TATATTATGGGACGGAGGGAGT	0.84	0	0.84
ATATTATGGGACGGAGGGAGT	26.89	1.37	28.26
ATATTATGGGACGGAGGGAGTA	0.56	0	0.56
ATATTATGGGACGGAGGGAG	0.56	0	0.56
ATATTATGGGACGGAGGGA	0.28	0	0.28
TATTATGGGACGGAGGGAGTA	5.88	1.03	6.91
TATTATGGGACGGAGGGAGT	0.28	0	0.28
TATTATGGGACGGAGGGAGTAC	0.28	0	0.28
ATTATGGGACGGAGGGAGTA	1.12	0	1.12
ATTATGGGACGGAGGGAGTAC	10.08	1.03	11.11
TTATGGGACGGAGGGAGTAC	0.28	0	0.28

```
      GTGATTCAGGAGCTGGGAAATACTCCCTCCGTCTCATAATATAAGAATGTTTTTGACACTACACTGTCAAAAATGTTCTTATATTATGGGACGGAGGGAGTACCTGCCCGGGCGGCCGC
      GTGATTCAGGAGCTGGGAAATACTCCCTCCGTCTCATAATATAAGAATGTTTTTGACACTACACTGTCAAAAATGTTCTTATATTATGGGACGGAGGGAGTACCTGCCCGGGCGGCCGC
      GTGATTCAGGAGCTGGGAAATACTCCCTCCGTCTCATAATATAAGAATGTTTTTGACACTACACTGTCAAAAATGTTCTTATATTATGGGACGGAGGGAGTACCTGCCCGGGCGGCCGC
-71.40 ........((.((((((...(((((((((((((((((((((((((((((((((((((......))))))))))))))))))))))))))))))))))))).....)))).))..)).. []
```

RC_tae-MIR1120

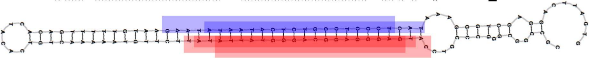

	VVI1 Leaves_trimmed	VVI3 Fruit_trimmed	VVI2 Flower_trimmed	Total
CGCATGTAGCATCATCAAGAT	0.52	0	0	0.52
TGTAGCATCATCAAGATTCAC	0.52	0	0	0.52
GTAGCATCATCAAGATTCAC	2.62	0.44	0	3.06
GTAGCATCATCAAGATTCACA	2.36	0	0	2.36
GTAGCATCATCAAGATTCA	0.79	0	0	0.79
TAGCATCATCAAGATTCACAT	3.41	0	0	3.41
ATGAGAATCTTGATGATGCTG	0.26	7.53	1.36	9.15
TGAGAATCTTGATGATGCTGCA	0	2.21	0.91	3.12
TGAGAATCTTGATGATGCTG	0	0.44	0.91	1.35
TGAGAATCTTGATGATGCTGC	2.36	72.18	16.3	90.84
TGAGAATCTTGATGATGCTGCATC	0.26	0	0	0.26
TGAGAATCTTGATGATGCTGCAT	1.05	0	0	1.05
GAGAATCTTGATGATGCTGC	0	1.77	0	1.77
AGAATCTTGATGATGCTGC	0	0.89	0	0.89
AGAATCTTGATGATGCTGCA	7.09	3.1	0.91	11.1
AGAATCTTGATGATGCTGCAT	1782.39	268.36	104.13	2154.88
GAATCTTGATGATGCTGCAT	1.05	0.44	0	1.49
CTTGATGATGCTGCATCGGCA	1.84	0	0.91	2.75
TTGATGATGCTGCATCGGCA	0.26	0	0	0.26

```
      TTGTTTGCGCATGTAGCATCATCAAGATTCACATGCAAATGCACGGTGGTGATGTTATCTCTGCCAAACTTTTCAAGGATGAGAATCTTGATGATGCTGCATCGGCAATA
      TTGTTTGCGCATGTAGCATCATCAAGATTCACATGCAAATGCACGGTGGTGATGTTATCTCTGCCAAACTTTTCAAGGATGAGAATCTTGATGATGCTGCATCGGCAATA
-42.00 ....((((..(((((((((((((((((((.(((..........(((((.(((...))).)))))..............))).)))))))))))))))))))..))))..   []
```

RC_vvi-MIR172a

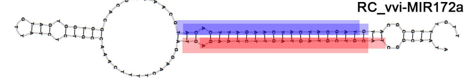

Sequence	VVI1 Leaves_trimmed	VVI3 Fruit_trimmed	VVI2 Flower_trimmed	Total
CAGGTGTAGCATCATCAAGAT	4.2	0.44	0	4.64
CAGGTGTAGCATCATCAAGATT	0.26	0	0	0.26
TGTAGCATCATCAAGATTCAC	0.52	0	0	0.52
GTAGCATCATCAAGATTCACA	2.36	0	0	2.36
GTAGCATCATCAAGATTCA	0.79	0	0	0.79
GTAGCATCATCAAGATTCAC	2.62	0.44	0	3.06
TAGCATCATCAAGATTCACAT	3.41	0	0	3.41
TGAGAATCTTGATGATGCTGC	2.36	72.18	16.3	90.84
TGAGAATCTTGATGATGCTGCA	0	2.21	0.91	3.12
TGAGAATCTTGATGATGCTG	0	0.44	0.91	1.35
TGAGAATCTTGATGATGCTGCATC	0.26	0	0	0.26
TGAGAATCTTGATGATGCTGCAT	1.05	0	0	1.05
GAGAATCTTGATGATGCTGC	0	1.77	0	1.77
AGAATCTTGATGATGCTGCAT	1782.39	268.36	104.13	2154.88
AGAATCTTGATGATGCTGCA	7.09	3.1	0.91	11.1
AGAATCTTGATGATGCTGC	0	0.89	0	0.89
GAATCTTGATGATGCTGCAT	1.05	0.44	0	1.49
CTTGATGATGCTGCATCGGCA	1.84	0	0.91	2.75
TTGATGATGCTGCATCGGCA	0.26	0	0	0.26

```
       TTGTTTGCAGGTGTAGCATCATCAAGATTCACATGCGAAAGCCCGGCGCGGCAGCGATGCCATCTTCGCTGCCTCAAGTTTTGGGGTTGAGAATCTTGATGATGCTGCATCGGCAATA
-61.20 ....((((.(((((((((((((((((((.....(((...(((((.((((((((((........))))))))....)).))))).))).)))))))))))))))))))))).)))).. []
```

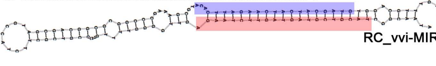

RC_vvi-MIR172b

Sequence	ZMA1 Leaves_trimmed	run1	run2	ZMA3 Male inflorescence(Tassels)_trimmed	GSM306487	GSM306488	Total
GCCTACCTCTGATAGCCAAGGA	0.26	0	0	0	0	0	0.26
ATAGCCAAGGATGACTTGCCTA	0	16.38	113.31	0	0	0	129.69
TAGCCAAGGATGACTTGCCTA	0.53	98.3	18072.89	0.24	0.35	0.83	18173.14
TAGCCAAGGATGACTTGCC	0.26	0	14.16	0	0	0	14.42
TAGCCAAGGATGACTTGCCT	0.26	0	368.26	0	0	0	368.52
TAGCCAAGGATGACTTGC	0	0	0	0.24	0	0	0.24
AGCCAAGGATGACTTGCCTA	0	0	0	0	0.17	0.28	0.45
AGGCAGTCTCCTTGGCTAGC	0.26	0	0	0	0	0	0.26
GGCAGTCTCCTTGGCTAGC	0	0	28.33	0.24	0.17	0.14	28.88
GGCAGTCTCCTTGGCTAG	0	0	14.16	0	0	0.14	14.3
GGCAGTCTCCTTGGCTAGCC	0	0	70.82	0	0	0	70.82

AATAAGGGCCTACCTCTGATAGCCAAGGATGACTTGCCTATGTCCTTTGTTTACAAAGGACCTGAATGTGTGGACCATTGAGAGGTTCATAGGCAGTCTCCTTGGCTAGCCTGAGTGGCCCCTATTGC

-59.00 ((((.(((((...(((...((((((((((.(((.((((((((.(((((.....(((.((.((.........)).)).)))))))))..)))))))))))))))))))))....))).))))).)))).. []

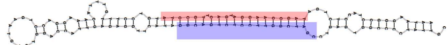

RC_zma-MIR169d

	ZMA1 Leaves_trimmed	run1	run2	GSM306487	ZMA3 Male inflorescence(Tassels)_trimmed	GSM306488	Total
AGGGCCTGCCTCTGATAGCCAAGGA	0.26	0	0	0	0	0	0.26
AGGGCCTGCCTCTGATAGCCAA	0.26	0	0	0	0	0	0.26
ATAGCCAAGGATGACTTGCCTA	0	16.38	113.31	0	0	0	129.69
TAGCCAAGGATGACTTGCCTA	0.53	98.3	18072.89	0.35	0.24	0.83	18173.14
TAGCCAAGGATGACTTGCC	0.26	0	14.16	0	0	0	14.42
TAGCCAAGGATGACTTGCCT	0.26	0	368.26	0	0	0	368.52
TAGCCAAGGATGACTTGC	0	0	0	0	0.24	0	0.24
AGCCAAGGATGACTTGCCTA	0	0	0	0.17	0	0.28	0.45
CCTTTGTGTTGGTTCGTAGGCA	0	0	0	0	0	0.14	0.14
AGGCAGTCTCCTTGGCTAGC	0.26	0	0	0	0	0	0.26
GGCAGTCTCCTTGGCTAGC	0	0	28.33	0.17	0.24	0.14	28.88
GGCAGTCTCCTTGGCTAG	0	0	14.16	0	0	0.14	14.3
GGCAGTCTCCTTGGCTAGCC	0	0	70.82	0	0	0	70.82

```
      AATAAGGGCCTGCCTCTGATAGCCAAGGATGACTTGCCTATGTCCTTTGTTTACAAAGGATCAGAATTGTGGACCTTTGTGTTGGTTCGTAGGCAGTCTCCTTGGCTAGCCTGAGTGGCCCCTATTGC
-62.60 ((((.(((((...(((...((((((((((.(((.((((((((..((.....((((((((.(((.....))).))))))))..))..)))))))))))))))))))))))....))).))))).))))..  []
```

RC_zma-MIR169e

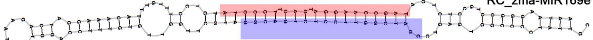

	GSM852281	GSM852280	GSM825573	GSM769285	GSM769282	GSM769284	Total
GATTGTCATGTGTCATGTTCTGA	0.07	0	0	0	0	0	0.07
ATTGTCATGTGTCATGTTCTGATT	0.07	0	0	0	0	0	0.07
GTCATGTGTCATGTTCTGATTGG	0.07	0	0	0	0	0	0.07
CATGTGTCATGTTCTGATTGGTCA	0.35	0.28	0	0	0	0	0.63
ATGTGTCATGTTCTGATTGGT	0	0	0.39	0	0	0	0.39
ATGTGTCATGTTCTGATTGGTCAA	0.14	0	0	0	0	0	0.14
GTGTCATGTTCTGATTGGT	0	0	0	0.29	0	0	0.29
GTGTCATGTTCTGATTGGTCAA	0.07	0	0	0	0	0	0.07
TGTCATGTTCTGATTGGTCAA	0	0	0	0.29	0.32	0	0.61
TGTCATGTTCTGATTGGTC	0	0	0.39	0	0	0	0.39
TGTCATGTTCTGATTGGTCAATG	0.07	0	0	0	0	0	0.07
TGTCATGTTCTGATTGGTCAATGA	0	0	0.78	0	0	0	0.78
GTCATGTTCTGATTGGTCAATGAA	0.07	0	0	0.59	0.64	0	1.3
TCATGTTCTGATTGGTCAATGAAA	0	0	0.39	0.29	0	0	0.68
TCATGTTCTGATTGGTCAATG	0	0	0.39	0	0	0	0.39
TCATGTTCTGATTGGTCAATGA	0	0	0	0.29	0	0	0.29
CATGTTCTGATTGGTCAATGAA	0	0	0	0	0.32	0	0.32
ATAACAATGCATTTCATCTTT	0	0	0.39	0	0	0	0.39
TAATCAGAACATGACACGTGACA	0	0	0.78	0	0	0	0.78
TAATCAGAACATGACACGTGACAA	0	0	0.39	0	0.32	1.15	1.86
AATCAGAACATGACACGTGA	0	0	0.39	0	0	0	0.39
AATCAGAACATGACACGTGACA	0	0	1.56	0	0.32	0	1.88
AATCAGAACATGACACGTGAC	0.35	0	1.17	0.59	0	0.76	2.87
AATCAGAACATGACACGTGACAAT	1.87	0	4.28	3.52	6.08	15.67	31.42
AATCAGAACATGACACGT	0	0	0.39	0	0	0	0.39
AATCAGAACATGACACGTGACAA	0	0	0	0	0	0.76	0.76
ATCAGAACATGACACGTGACAA	0	0	0.39	0	0	0	0.39
ATCAGAACATGACACGTGAC	0	0	0.39	0	0	0	0.39
ATCAGAACATGACACGTGACAATC	0	0	0.39	1.17	2.56	1.53	5.65
ATCAGAACATGACACGTGACA	0	0	0	0	0	0.38	0.38
ATCAGAACATGACACGTGACAAT	0	0	0	0.29	0	0	0.29
TCAGAACATGACACGTGACAATC	0	0	0.39	0	0	0.38	0.77
TCAGAACATGACACGTGACAA	0	0	0	0.29	0	0.38	0.67
TCAGAACATGACACGTGACAAT	0	0	0.39	0	0	0.38	0.77
CAGAACATGACACGTGACAA	0	0	0.39	0	0	0	0.39
CAGAACATGACACGTGACAATCG	0.07	0	0	0	0	0	0.07
CAGAACATGACACGTGACA	0	0	0.39	0	0	0	0.39
CAGAACATGACACGTGACAATCGT	0.07	0.14	0	0	0	0	0.21
CAGAACATGACACGTGAC	0	0	0.39	0	0	0	0.39

```
       TGATTGTCATGTGTCATGTTCTGATTGGTCAATGAAAATAACAATGCATTTCATCTTTCATGTAATCAGAACATGACACGTGACAATCGTCATTTAGTAAGAA
-44.17 .(((((((((((((((((((((((((....((((((..................)))))).)))))))))))))))))))))))))................   []
```

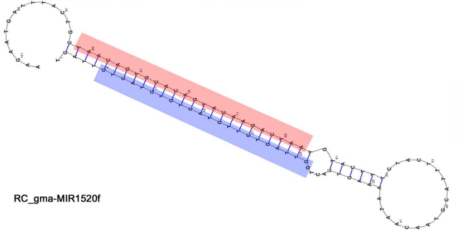

RC_gma-MIR1520f

	GSM852281	GSM852280	GSM825573	GSM769284	GSM769282	GSM769285	Total
ACTGTCATGTGTCATGTTCTGATT	0.07	0	0	0	0	0	0.07
GTCATGTGTCATGTTCTGATTGG	0.07	0	0	0	0	0	0.07
GTCATGTGTCATGTTCTGATTGGA	0.07	0	0	0	0	0	0.07
CATGTGTCATGTTCTGATTGGA	0.07	0	0	0	0	0	0.07
CATGTGTCATGTTCTGATTGGATG	0	0.14	0	0	0	0	0.14
ATGTGTCATGTTCTGATTGGATGT	0	0	0.39	0	0	0	0.39
ATGTGTCATGTTCTGATTGGATG	0.07	0	0	0	0	0	0.07
TGTGTCATGTTCTGATTGGATGTC	0.56	1.84	0.39	0.38	0.64	0.88	4.69
TGTGTCATGTTCTGATTGGATG	0	0	0	0.38	0	0	0.38
GTGTCATGTTCTGATTGGATGTCA	0.49	0	0.39	0	0.96	1.76	3.6
GTGTCATGTTCTGATTGGATGTC	0	0	0.39	0	0	0	0.39
TGTCATGTTCTGATTGGATGTC	0	0	0	0	0	0.59	0.59
TGTCATGTTCTGATTGGATGTCAAC	0	0	0	0	0	0.29	0.29
TGTCATGTTCTGATTGGATGTCAA	0	0	0	1.53	8.64	3.81	13.98
TGTCATGTTCTGATTGGATG	0	0	0.78	0	0	0	0.78
GTCATGTTCTGATTGGATGTCAAC	0.83	1.69	0.39	0.38	0	0.29	3.58
GTCATGTTCTGATTGGATGTCAA	0	0	0	0	0	0.29	0.29
TCATGTTCTGATTGGATGTC	0	0	0.39	0	0	0.29	0.68
TCATGTTCTGATTGGATGTCA	0	0	0	0.38	0.64	0	1.02
TCATGTTCTGATTGGATGTCAA	0	0	0	0.38	0.32	0	0.7
TCATGTTCTGATTGGATGTCAAC	0	0	0	0	0.32	0	0.32
CATGTTCTGATTGGATGTCAA	0	0	0	0	0.32	0	0.32
ATGTTCTGATTGGATGTCAAC	0.07	0	0	0	0	0.29	0.36
TCATTGACTAATCAGAACATGACA	0.07	0	0.39	0	0	0	0.46
CTAATCAGAACATGACACGTGAC	0	0	0	0.38	0	0	0.38
TAATCAGAACATGACACGTGACA	0	0	0.78	0	0	0	0.78
TAATCAGAACATGACACGTGACAA	0	0	0.39	1.15	0.32	0	1.86
AATCAGAACATGACACGTGACAAT	1.87	0	4.28	15.67	6.08	3.52	31.42
AATCAGAACATGACACGTGAC	0.35	0	1.17	0.76	0	0.59	2.87
AATCAGAACATGACACGTGA	0	0	0.39	0	0	0	0.39
AATCAGAACATGACACGTGACA	0	0	1.56	0	0.32	0	1.88
AATCAGAACATGACACGTGACAA	0	0	0	0.76	0	0	0.76
AATCAGAACATGACACGT	0	0	0.39	0	0	0	0.39
ATCAGAACATGACACGTGACAATC	0	0	0.39	1.53	2.56	1.17	5.65
ATCAGAACATGACACGTGACAA	0	0	0.39	0	0	0	0.39
ATCAGAACATGACACGTGAC	0	0	0.39	0	0	0	0.39
ATCAGAACATGACACGTGACA	0	0	0	0.38	0	0	0.38
ATCAGAACATGACACGTGACAAT	0	0	0	0	0	0.29	0.29
ATCAGAACATGACACGTGACAATCA	0	0	0	0	0	0.59	0.59
TCAGAACATGACACGTGACAATC	0	0	0.39	0.38	0	0	0.77
TCAGAACATGACACGTGACAAT	0	0	0.39	0.38	0	0	0.77
TCAGAACATGACACGTGACAA	0	0	0	0.38	0	0.29	0.67
TCAGAACATGACACGTGACAATCA	0.07	0	0	0	0	0	0.07
CAGAACATGACACGTGACAATCAT	0	0	0.39	0	0	0	0.39
CAGAACATGACACGTGACA	0	0	0.39	0	0	0	0.39
CAGAACATGACACGTGACAA	0	0	0.39	0	0	0	0.39
CAGAACATGACACGTGACAATCA	0	0	0	0	0	0.29	0.29
CAGAACATGACACGTGAC	0	0	0.39	0	0	0	0.39
AGAACATGACACGTGACAATCATC	0	0	0.39	0.38	0	0	0.77
AGAACATGACACGTGACAATCATCA	0	0	0	0	0	0.29	0.29
AACATGACACGTGACAATCATCA	0	0	0	0	0	0.29	0.29
ACATGACACGTGACAATCATCATC	0	0	0	0	0	0.29	0.29
ACATGACACGTGACAATCAT	0	0	0.39	0	0	0	0.39
ATGACACGTGACAATCATCATCCA	0.14	0	0.39	0	0	0	0.53

```
      TTCTTATTGGTTGATGACTGTCATGTGTCATGTTCTGATTGGATGTCAACACCATGAAAGATGAAATACATTGCTGTTTTCATTGACTAATCAGAACATGACACGTGACAATCATCATCCAATAAGAA
-64.01 .((((((((.((((((.((((((((((((((((((((((((.................(((((((.(((....))))))))))..))))))))))))))))))))))))))).)))))).)))))))))).   []
```

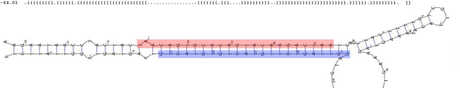

RC_gma-MIR1520k

Sequence	GSM852281	GSM852280	GSM825573	GSM769285	Total
CGGTTTTGTAAGGTTGAGTTAGGC	0.14	0.14	0	0	0.28
TATCCGATGTGGGACTCTTAACAC	0	0.14	0	0	0.14
CCGATGTGGGACTCTTAACAC	0	0.28	0	0	0.28
CGATGTGGGACTCTTAACACACCC	0.07	0	0	0	0.07
CGATGTGGGACTCTTAACAC	0.28	0.28	0	0	0.56
CTAACTCAACCTTACAAAACCGG	0	0.14	0	0	0.14
TAACTCAACCTTACAAAACCGG	0.14	0	0.39	0	0.53
TAACTCAACCTTACAAAACCGGCT	0	0	0	0.29	0.29

CTCACAAGCCGGTTTTGTAAGGTTGAGTTAGGCCTCAACTTTATAAATATATTGTCAGACCATGTCCTATCCGATGTGGGACTCTTAACACACCCCCTCACGATCAGCACTATTAGGTTTGGTTCGTAGACATAAATGGGGGGTGGCCCGATAGCGGAAACCTGATAGCATGTGGTTCAATGGCTCTGATACCATGTTGAAATTTGAGGTCTAACTCAACCTTACAAAACCGGCTTGTGAG

-116.33 (((((((((((((((((((((((((((((((((((((.(((...(((((..((((((((((((..(((((((...((((..........(((((((((((((((.(((....)))))))).))).))........))))))).))))....))))..............)))..)).))).)))))))..))))).))).)))))))))))))))))))))))))))))))))))))) []

RC_gma-MIR1527

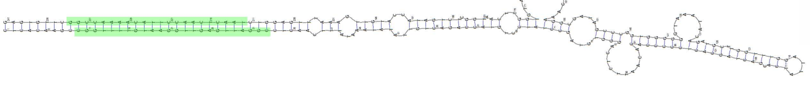

	GSM852281	GSM852280	GSM769285	Total
TGATGACAGAGGAAGAGAGAGC	2.71	0	0	2.71
GATGACAGAGGAAGAGAGAGC	8.4	0.14	0	8.54
ATGACAGAGGAAGAGAGAGC	0.14	0	0	0.14
TGACAGAGGAAGAGAGAGC	0.07	0	0	0.07
GACAGAGGAAGAGAGAGC	0	0	0.29	0.29
GACAGAGGAAGAGAGAGCAC	0	0	1.17	1.17
GACAGAGGAAGAGAGAGCA	0	0	0.59	0.59
GTGCTCTCTCTCTTCTGTC	0	0.42	0	0.42
GTGCTCTCTCTCTTCTGTCA	2.43	7.77	0	10.2
GCTCTCTCTCTTCTGTCAA	2.57	0	0	2.57

```
      TGAATGTGATGATGACAGAGGAAGAGAGAGCACACTCCCAACAAAGGCAAAGACTCCTTTAACCATTCCCGGGTTGTGCTCTCTCTCTTCTGTCAACATGAGATAT
-44.50 ........(((.(((((((((.((((((((((((..(((......((.((((....))))..)).......))).))))))))))))))))))))).)))...... []
```

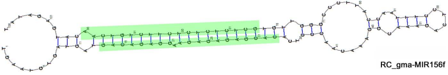

RC_gma-MIR156f

Sequence	GSM852281	GSM852280	Total
CTACTAGGTGAAACTGCCACA	0.76	0	0.76
GGTGAAACTGCCACATGAT	0.07	0	0.07
GGTGAAACTGCCACATGATCTGAT	11.38	0	11.38
GGTGAAACTGCCACATGATC	16.1	0	16.1
GTGAAACTGCCACATGATC	0.07	0	0.07
GTGAAACTGCCACATGATCTGAT	0	0.28	0.28
GAAACTGCCACATGATCTGATC	0.07	0.14	0.21
CCACATGATCTGATCTTTCCA	0	0.42	0.42
GTTAAGATCATGCTGGCAGCTTCA	0.35	0	0.35
TAAGATCATGCTGGCAGCTTC	0.07	0	0.07
AAGATCATGCTGGCAGCTTC	0.28	0	0.28
AAGATCATGCTGGCAGCTTCAA	0.56	7.2	7.76
AAGATCATGCTGGCAGCTT	0	0.14	0.14
AAGATCATGCTGGCAGCT	0	0.14	0.14
AAGATCATGCTGGCAGCTTCA	0	914.6	914.6
AGATCATGCTGGCAGCTTCAA	0.14	0.71	0.85
AGATCATGCTGGCAGCTTCA	0	11.01	11.01
GATCATGCTGGCAGCTTCA	0	13.7	13.7

```
      TCATGCGGCCAGCAACTACTAGGTGAAACTGCCACATGATCTGATCTTTCCACGGCAAGTGAGGGAAGTTAAGATCATGCTGGCAGCTTCAACTGGTAGTGGTGCATGA
-54.70 (((((.(((....((((((((.((((.((((((((((((((...((((((.((.....)).))))))...)))))))).)))))).)))).)))))))))))))))))   []
```

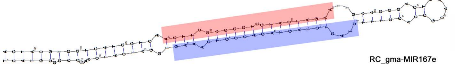

RC_gma-MIR167e

	GSM852281	GSM852280	Total
CTACTAGGTGAAACTGCCACA	0.76	0	0.76
GGTGAAACTGCCACATGATC	16.1	0	16.1
GGTGAAACTGCCACATGATCTGAT	11.38	0	11.38
GGTGAAACTGCCACATGAT	0.07	0	0.07
GTGAAACTGCCACATGATCTGAT	0	0.28	0.28
GTGAAACTGCCACATGATC	0.07	0	0.07
GAAACTGCCACATGATCTGATC	0.07	0.14	0.21
CCACATGATCTGATCTTTCCA	0	0.42	0.42
GATCTTTCCACAGCAAGT	0.76	0.56	1.32
GATCTTTCCACAGCAAGTGAGG	0	0.14	0.14
GTTAAGATCATGCTGGCAGCTTCA	0.35	0	0.35
TAAGATCATGCTGGCAGCTTC	0.07	0	0.07
AAGATCATGCTGGCAGCTTC	0.28	0	0.28
AAGATCATGCTGGCAGCTTCA	0	914.6	914.6
AAGATCATGCTGGCAGCTTCAA	0.56	7.2	7.76
AAGATCATGCTGGCAGCT	0	0.14	0.14
AAGATCATGCTGGCAGCTT	0	0.14	0.14
AGATCATGCTGGCAGCTTCA	0	11.01	11.01
AGATCATGCTGGCAGCTTCAA	0.14	0.71	0.85
GATCATGCTGGCAGCTTCA	0	13.7	13.7

```
       TCATGCGGCCAACAACTACTAGGTGAAACTGCCACATGATCTGATCTTTCCACAGCAAGTGAGGGAAGTTAAGATCATGCTGGCAGCTTCAACTGGTAGTGGTGCATGA
-55.30 ((((((.(((....((((((((.((((.((((((((((((((...((((((.((.....)).))))))...))))))))).)))))).)))).)))))))))))))))))   []
```

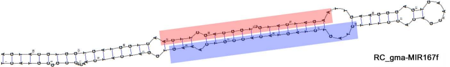

RC_gma-MIR167f

	GSM852280	GSM852281	GSM825573	GSM769285	Total
GGTGTGAGTATCTACGCCTCCGAG	0.28	0	0	0	0.28
GGTGTGAGTATCTACGCCT	1.84	0	0	0	1.84
GGTGTGAGTATCTACGCCTC	1.69	1.18	0	0	2.87
GGTGTGAGTATCTACGCC	0.42	0.07	0	0	0.49
GGTGTGAGTATCTACGCCTCC	383.52	594.7	0	0	978.22
GGTGTGAGTATCTACGCCTCCG	0	0.07	0	0	0.07
GTGTGAGTATCTACGCCTC	0	0.14	0	0	0.14
GTGTGAGTATCTACGCCTCC	6.21	5	0	0	11.21
GTGTGAGTATCTACGCCTCCG	0.99	0.14	0	0	1.13
TGTGAGTATCTACGCCTCCG	0.85	0.28	0	0	1.13
TGTGAGTATCTACGCCTCC	1.55	1.6	0	0	3.15
TGTGAGTATCTACGCCTC	0	0.07	0	0	0.07
GTGAGTATCTACGCCTCC	0	0.14	0	0	0.14
GAGTATCTACGCCTCCGAGGC	0.28	0	0	0	0.28
TCTCAGAGGCGAAGACACTCG	0.56	0.07	0	0	0.63
CTCAGAGGCGAAGACACTCGCA	0.56	0	0	0	0.56
TCAGAGGCGAAGACACTCGC	3.39	4.86	0	0	8.25
TCAGAGGCGAAGACACTCGCA	85.15	80.85	0	0	166
CAGAGGCGAAGACACTCGC	0	0.21	0	0	0.21
CAGAGGCGAAGACACTCGCA	1.98	3.12	0	0	5.1
AGAGGCGAAGACACTCGCACC	0	0	0.39	0.29	0.68
AGAGGCGAAGACACTCGCA	0	0.14	0	0	0.14

GGTGTGAGTATCTACGCCTCCGAGGCTTGAGATCTCATAGTATCTCTCTCAGAGGCGAAGACACTCGCACC
GGTGTGAGTATCTACGCCTCCGAGGCTTGAGATCTCATAGTATCTCTCTCAGAGGCGAAGACACTCGCACC
-37.90 ((((((((.(((.((((((.((((...((((((((...)).)))))))))).)))))).))).))))))))) []

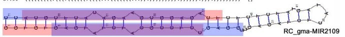

RC_gma-MIR2109

Sequence	GSM852281	GSM852280	GSM769285	GSM769282	GSM769284	GSM825573	Total
AACCGTCTTAGAATGTCTACA	0.21	0	0	0	0	0	0.21
AACCGTCTTAGAATGTCTACAT	0	0.42	0	0	0	0	0.42
ACCGTCTTAGAATGTCTACATTT	0.28	0	0	0	0	0	0.28
CCGTCTTAGAATGTCTACA	0.07	0	0	0	0	0	0.07
CGTCTTAGAATGTCTACATTTTA	0.21	0	0	0	0	0	0.21
GTCTTAGAATGTCTACATTTTAA	0.07	0	0	0	0	0	0.07
ACACTCTTAGACAGTTCTTTGAAG	0	0	0.29	0	0	0	0.29
TAGATTGTAAACATTCTAAGACGG	0.49	0	0	0.64	0.38	0	1.51
AGATTGTAAACATTCTAAG	0	0	0	0	0	0.78	0.78
GATTGTAAACATTCTAAGACGGTT	0.14	0	0	0	0	0	0.14
ATTGTAAACATTCTAAGACGGTTT	0	0.28	0	0	0	0	0.28
TGTAAACATTCTAAGACGGTTTCT	0	0	0.29	0	0	0	0.29
AAACATTCTAAGACGGTTTCTGAA	0	0	0	0.32	0	0	0.32

GATTTTTTGAAAAACCGTCTTAGAATGTCTACATTTTAAAACGATTTTTGGAAAAATCATCTTAGAATTCTAAATTTATTTTAATTTTTTAAAAAAAACAAACACTCTTAGACAGTTCTTTGAAGAATCGTCTTAGATTGTAAACATTCTAAGACGGTTTCTGAAAAAATC

-53.85 ((((((((...((((((((((((((((.((((.(((((.(((((((((.(((.(((..(((.(((............((((((....))))))..........))).)))..))).))).))))))))).))))).)))).)))))))))))))))))))...)))))))) []

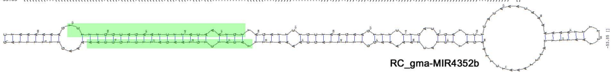

RC_gma-MIR4352b

Sequence	GSM769284	GSM769285	GSM825573	GSM852281	GSM852280	GSM769282	Total
AACAGCGGAAGAACCTTCTT	0.38	0	0	0	0	0	0.38
CAGCGGAAGAACCTTCTT	0	0.29	0	0	0	0	0.29
CAGCGGAAGAACCTTCTTCCGT	0	0	0.39	0	0	0	0.39
AGAACCTTCTTCCGTGCGATCTCG	0	0	0	0.28	0	0	0.28
GAACCTTCTTCCGTGCGATCTCGC	0	0	0	0.14	0	0	0.14
AACCTTCTTCCGTGCGATCTCGCG	0	0	0	0	1.55	0	1.55
AACCTTCTTCCGTGCGATCTC	0	0	0	0	0.14	0	0.14
ACCTTCTTCCGTGCGATCTCG	0	0	0	0	0.42	0	0.42
ACCTTCTTCCGTGCGATCTCGCGG	0	0	0	0.42	0	0	0.42
CCTTCTTCCGTGCGATCTCGCGGA	0	0	0	0	0.28	0	0.28
CTTCTTCCGTGCGATCTCGCGGA	0	0	0.39	0	0	0	0.39
CAAGATGGCACGGAAGAAGGT	0	0	0.39	0	0	0	0.39
ATGGCACGGAAGAAGGTTCT	0.38	0	0	0	0	0	0.38
ATGGCACGGAAGAAGGTTCTTCC	0.38	0	0	0	0	0	0.38
TGGCACGGAAGAAGGTTCTTC	0	0	0.39	0	0	0.32	0.71
GCACGGAAGAAGGTTCTTCCGC	0	0.29	0	0	0	0	0.29

```
       AACAGCGGAAGAACCTTCTTCCGTGCGATCTCGCGGAAGAACCATCTTCCGTGGCTTCCTGGGGAAGAAGTTCTTCCGCAAGATGGCACGGAAGAAGGTTCTTCCGCAGTT
-81.00 ....(((((((((((((((((((((((.((((.((((((((((..((((((.(((....))).)))))).)))))))))).)))).)))))))))))))))))))))))....  []
```

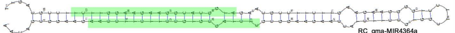

RC_gma-MIR4364a

Sequence	GSM852281	GSM825573	GSM769285	GSM769284	GSM769282	GSM852280	Total
CGTCTTTGAAAGTTCACAAACA	0.07	0	0	0	0	0	0.07
AAACAAATACGACGTTGCTTGC	0	0.39	0	0	0	0	0.39
ATACGACGTTGCTTGCACCGTCTT	0	0	0.29	0	0	0	0.29
TTTGTTTGTGAACTTTCAAAGACG	0.21	0.39	0.29	0.38	0.32	0	1.59
TTGTTTGTGAACTTTCAAAGACGT	0	0.39	1.17	0.76	1.28	0	3.6
TGTTTGTGAACTTTCAAAGACGTT	0.07	0.39	2.64	2.29	0.96	0	6.35
TGTTTGTGAACTTTCAAAGACGT	0	0	0.59	0.38	0	0	0.97
TGTTTGTGAACTTTCAAAGACG	0	0	0	0.76	0	0	0.76
GTTTGTGAACTTTCAAAGACGT	0	0	0	0.76	0	0	0.76
TTTGTGAACTTTCAAAGACGTT	0.14	0	0	0	0	0	0.14
TTTGTGAACTTTCAAAGACG	0	0	0.29	0	0	0	0.29
TTGTGAACTTTCAAAGACGTTGCT	0.56	0	0.88	0.76	0.32	0	2.52
TGTGAACTTTCAAAGACGTTGCTT	0.07	0	0	0	0	0.14	0.21
TGTGAACTTTCAAAGACGTTGC	0.97	0	0.88	0	0	0	1.85
TGTGAACTTTCAAAGACGTTGCT	0.21	0	0.29	0	0	0	0.5
TGTGAACTTTCAAAGACGTTG	0	0	0.29	0	0	0	0.29
GTGAACTTTCAAAGACGTTGC	0.14	0	0	0	0	0.28	0.42
GTGAACTTTCAAAGACGTTGCTTA	0	0	0.29	0	0	0	0.29
TGAACTTTCAAAGACGTTGCTTAC	0.28	0	0	0	0	0.71	0.99
TGAACTTTCAAAGACGTTGCTT	0	0.39	0	0	0	0	0.39
TGAACTTTCAAAGACGTTGCT	0.28	0	0	0.38	0	0	0.66
TGAACTTTCAAAGACGTTGC	0	0	0.29	0	0	0	0.29
GTTGCTTACGTAAGTACCGTCCTT	0.07	0	0	0	0	0	0.07

AAAAGACGGTGCTTACATAAGCAGCGTCTTTGAAAGTTCACAAACAAATACGACGTTGCTTGCACCGTCTTTGTTTGTGGACGTTGCTTGCACCGTCTTTGTTTGTGAACTTTCAAAGACGTTGCTTACGTAAGTACCGTCCTTG

-91.20 ....(((((((((((.((((((((((((((((((((((((((((((((...((((.(((..(((.(((((........))))).)))..))).)))))))))))))))))))))))))))))))))))))).)))))))))))))).... []

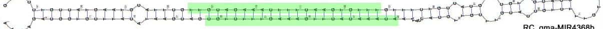

RC_gma-MIR4368b

	GSM852281	GSM852280	GSM769282	GSM769284	GSM769285	Total
TCTTCTTCTTAGTTCTTTG	0.07	0	0	0	0	0.07
TTCTTCTTAGTTCTTTGCCGAG	0	0.14	0	0	0	0.14
TCTTCTTAGTTCTTTGCCGAGAGC	0.28	0	0	0	0	0.28
TTCTTAGTTCTTTGCCGAGAGC	0	0.14	0	0	0	0.14
TCTTAGTTCTTTGCCGAGAGCT	0.07	0	0	0	0	0.07
TCTTAGTTCTTTGCCGAGAGC	0.14	0	0	0	0	0.14
TAGTTCTTTGCCGAGAGCTTA	0	0	0.32	0	0	0.32
TTTCATAAGCTCTCGGCAAAGAAC	0.07	0	0	0	0	0.07
TTCATAAGCTCTCGGCAAAGAA	0	0	0	0.38	0	0.38
CATAAGCTCTCGGCAAAGAACTAA	0.07	0	0	0	0	0.07
AGCTCTCGGCAAAGAACTAAGAAGA	0	0	0	0.38	0	0.38
AGCTCTCGGCAAAGAACTAAGAAG	0	0	0.32	0	0	0.32
CTCTCGGCAAAGAACTAAGA	0.07	0	0	0	0	0.07
CTCTCGGCAAAGAACTAAGAAGAA	0	0	0	0.76	0	0.76
CTCTCGGCAAAGAACTAAGAAGA	0	0	0	0.38	0	0.38
CTCTCGGCAAAGAACTAAGAAG	0	0	0.32	0	0	0.32
TCTCGGCAAAGAACTAAGAAGA	0.21	0	0.96	0.38	0	1.55
TCTCGGCAAAGAACTAAGAAGAA	0	0	0	0	0.29	0.29
TCTCGGCAAAGAACTAAGAAG	0.14	0	0	0	0	0.14
TCGGCAAAGAACTAAGAAGA	0.07	0	0	0	0	0.07
TCGGCAAAGAACTAAGAAG	0.07	0	0	0	0	0.07
TCGGCAAAGAACTAAGAAGAA	0.07	0	0	0	0	0.07
CGGCAAAGAACTAAGAAGAA	0.07	0	0	0	0	0.07

```
       GAACCAAATTGTTCTTCTTCTTAGTTCTTTGCCGAGAGCTTATGAAAAATATTGATGTTGTGATGTCTACAAAATTTTCATAAGCTCTCGGCAAAGAACTAAGAAGAACAGCTTGGTCC
-63.30 ..(((((.((((...((((((((((((((((((((((((((((((((.....(((((....)))))......))))))))))))))))))))))))))))))))))).))))).. []
```

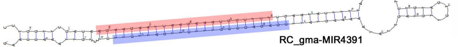

RC_gma-MIR4391

	GSM852281	GSM852280	Total
AACTGTTGTGGGGATCTACGCC	0.07	0	0.07
ACTGTTGTGGGGATCTAC	0	0.28	0.28
ACTGTTGTGGGGATCTACGCCA	0.28	0.71	0.99
ACTGTTGTGGGGATCTACGCC	7.77	13.13	20.9
ACTGTTGTGGGGATCTACGC	10.41	3.39	13.8
ACTGTTGTGGGGATCTACG	1.87	1.41	3.28
ACTGTTGTGGGGATCTACGCCACT	0.07	0	0.07
CTGTTGTGGGGATCTACGCC	0	0.28	0.28
GTTGTGGGGATCTACGCCACT	0.07	0	0.07
TCAGAGGCAAAGATACCCGCA	0.76	1.13	1.89
CAGAGGCAAAGATACCCGCAA	0.07	0.14	0.21
GAGGCAAAGATACCCGCAACA	11.8	13.7	25.5
AGGCAAAGATACCCGCAACA	0	0.14	0.14
GGCAAAGATACCCGCAACA	0.62	0.85	1.47
GGCAAAGATACCCGCAACAGT	0.07	0	0.07
GCAAAGATACCCGCAACA	0.28	0	0.28

AACTGTTGTGGGGATCTACGCCACTAAAGCAATAAGCCATAATAATAGGCACAACTTTCCTTCAGAGGCAAAGATACCCGCAACAGTT

-32.00 ((((((((((((.((((..(((.((.(((.((...(((.........)))......)).))).)).)))..)))).)))))))))))) []

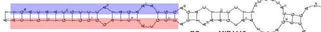

RC_gma-MIR4412

Sequence	GSM852281	GSM825573	GSM769282	GSM769284	GSM769285	GSM852280	Total
AATAAGAGAATTGTAAGTCAC	0.14	0	0	0	0	0	0.14
AATAAGAGAATTGTAAGTCACTG	0	0.39	0	0	0	0	0.39
TAAGAGAATTGTAAGTCACT	0.35	3.11	0.32	0	0	0	3.78
TAAGAGAATTGTAAGTCAC	0.07	1.56	0.96	0	0	0	2.59
TAAGAGAATTGTAAGTCACTG	266.36	80.93	29.12	24.07	17.01	5.93	423.42
TAAGAGAATTGTAAGTCA	0.07	0.39	0	0	0.29	0	0.75
TAAGAGAATTGTAAGTCACTGT	0.14	0	0.32	0	0.29	0	0.75
TAAGAGAATTGTAAGTCACTGTAT	0.07	0	0	0	0	0	0.07
AAGAGAATTGTAAGTCACTGT	1.39	1.56	0.32	0.76	0.29	1.27	5.59
AAGAGAATTGTAAGTCACTG	1.18	0.78	0	0	0.88	0	2.84
AGAGAATTGTAAGTCACTG	0.07	0	0	0	0	0.14	0.21
AGAGAATTGTAAGTCACTGTA	0.21	0	0	0	0	0	0.21
GAGAATTGTAAGTCACTGTA	0.07	0	0	0	0	0	0.07
GAGAATTGTAAGTCACTG	0.07	0	0	0	0	0	0.07
AATACAGTGACTTACAATTC	0	0	0.32	0	0	0	0.32
AATACAGTGACTTACAATTCT	0	0	0	0	0	0.14	0.14
ATACAGTGACTTACAATTCTC	0.21	0.39	0.32	0.76	0	0	1.68
TACAGTGACTTACAATTCTCT	0	0.39	0.64	0	0	0	1.03
CAGTGACTTACAATTCTCT	0.28	0	0	0	0	0	0.28
CAGTGACTTACAATTCTCTT	0.35	0	0	0	0	0	0.35
CAGTGACTTACAATTCTCTTA	0	0	0.64	0	0	0	0.64
GTGACTTACAATTCTCTTATT	0.14	0	0	0	0	0	0.14

TTAATAAGAGAATTGTAAGTCACTGTATCATGCATCTAATCAACAGTTCCTAATTAATACAGTGACTTACAATTCTCTTATTGATGA

-37.39 ((((((((((((((((((((((((((((............................)))))))))))))))))))))))))))).... []

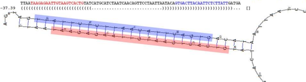

RC_gma-MIR4413

	GSM852280	GSM769284	GSM852281	Total
CATAGGAATGGGCGGAATTGG	0.56	0	0	0.56
CATAGGAATGGGCGGAATTGGG	0.71	0	0	0.71
ATAGGAATGGGCGGAATTGGG	0	0.38	0	0.38
TAGGAATGGGCGGAATTGGGAAG	0.14	0	0	0.14
AGGAATGGGCGGAATTGGGAAG	0.14	0	0	0.14
AGGAATGGGCGGAATTGGGAA	4.24	0	0.07	4.31
GGAATGGGCGGAATTGGGAAGA	0	0	0.28	0.28
GAATGGGCGGAATTGGGAA	0.42	0	0	0.42
CTTCCCAATCAGCCCATT	0.42	0	0.07	0.49
CTTCCCAATCAGCCCATTCCC	0	0	43.17	43.17
CTTCCCAATCAGCCCATTCC	0.14	0	0.14	0.28
CTTCCCAATCAGCCCATTC	1.69	0	0	1.69
CTTCCCAATCAGCCCATTCCCA	17.37	0	0	17.37
TTCCCAATCAGCCCATTC	0	0	0.07	0.07
TTCCCAATCAGCCCATTCCCA	0	0	9.72	9.72
TTCCCAATCAGCCCATTCCCAC	0.71	0	0.56	1.27
TTCCCAATCAGCCCATTCC	0	0	41.78	41.78
TTCCCAATCAGCCCATTCCC	0	0	0.07	0.07
TCCCAATCAGCCCATTCCCACA	0	0	0.14	0.14
TCCCAATCAGCCCATTCCC	0	0	0.21	0.21
TCCCAATCAGCCCATTC	0.56	0	0	0.56
TCCCAATCAGCCCATTCCCAC	2.4	0	2.01	4.41
TCCCAATCAGCCCATTCC	76.39	0	50.52	126.91
TCCCAATCAGCCCATTCCCA	153.35	0	0	153.35
CCCAATCAGCCCATTCCCACA	0.14	0	0.21	0.35
CCCAATCAGCCCATTCCCACAAAT	0.56	0	0	0.56
CCAATCAGCCCATTCCCA	0.85	0	0	0.85
GCCCATTCCCACAAATTCTGA	3.25	0	0	3.25

```
      TCAGAAATCATAGGAATGGGCGGAATTGGGAAGAAATTAAATGCATTGCACCAGCACACATTGCTTCCCAATCAGCCCATTCCCACAAATTCTGA
-39.40 ((((((......((((((((.((.((((((((......((((...(((....)))..)))).)))))))))).)))))))))......))))))  []
```

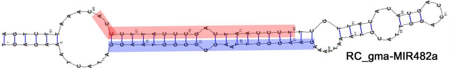

Supplement: Figure S1 — Structure- and accumulation level-based identification of plant RC-miRNAs. (PDF) [file pone.0046991.s001.pdf]
