# Supplementary figures and images for: Genome-Wide Identification of Reverse Complementary microRNA Genes in Plants
Source: PLoS One. 2012 Oct 23;7(10):e46991. doi: 10.1371/journal.pone.0046991 (PMC3479107; doi:10.1371/journal.pone.0046991)

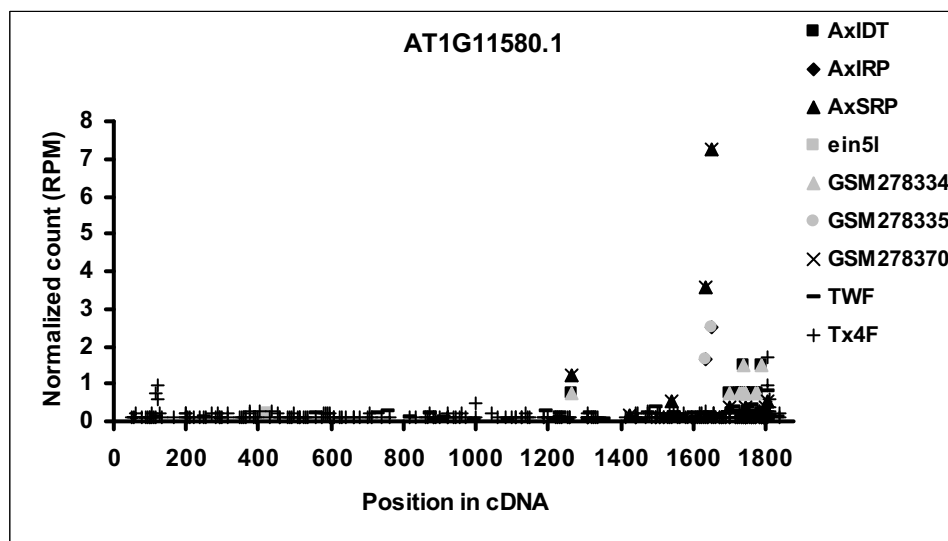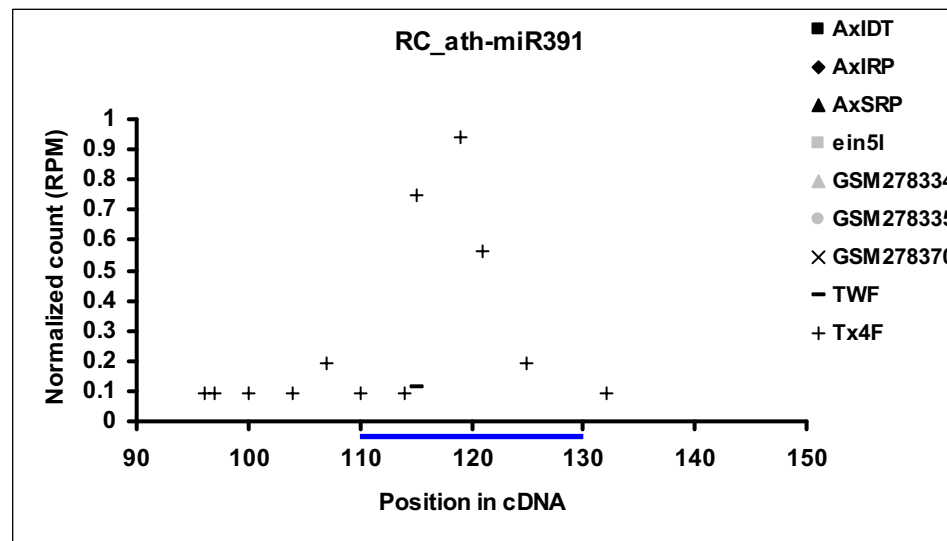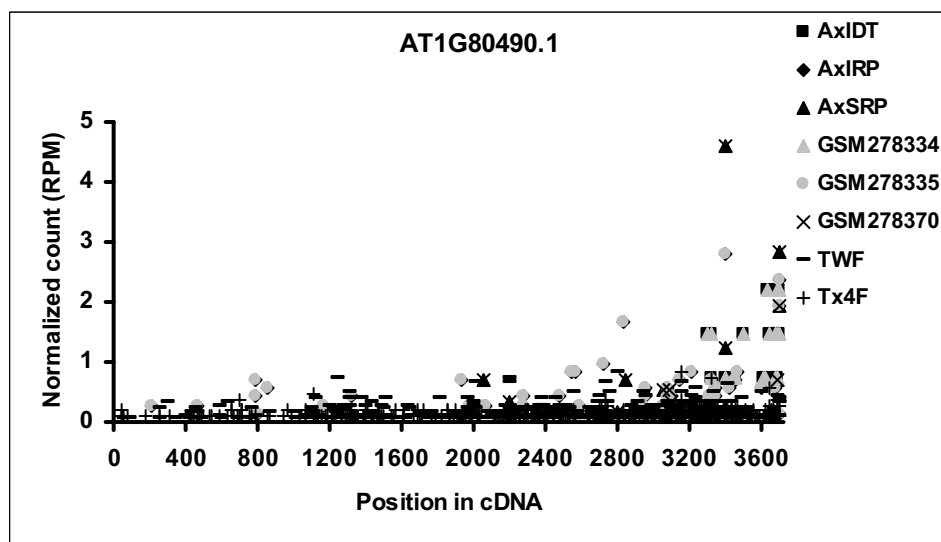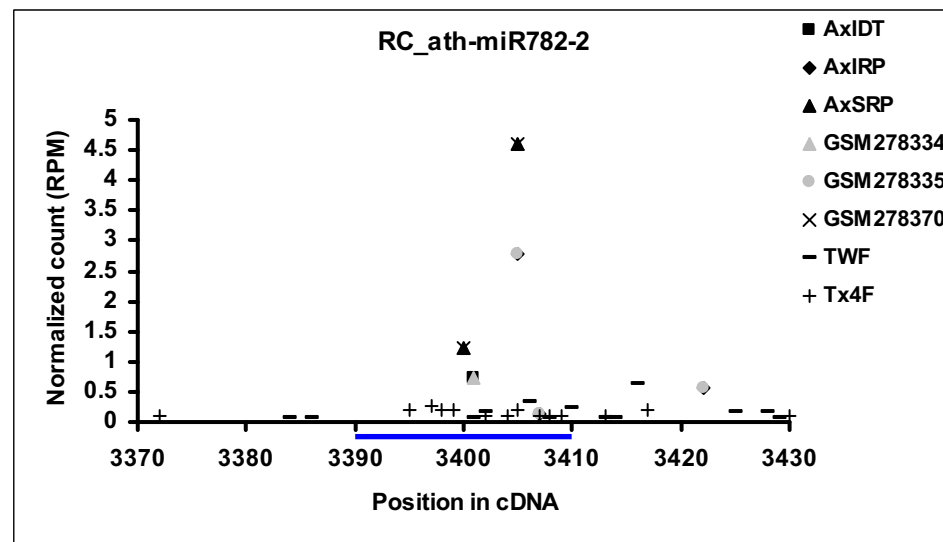

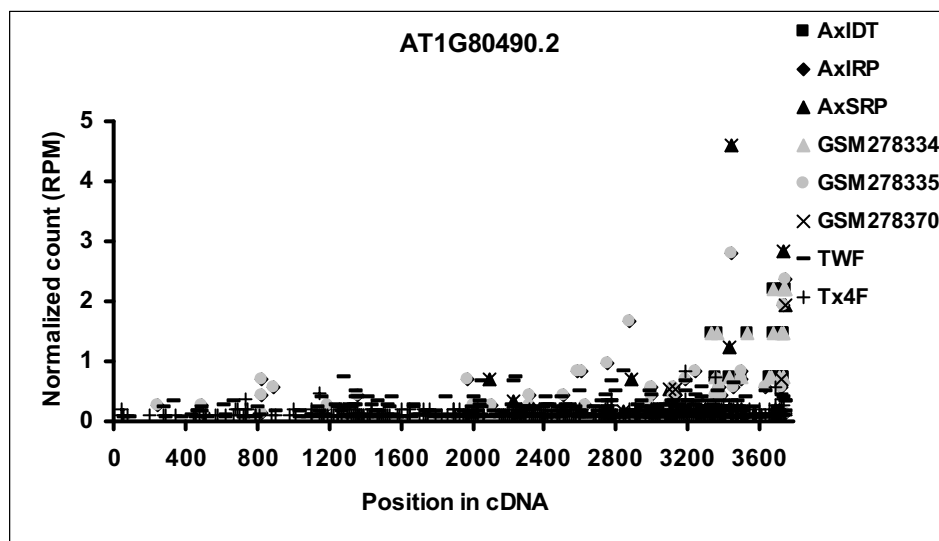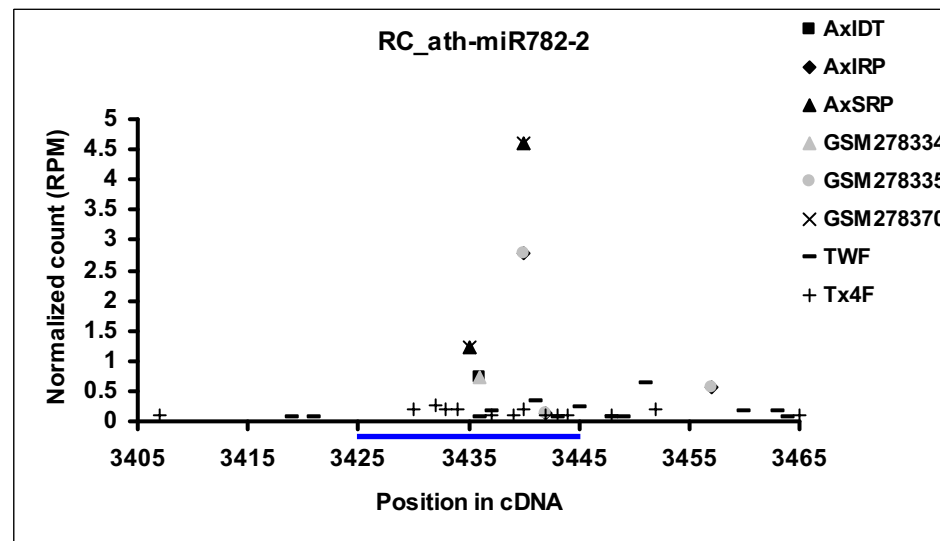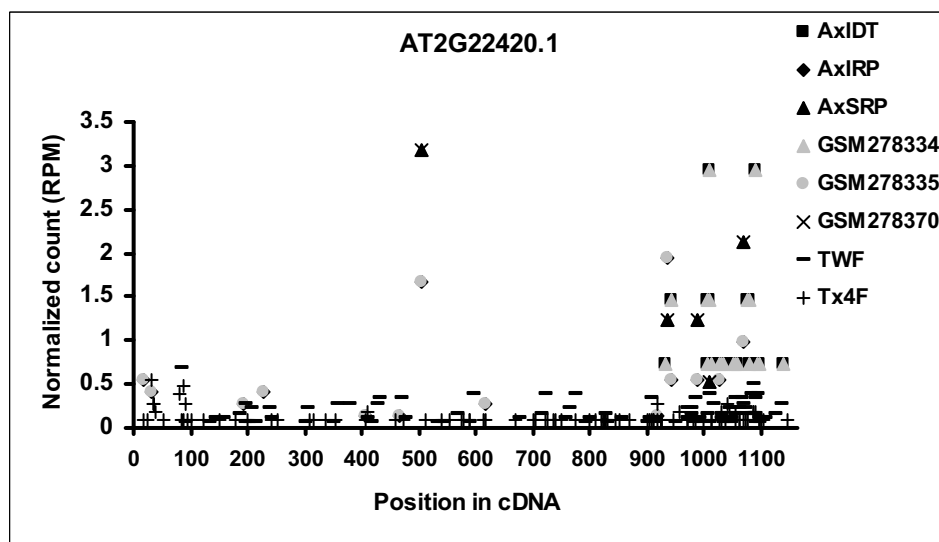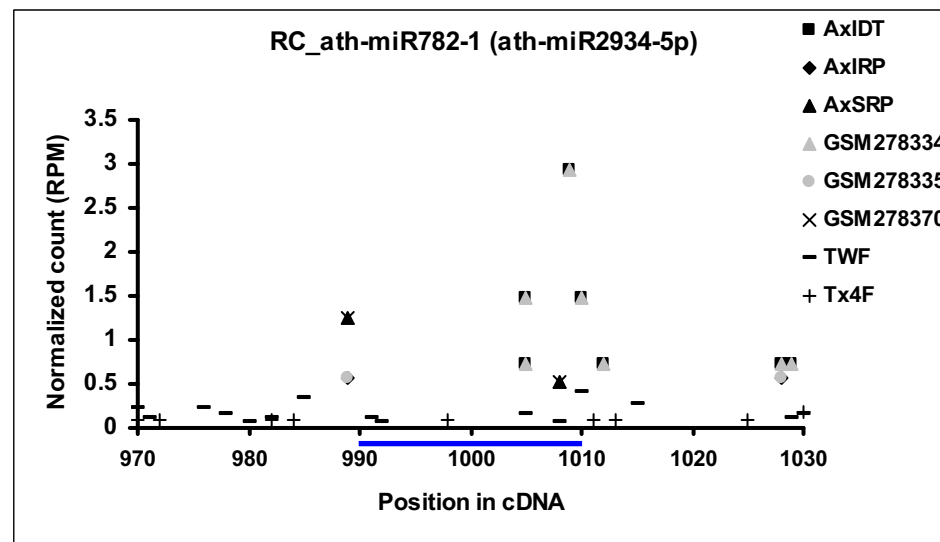

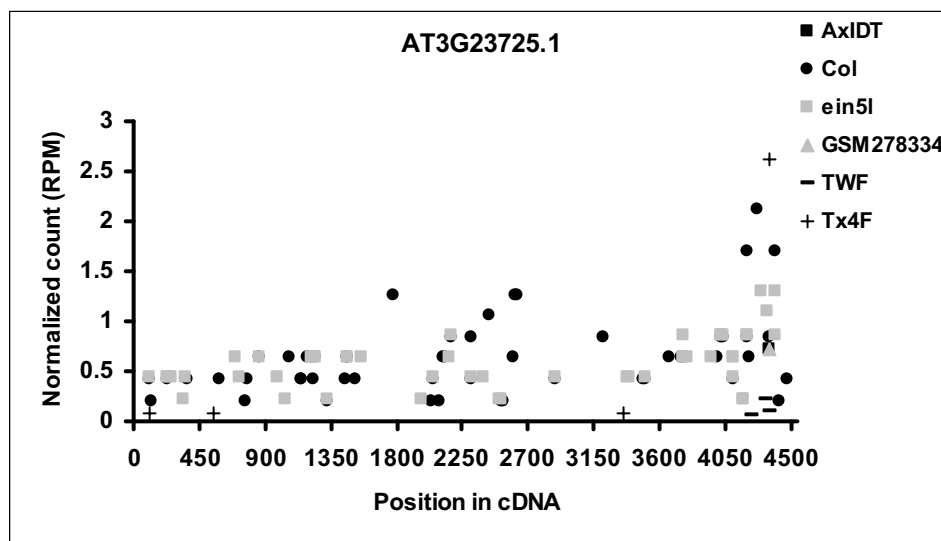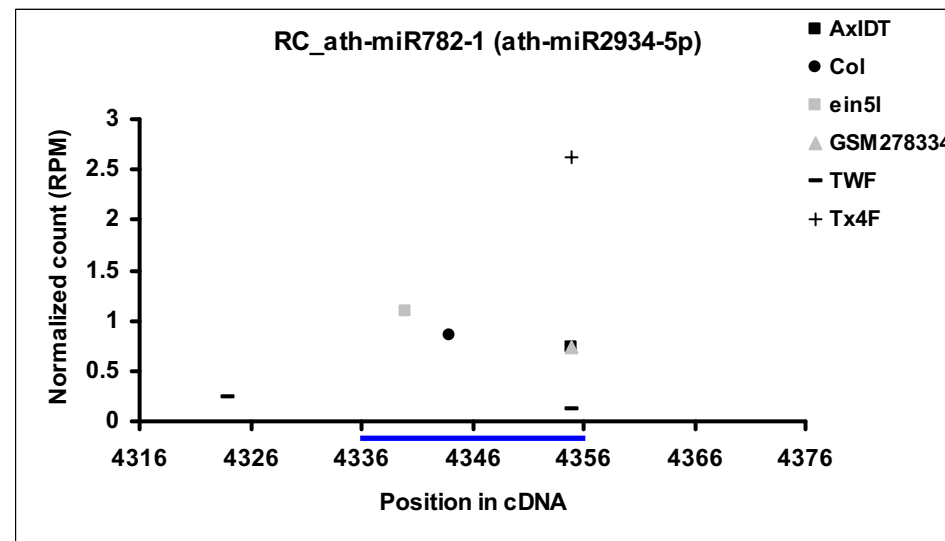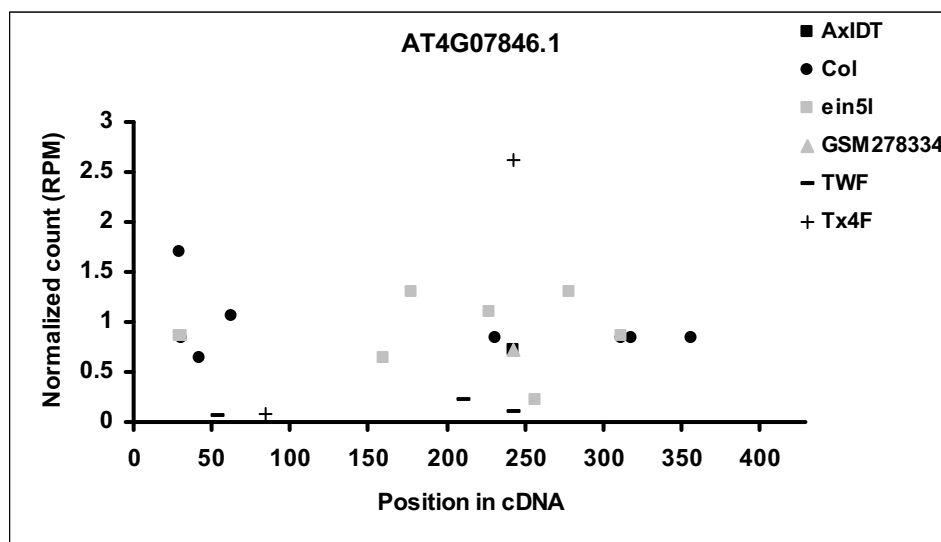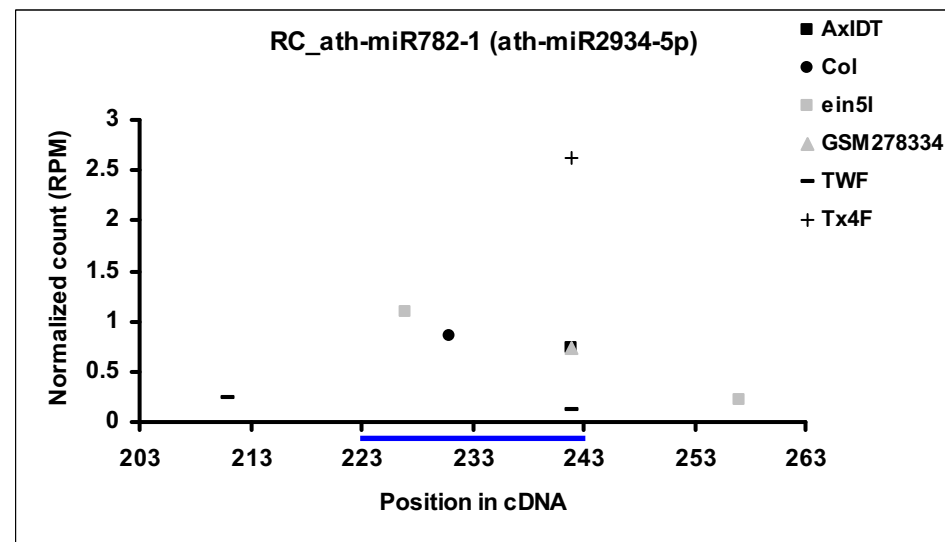

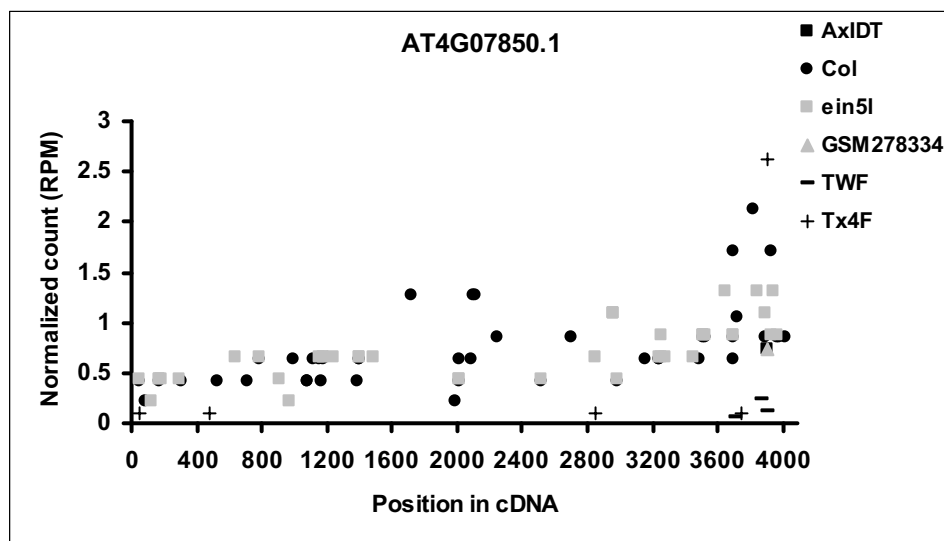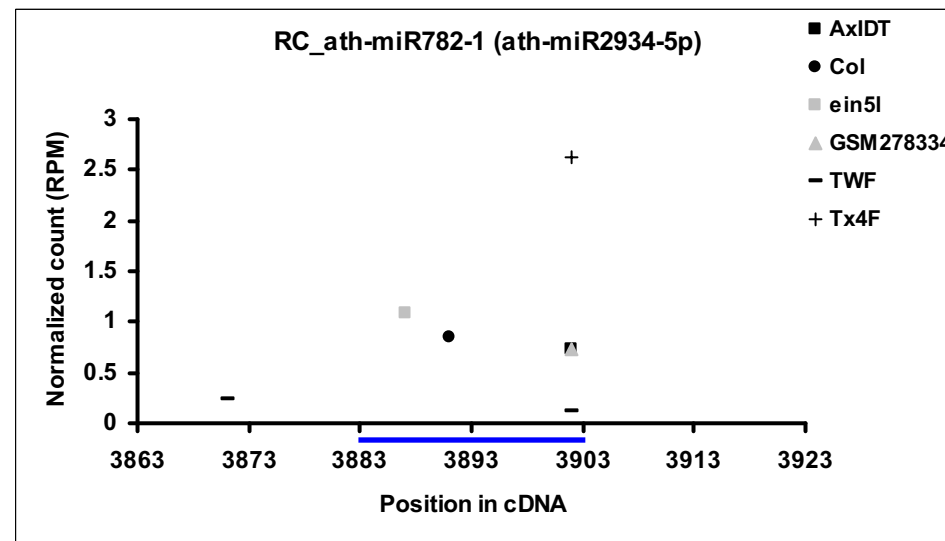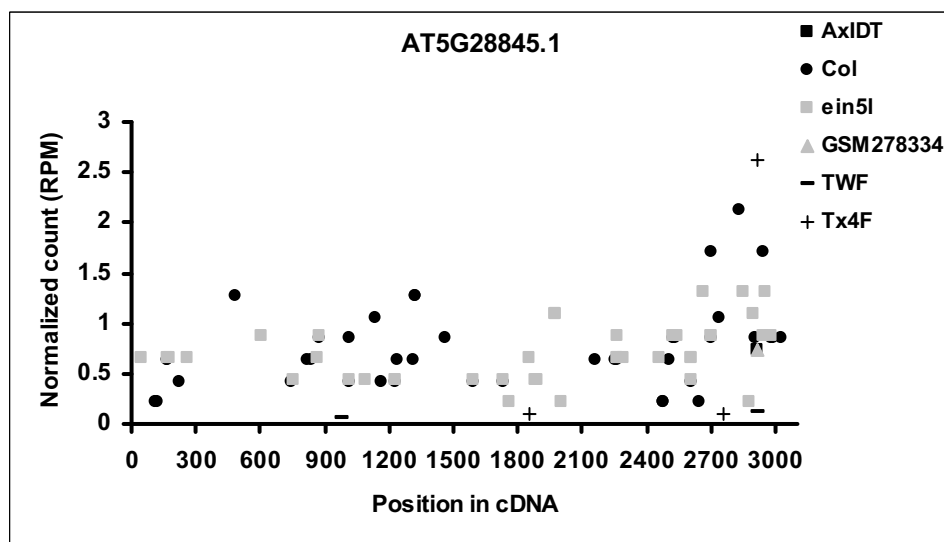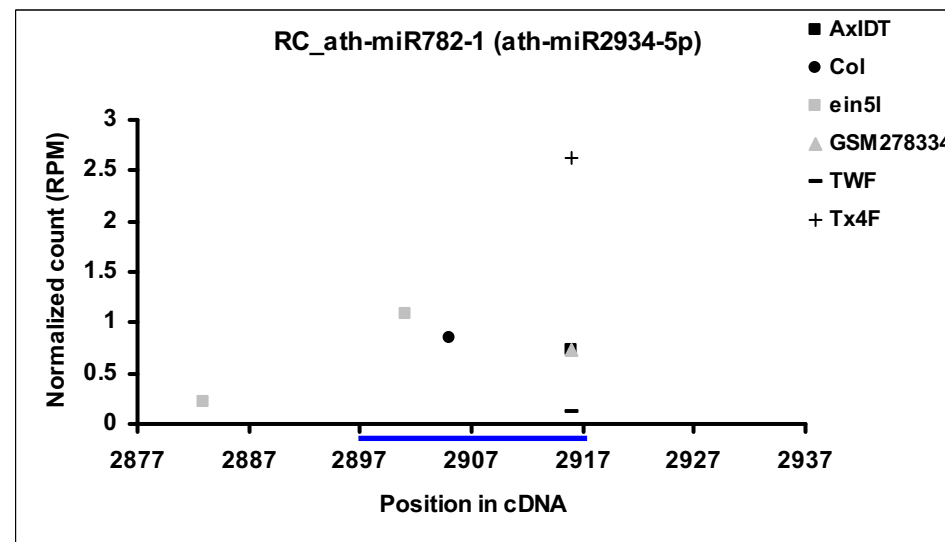

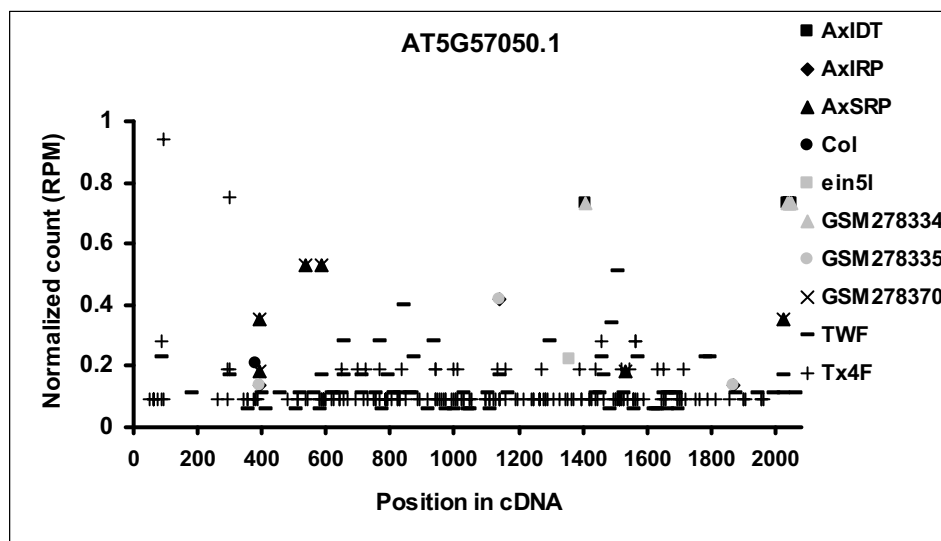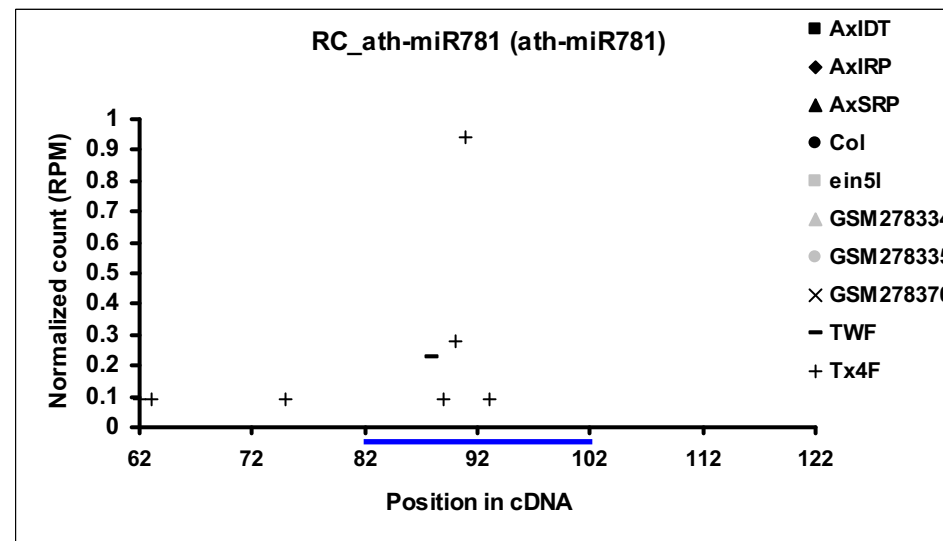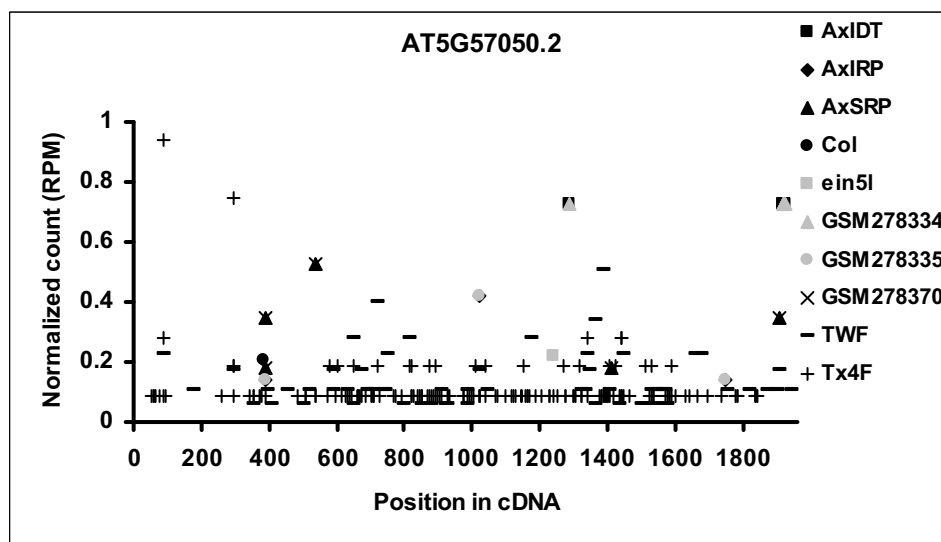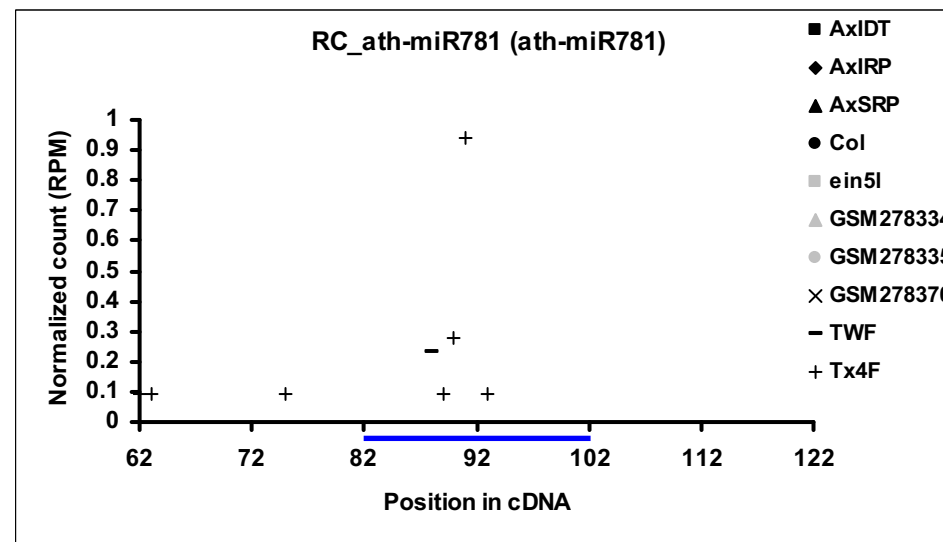

Supplement: Figure S2 — Degradome data-based validation of RC-miRNA-regulated target transcripts in Arabidopsis . (PDF) [file pone.0046991.s002.pdf]

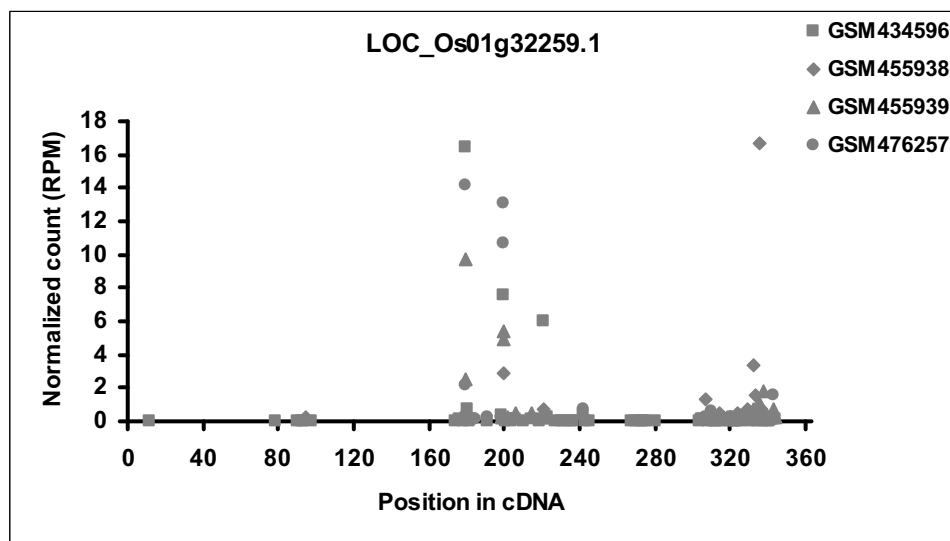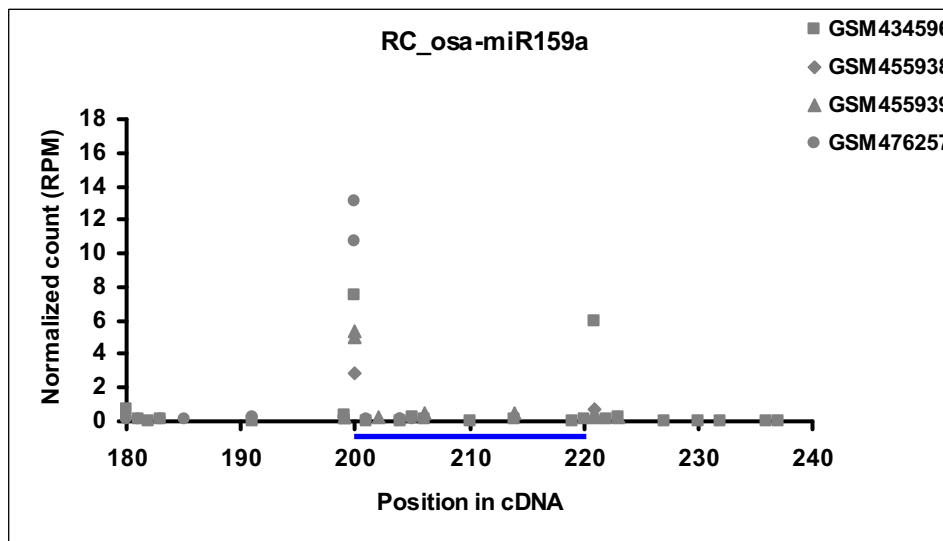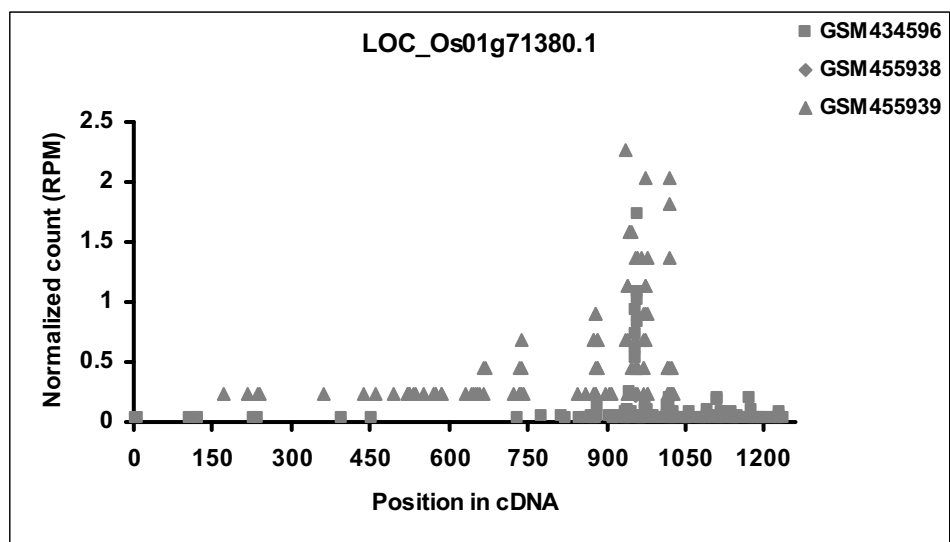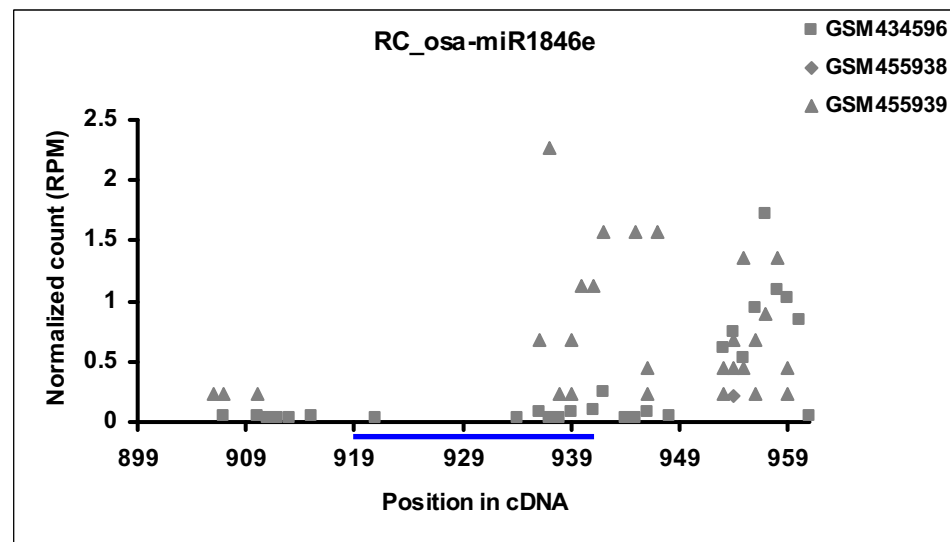

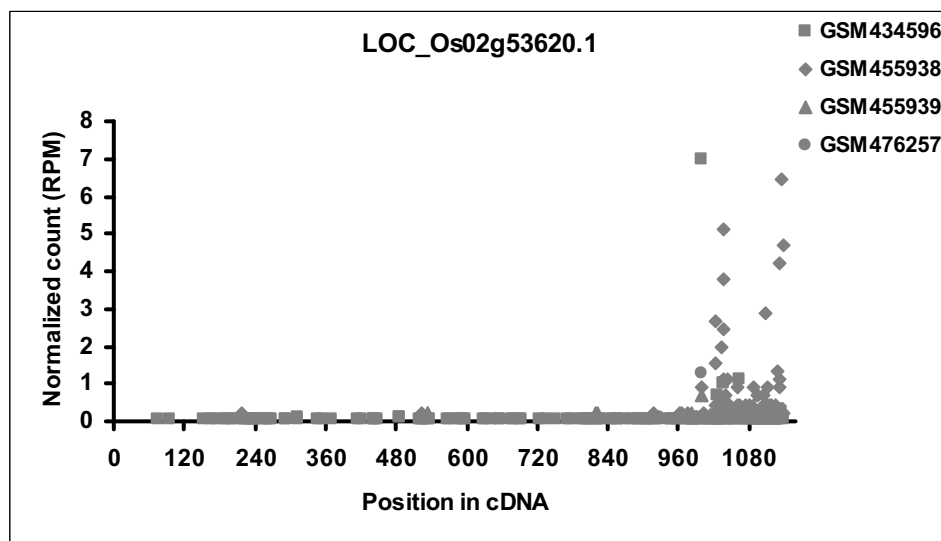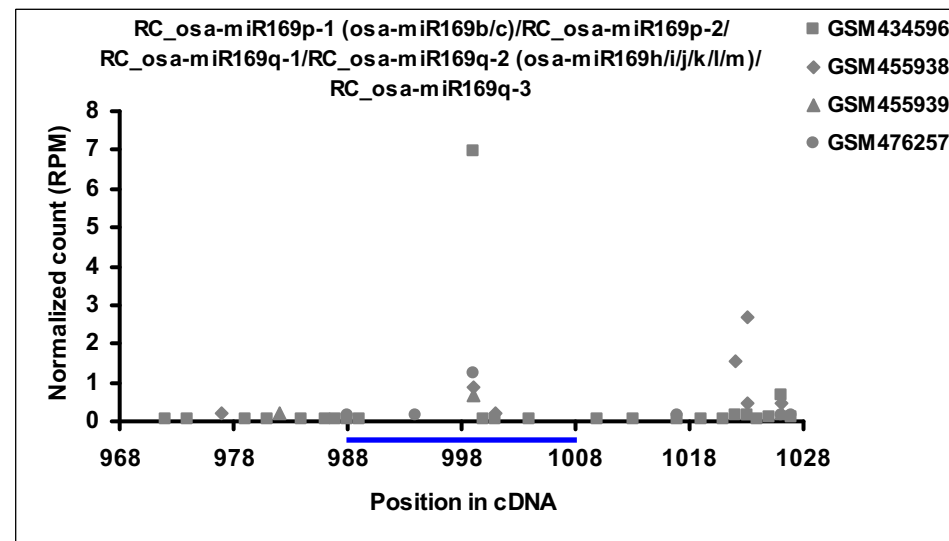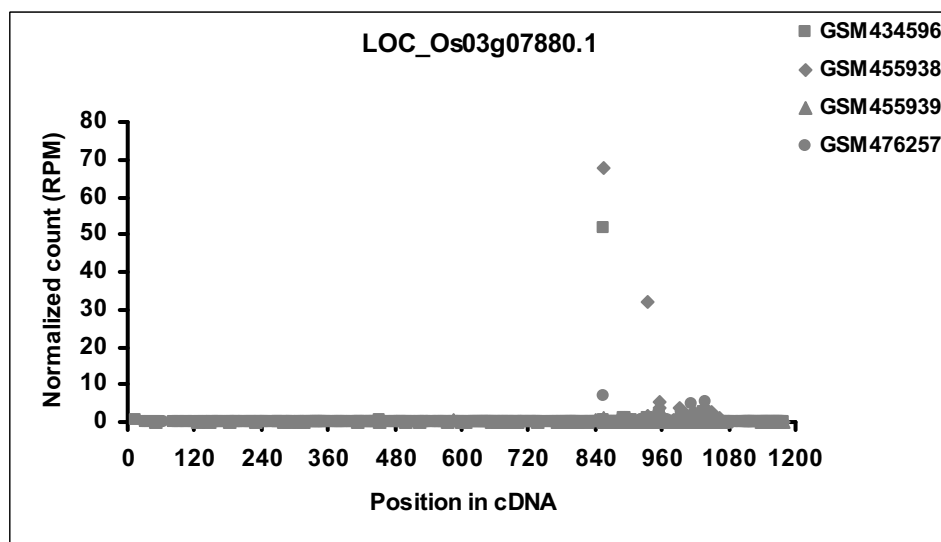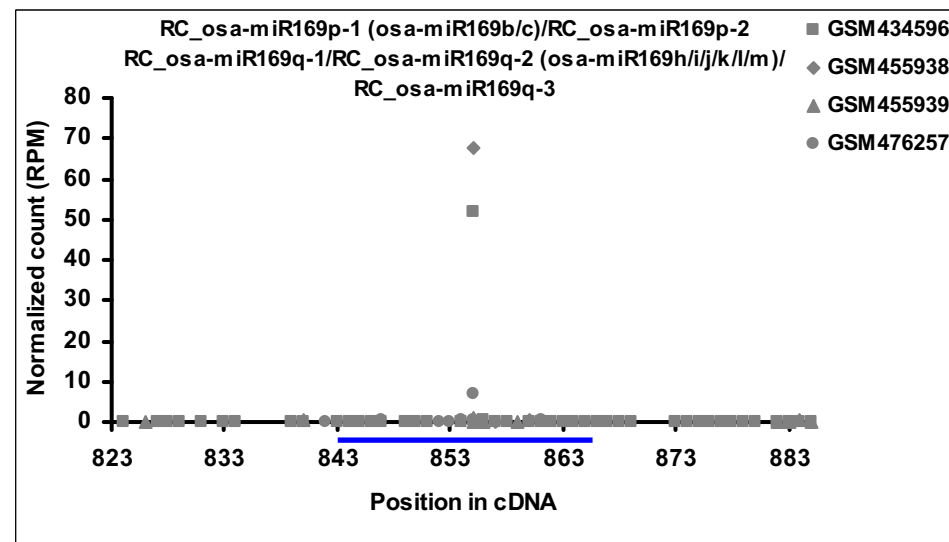

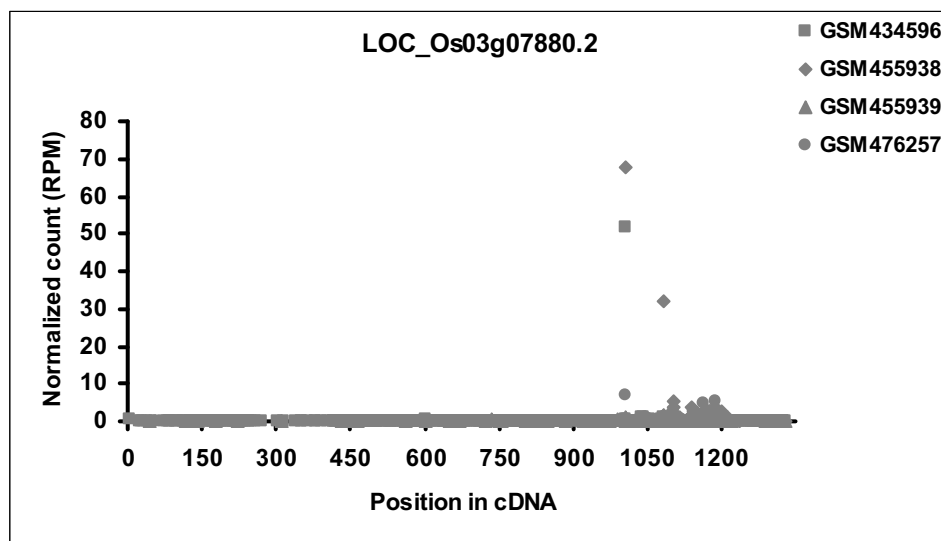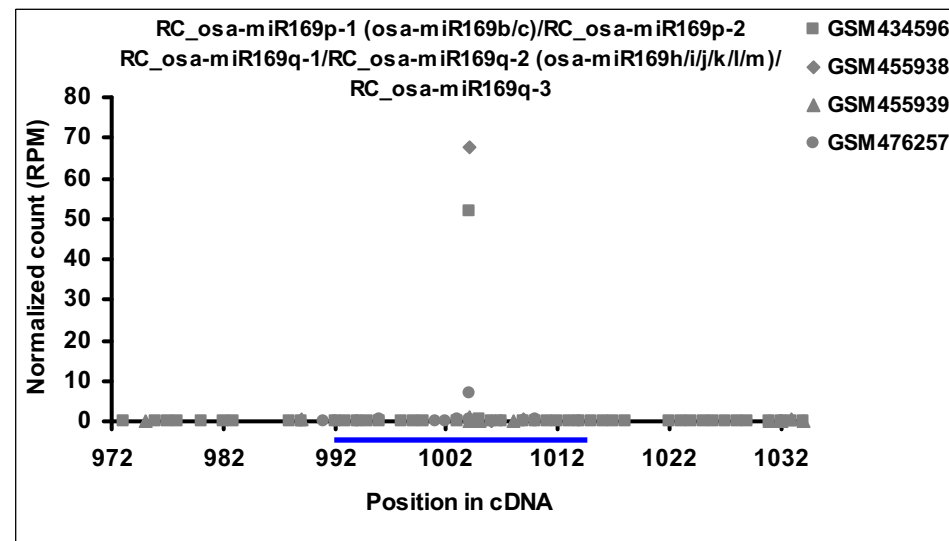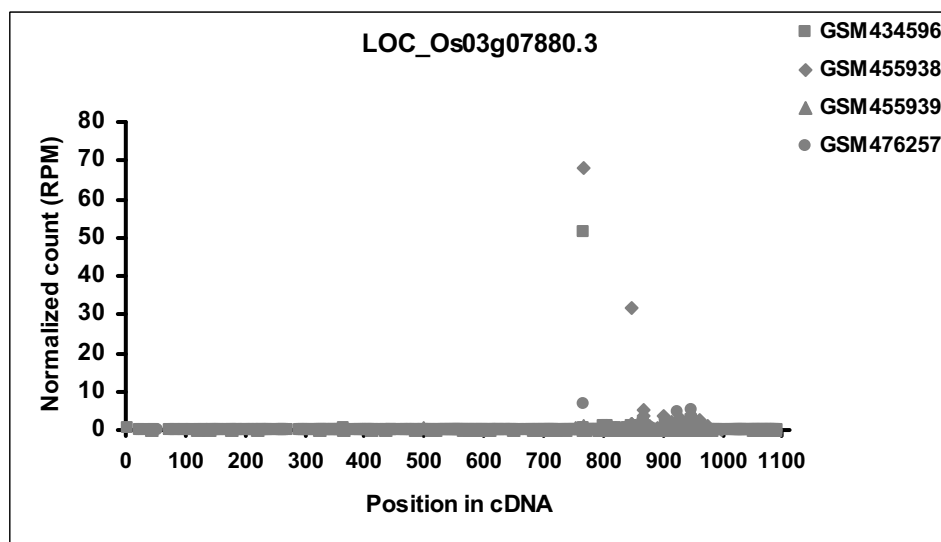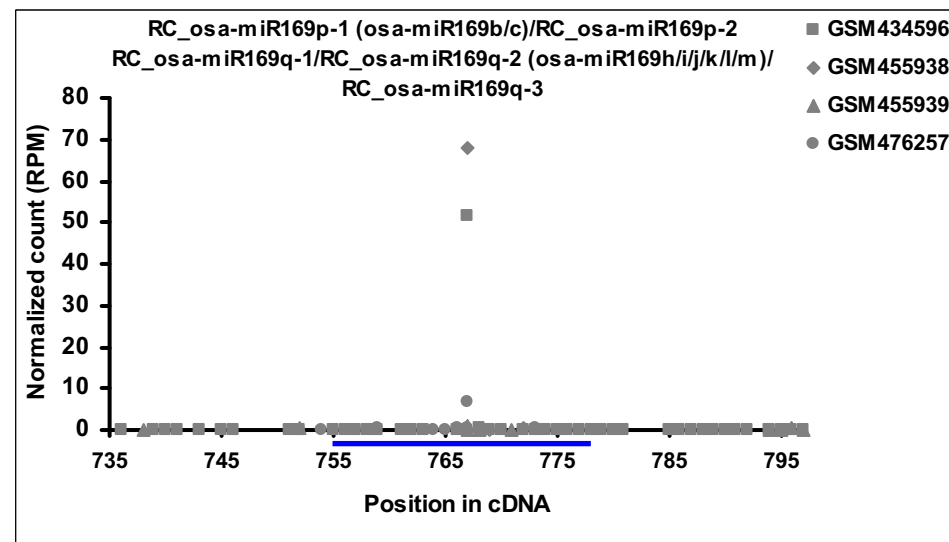

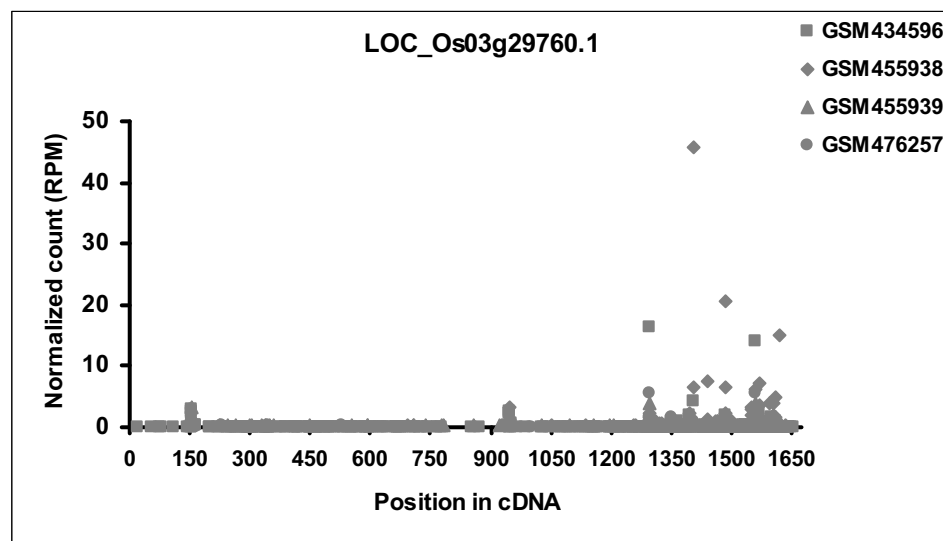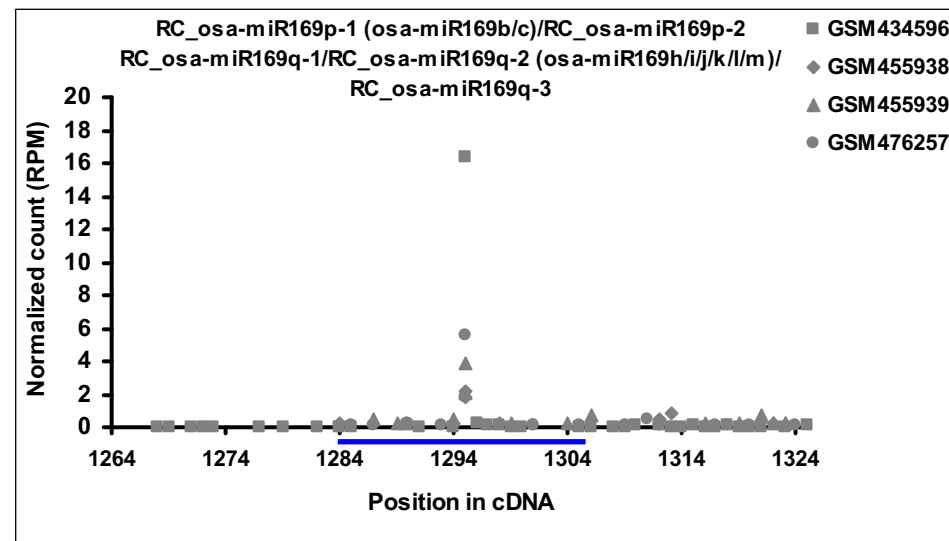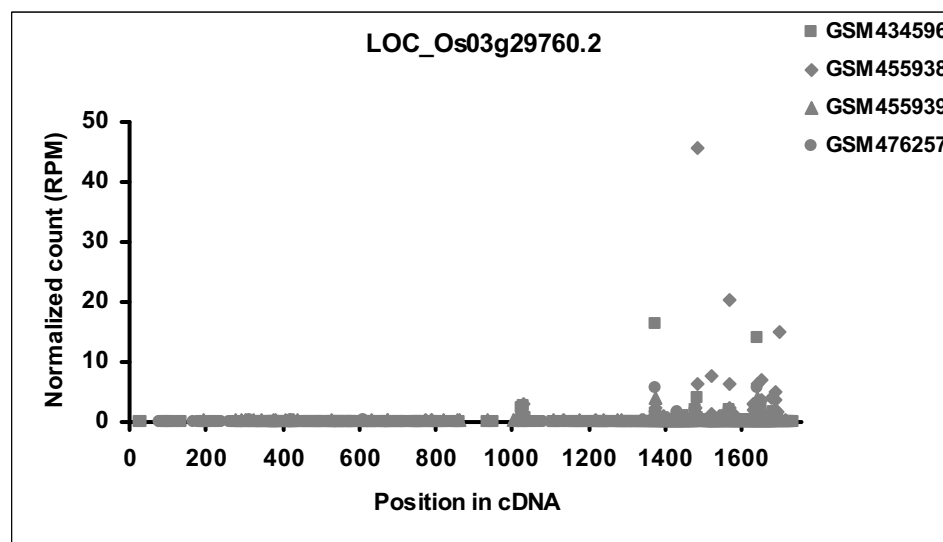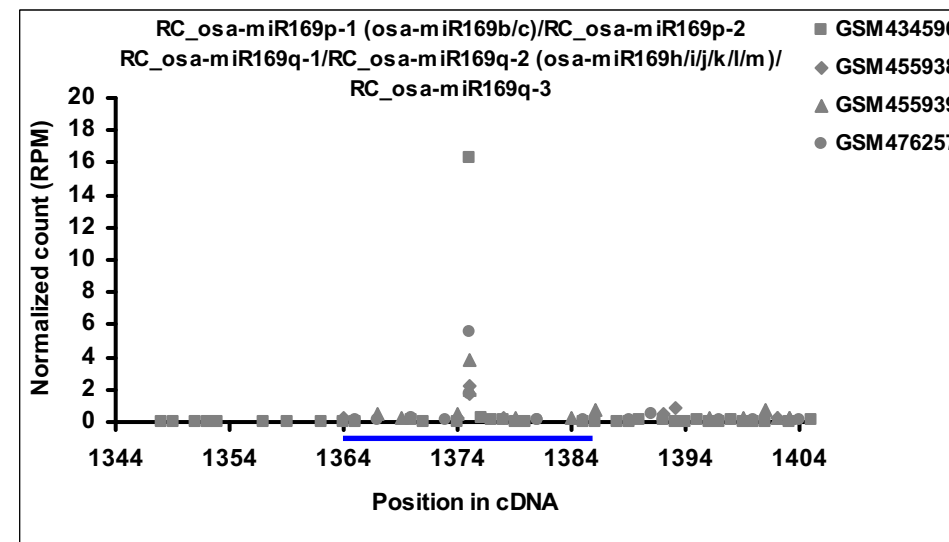

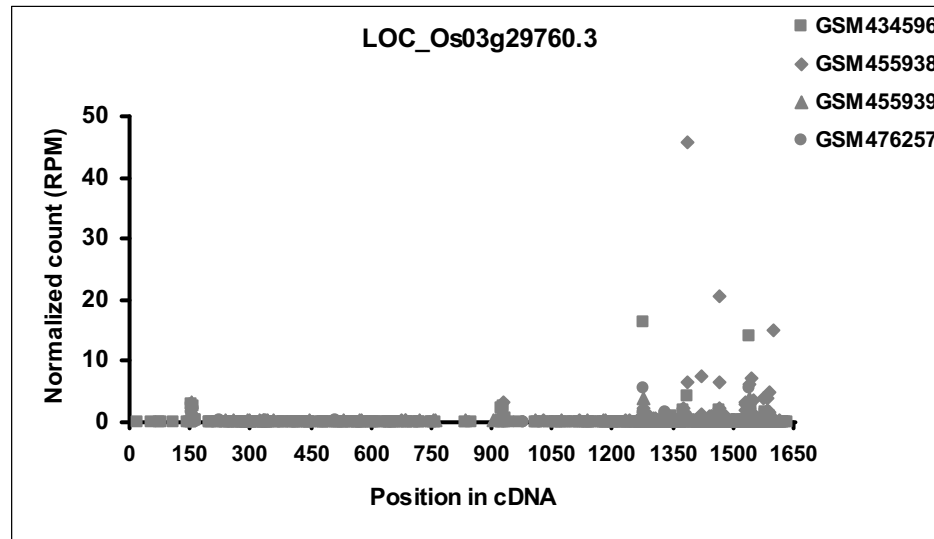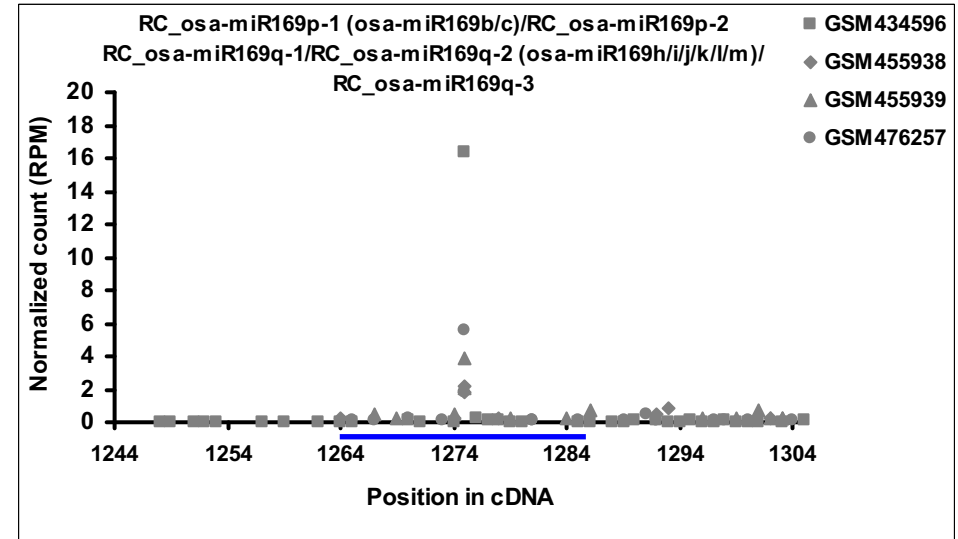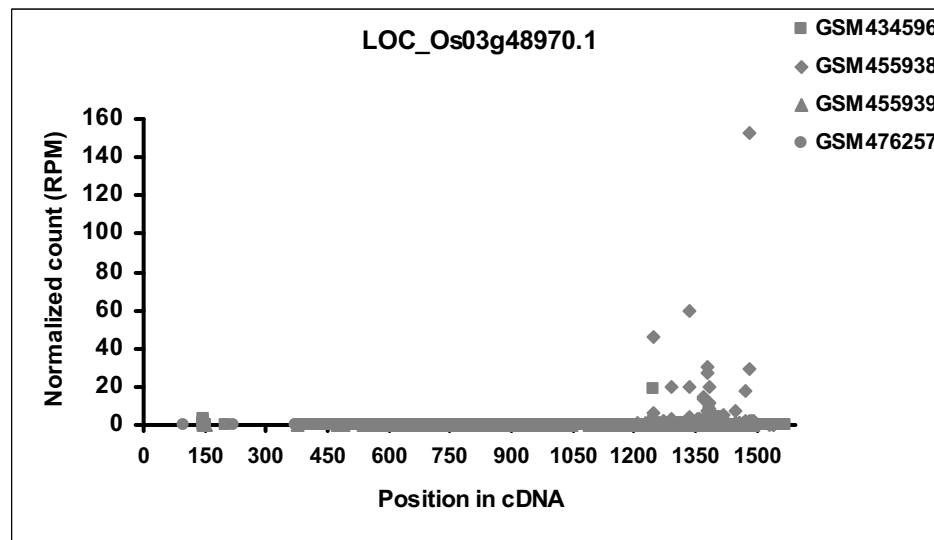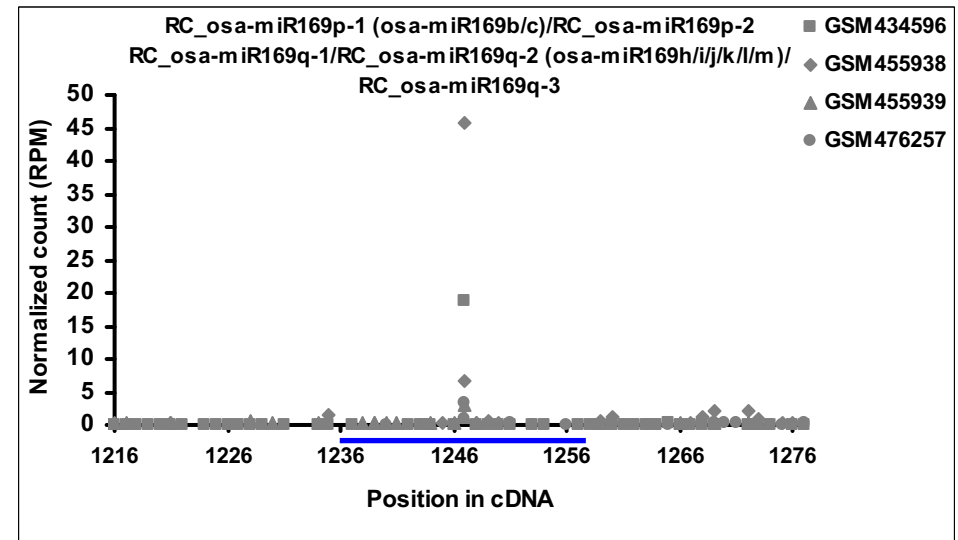

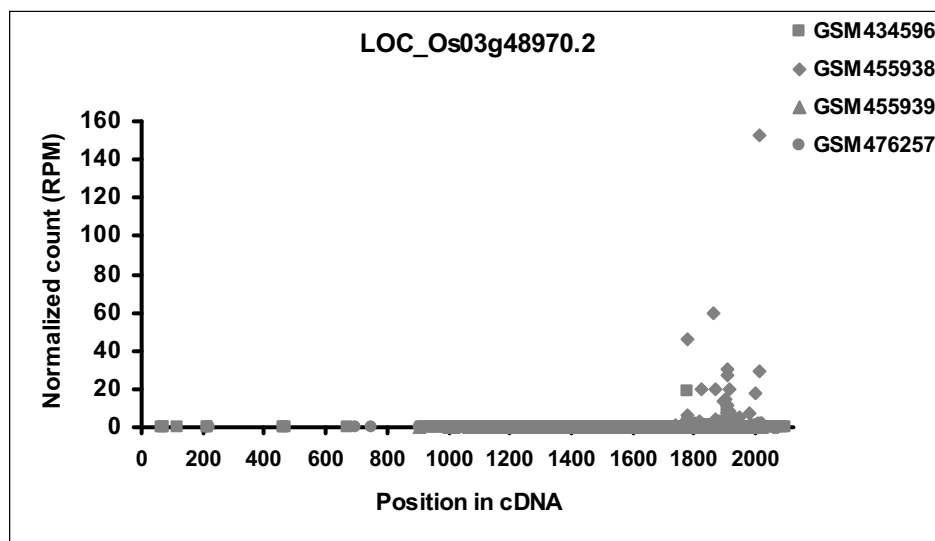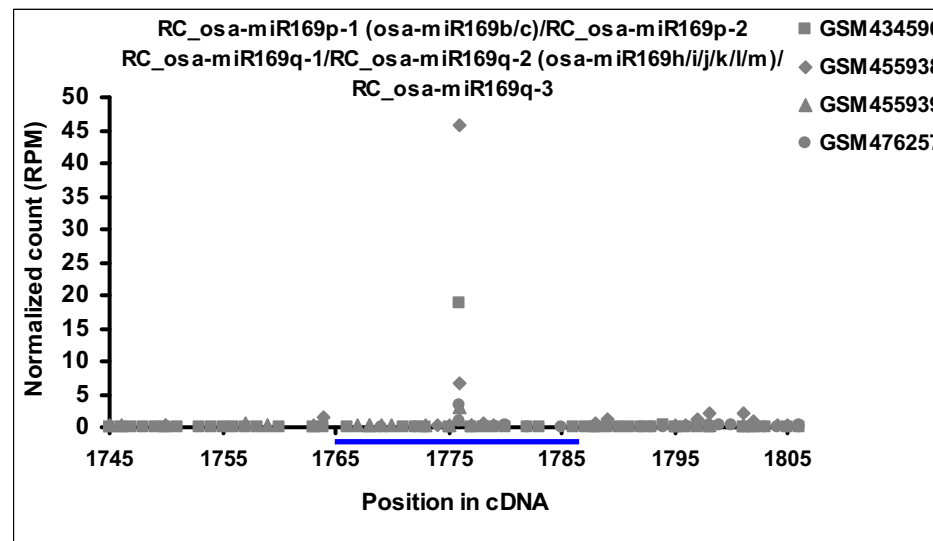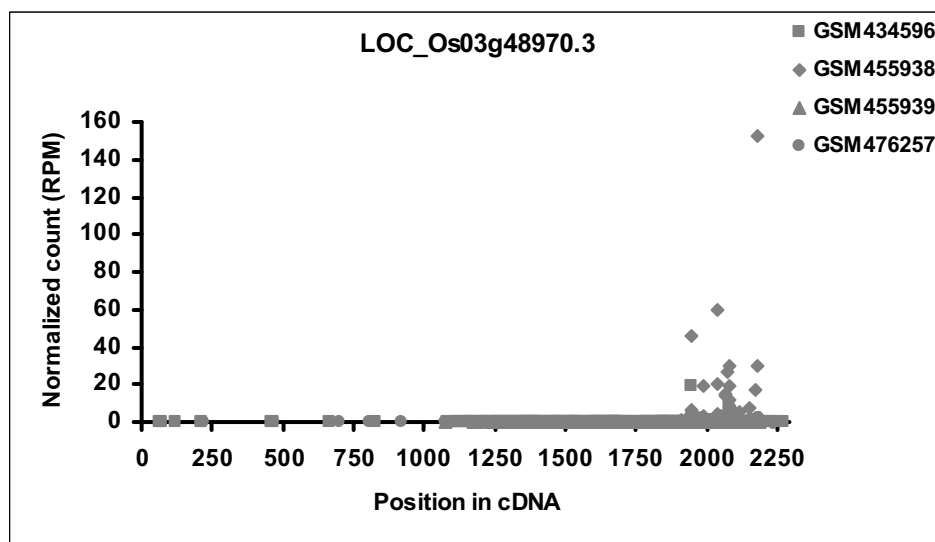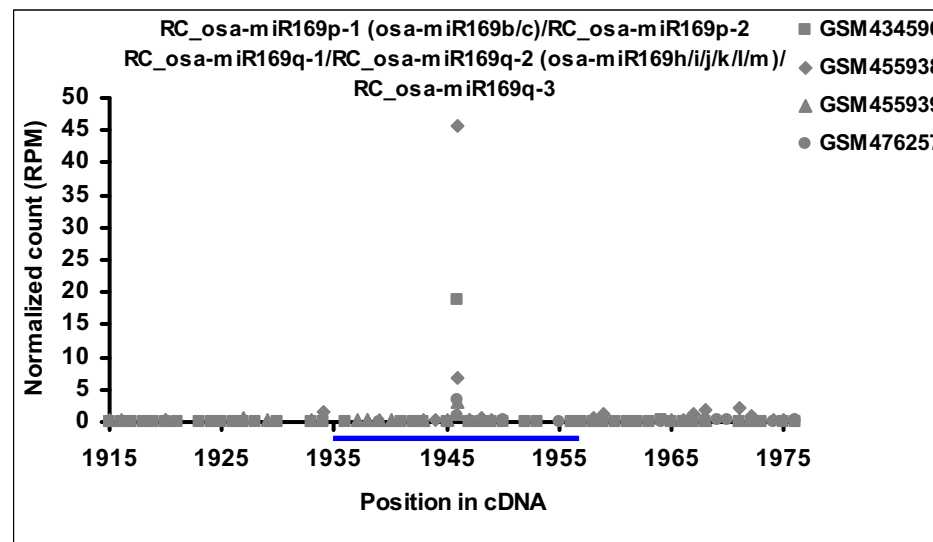

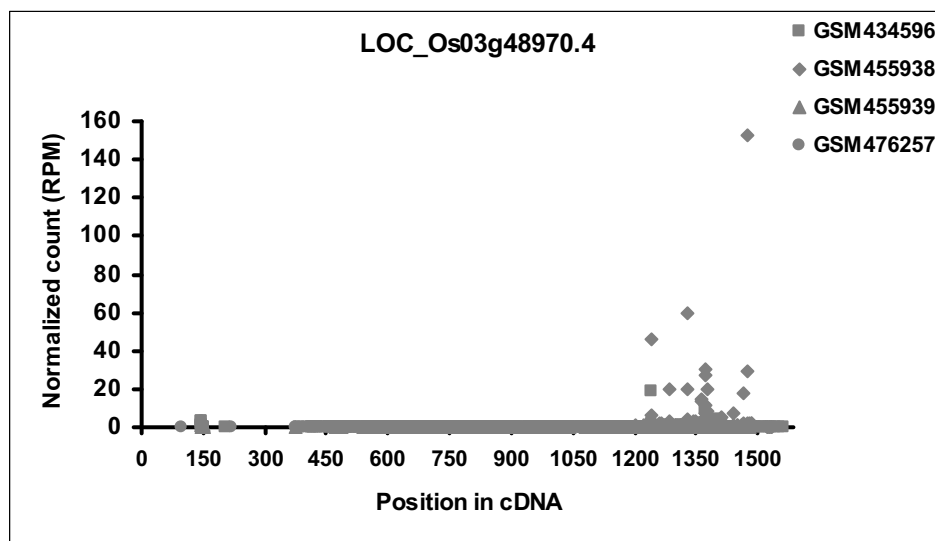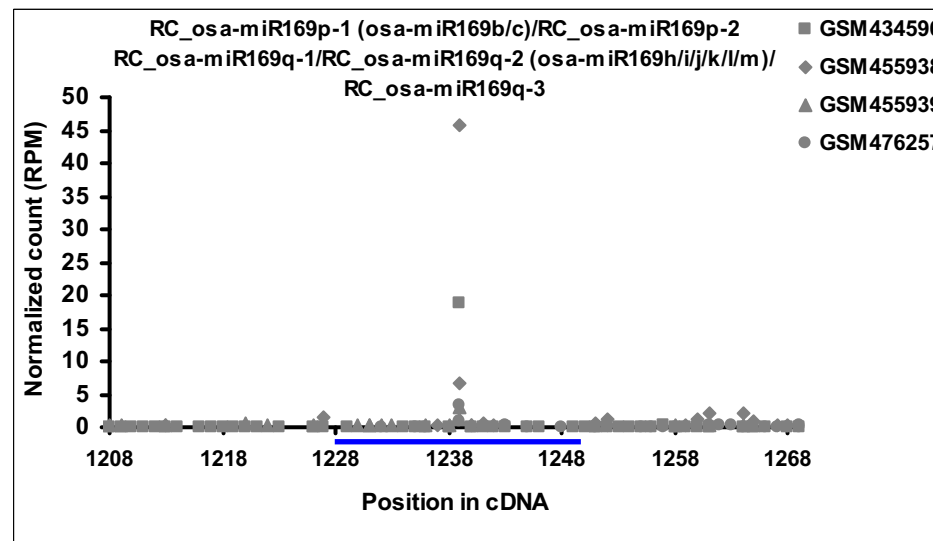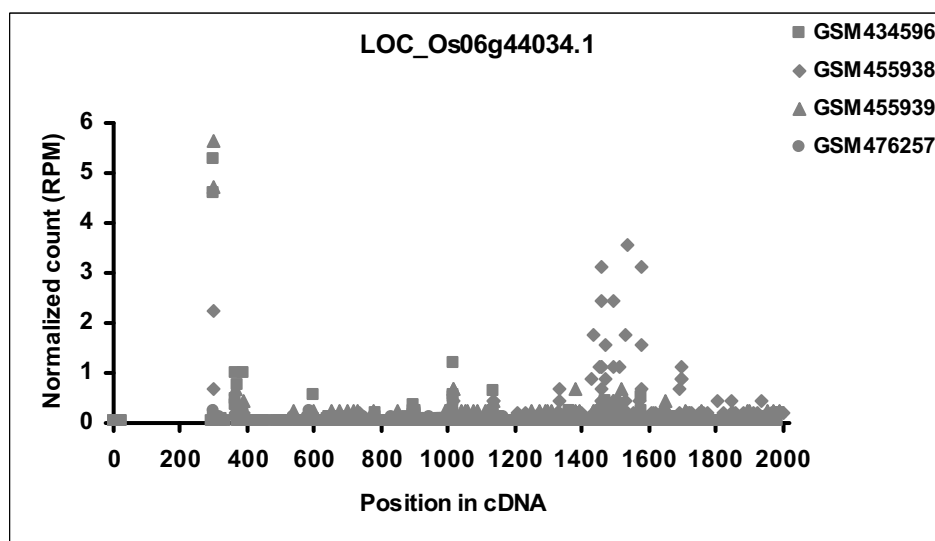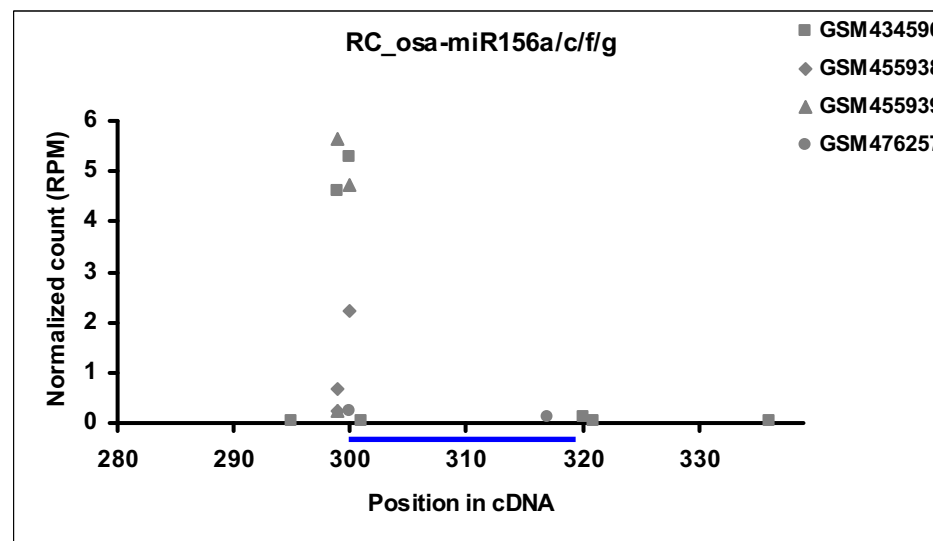

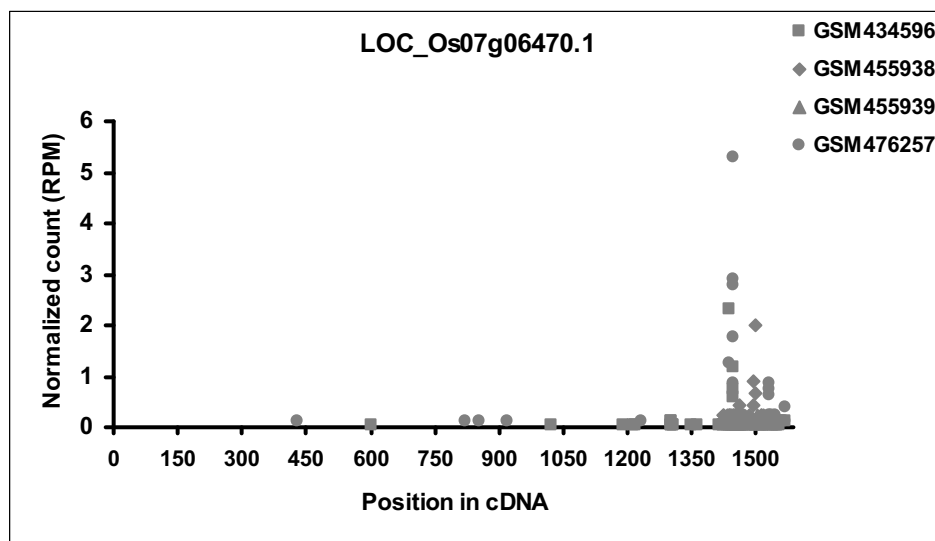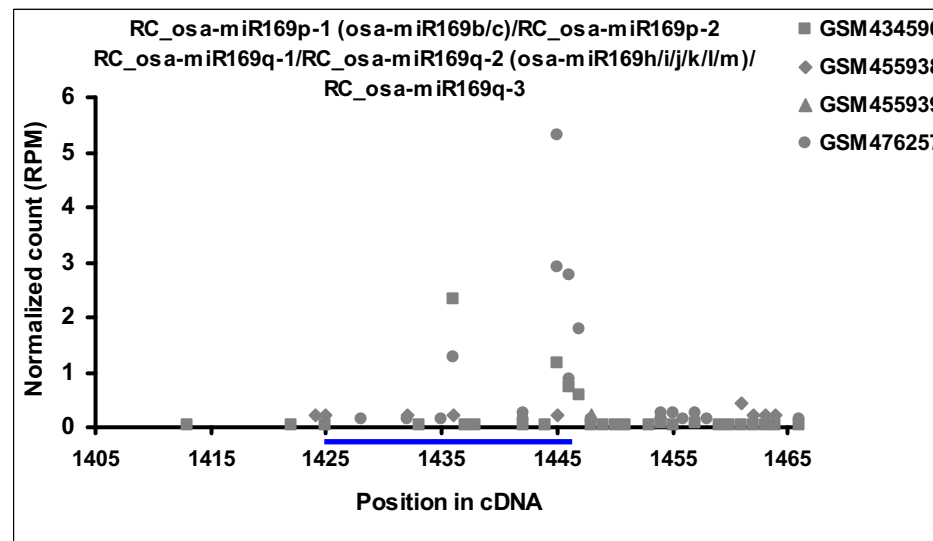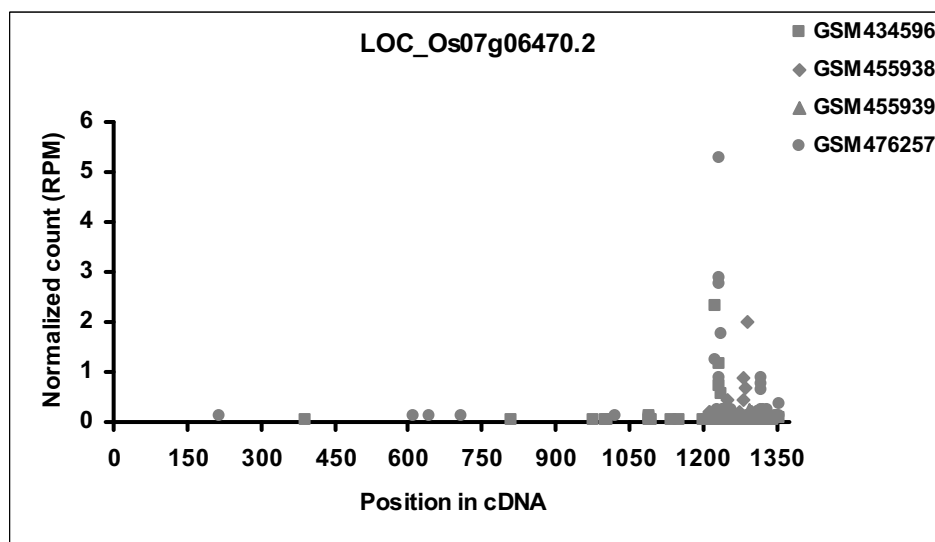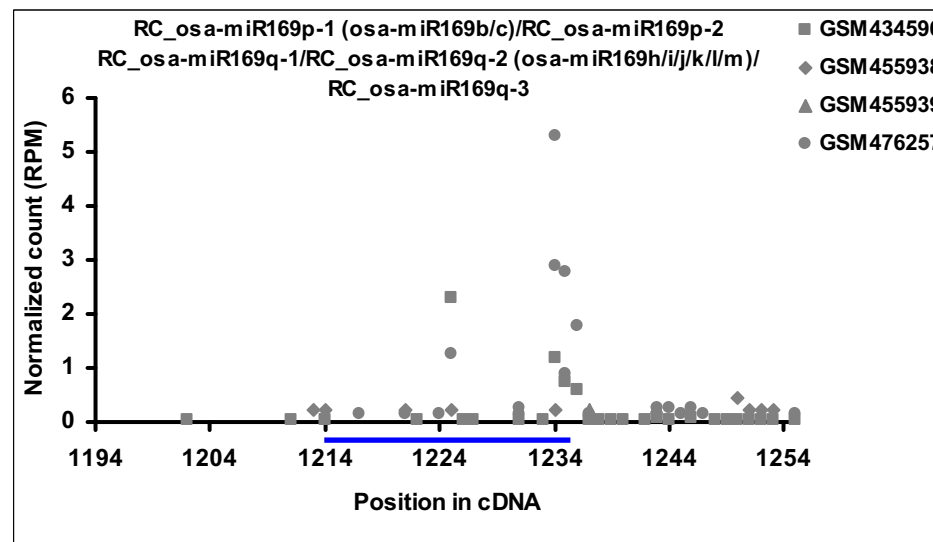

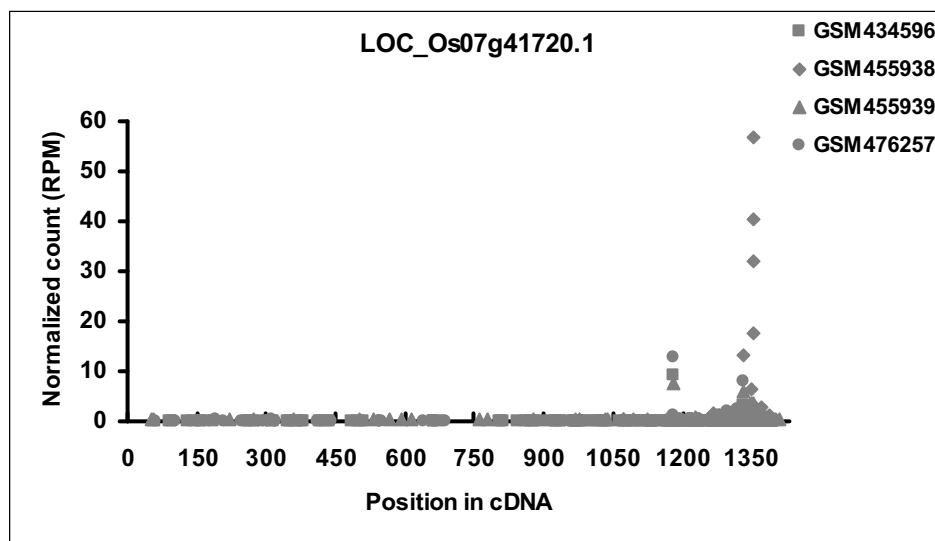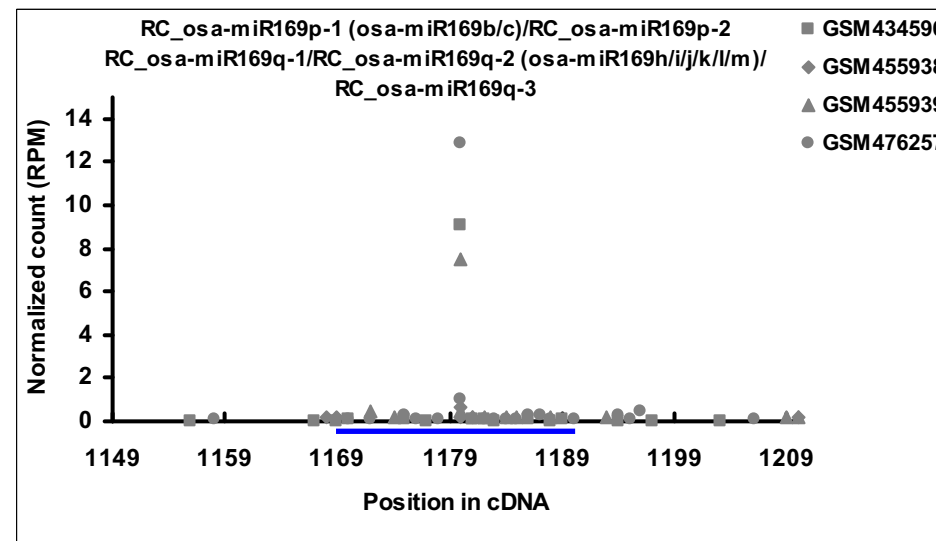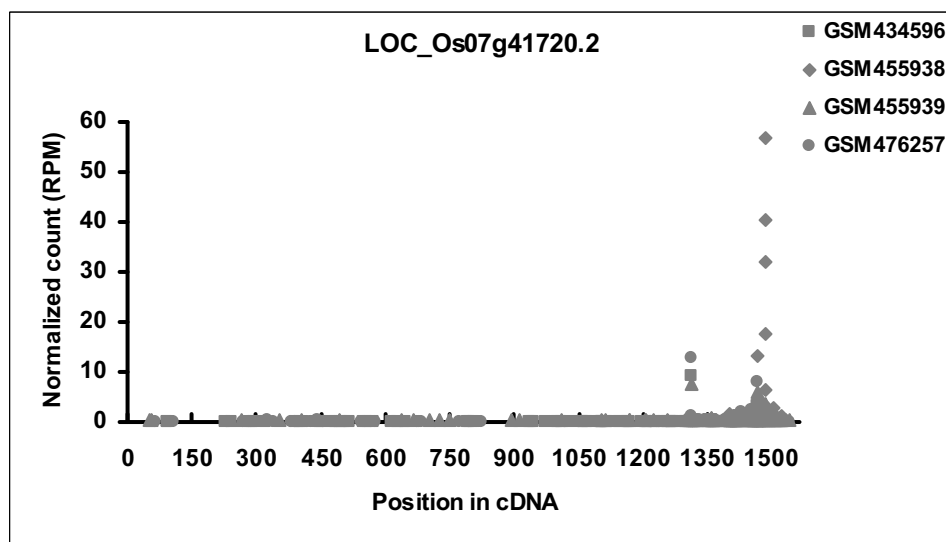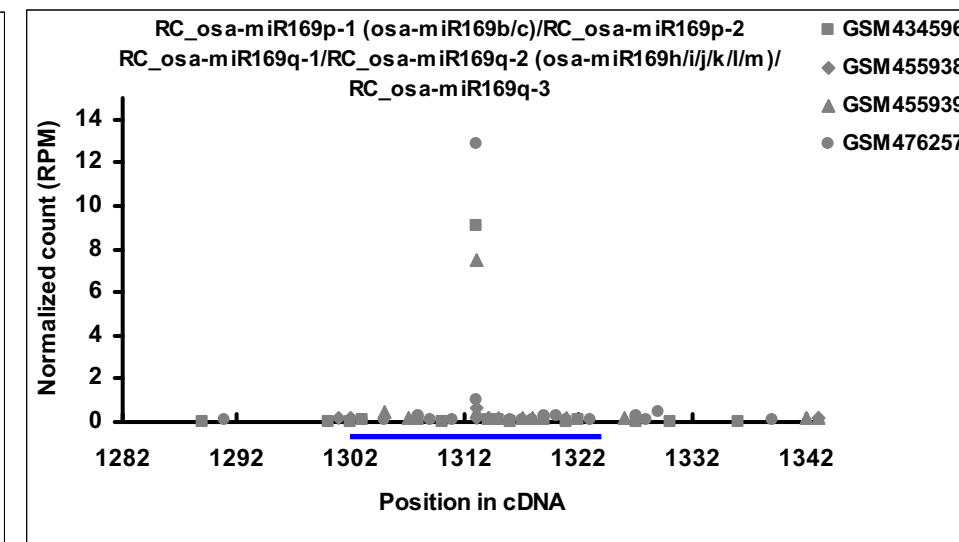

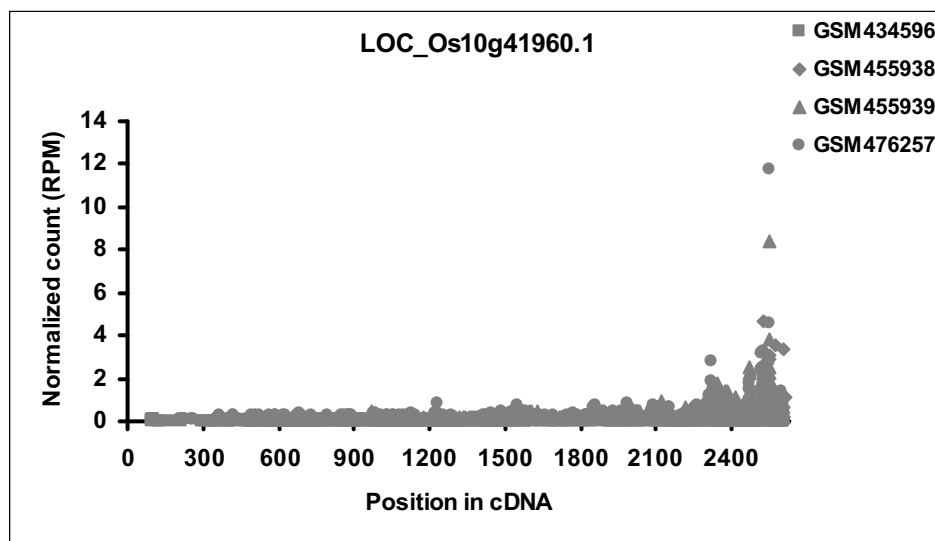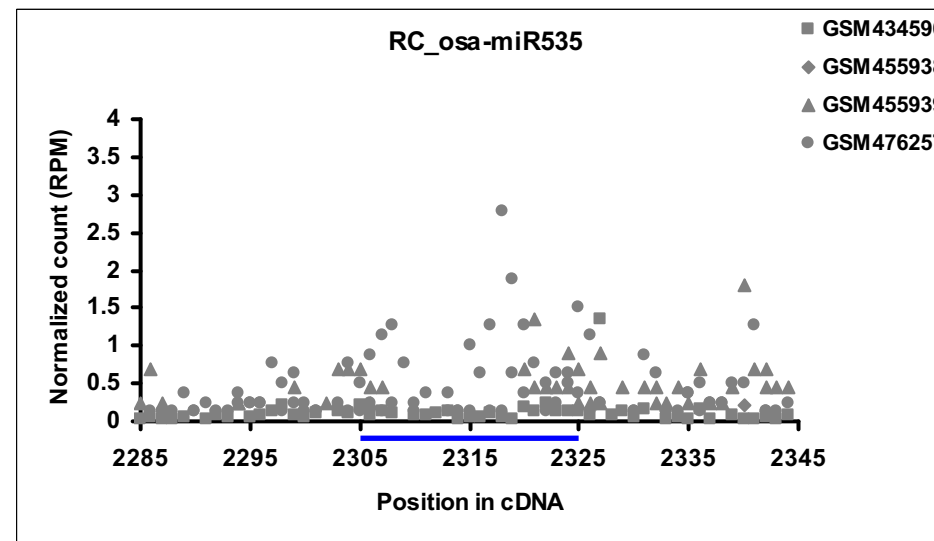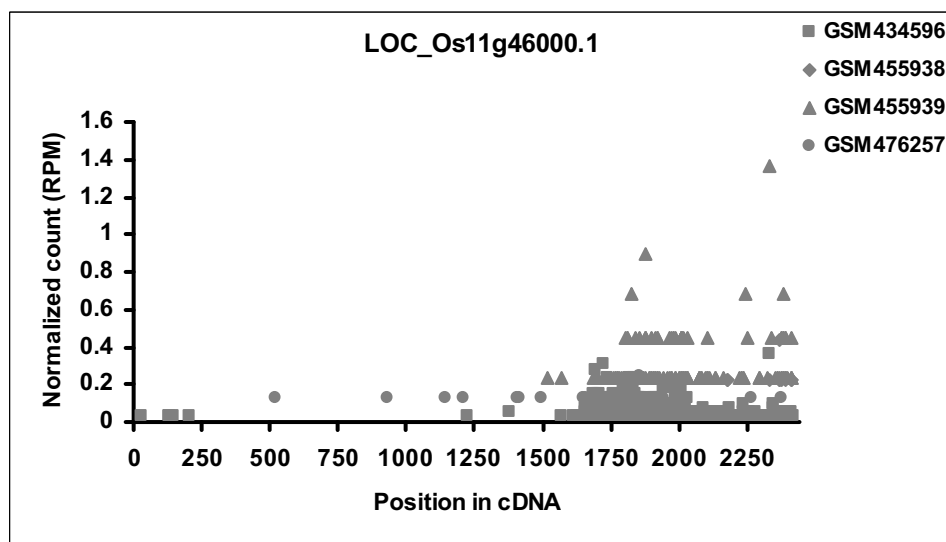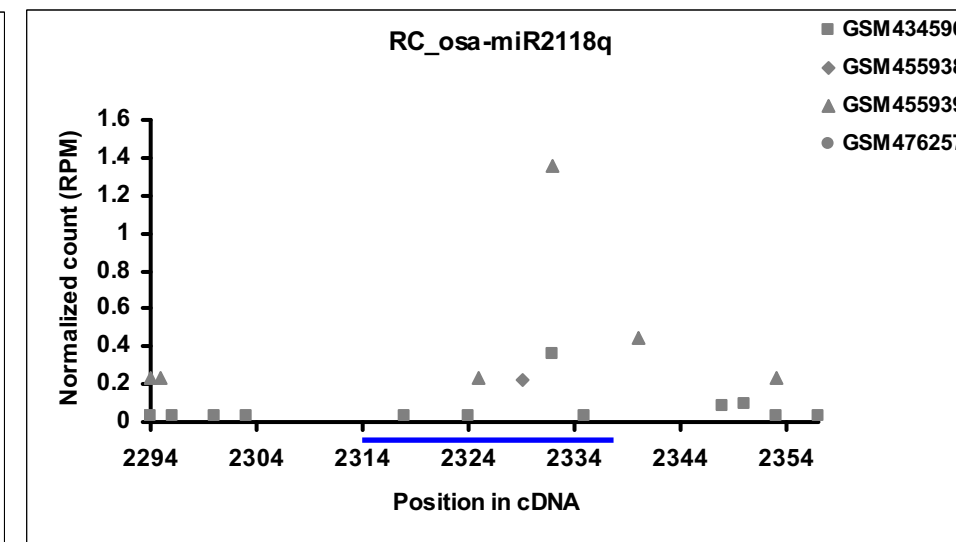

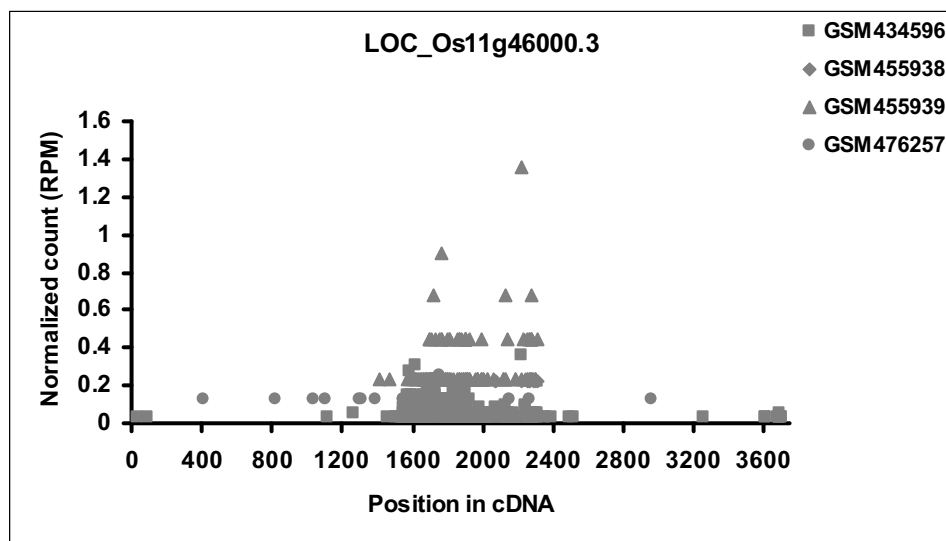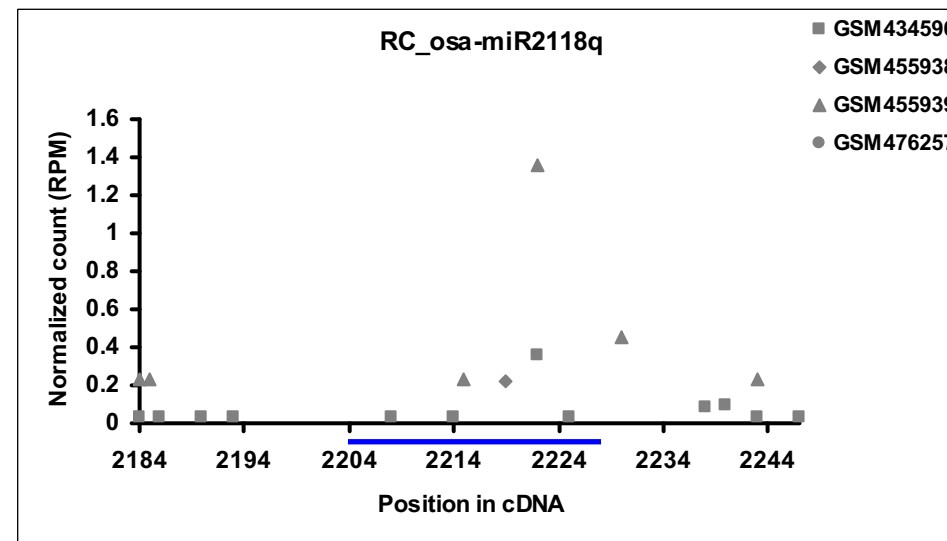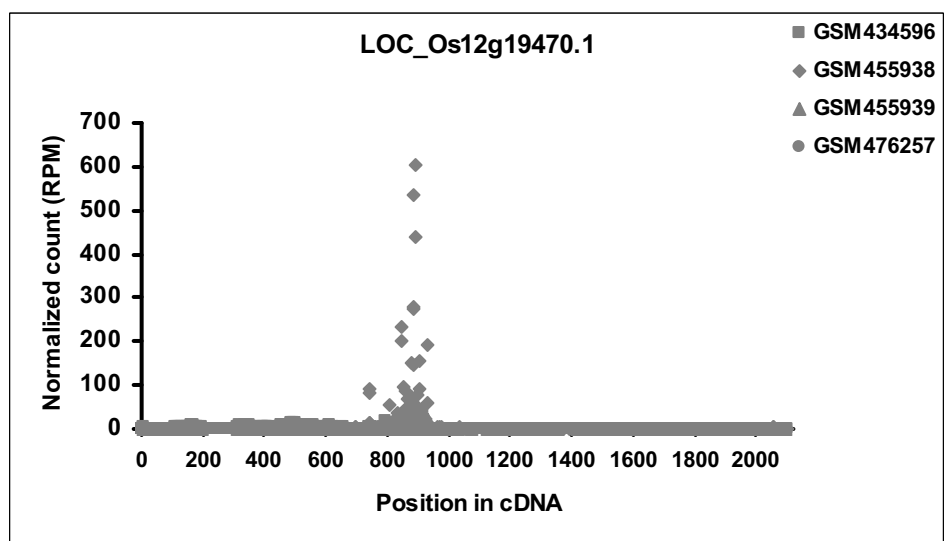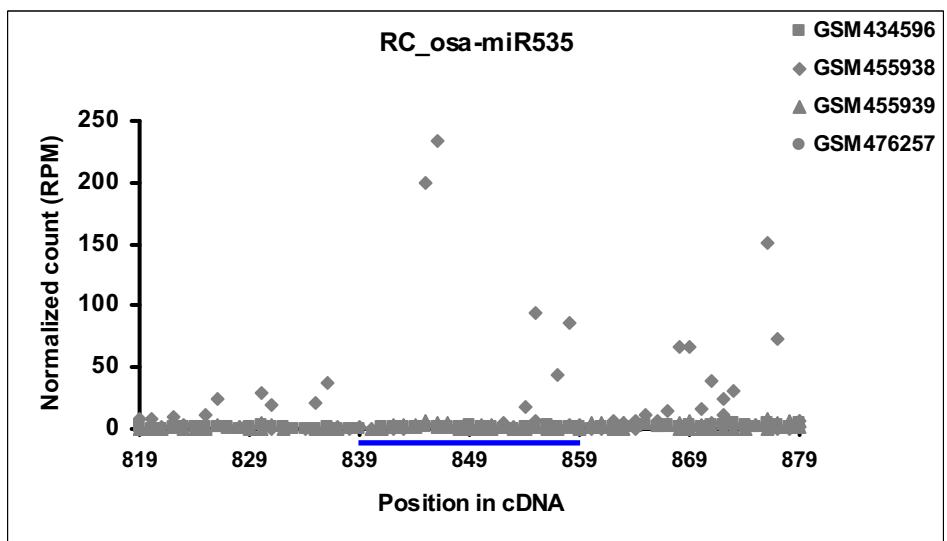

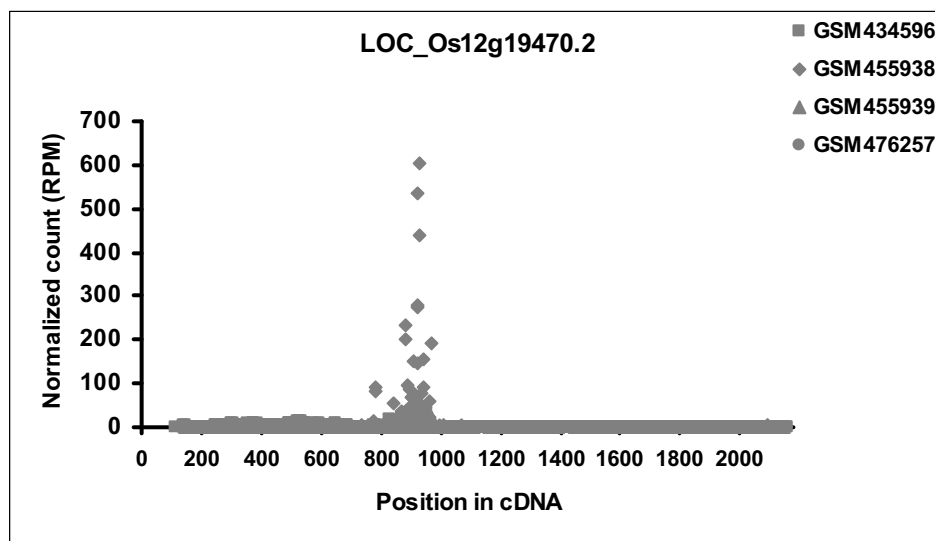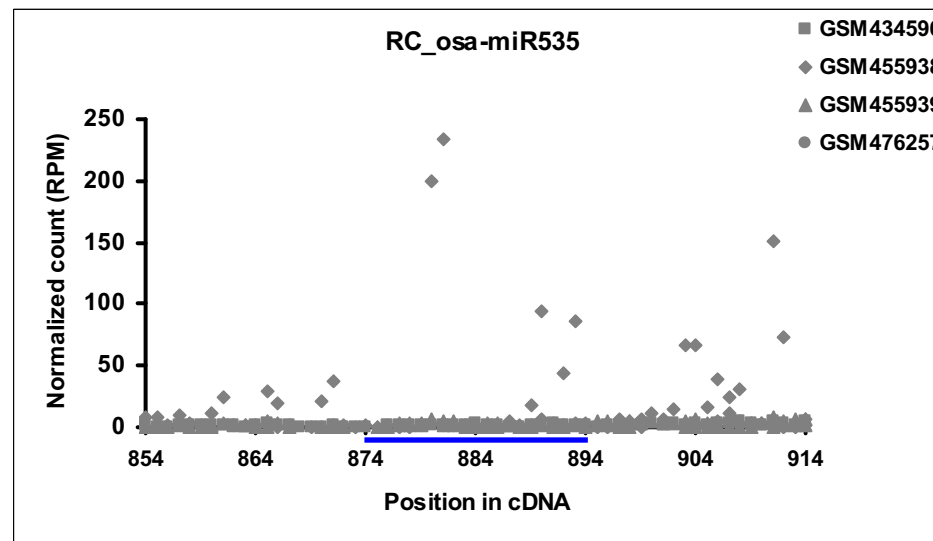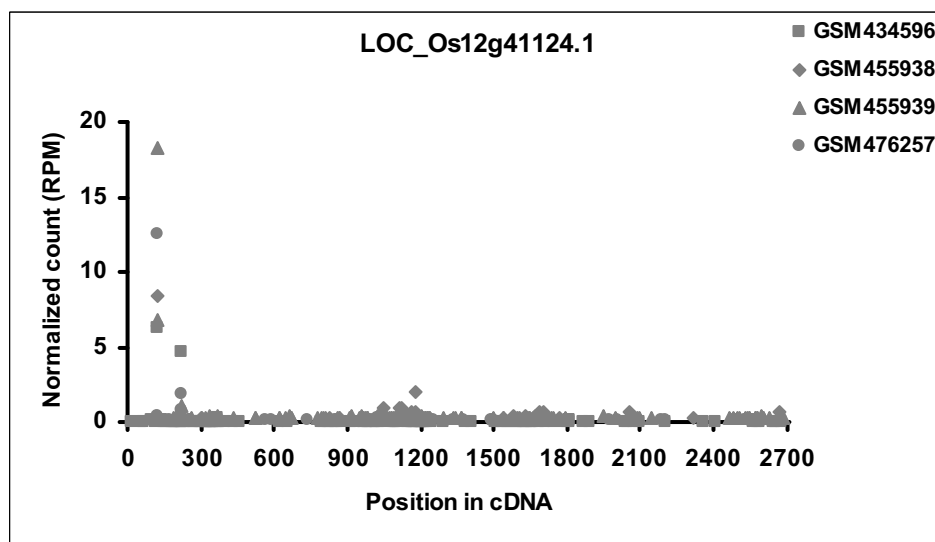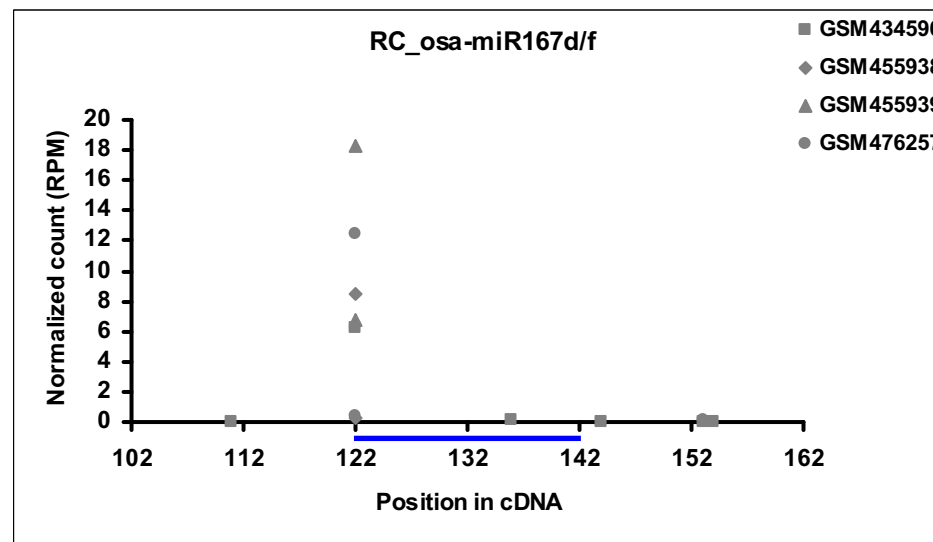

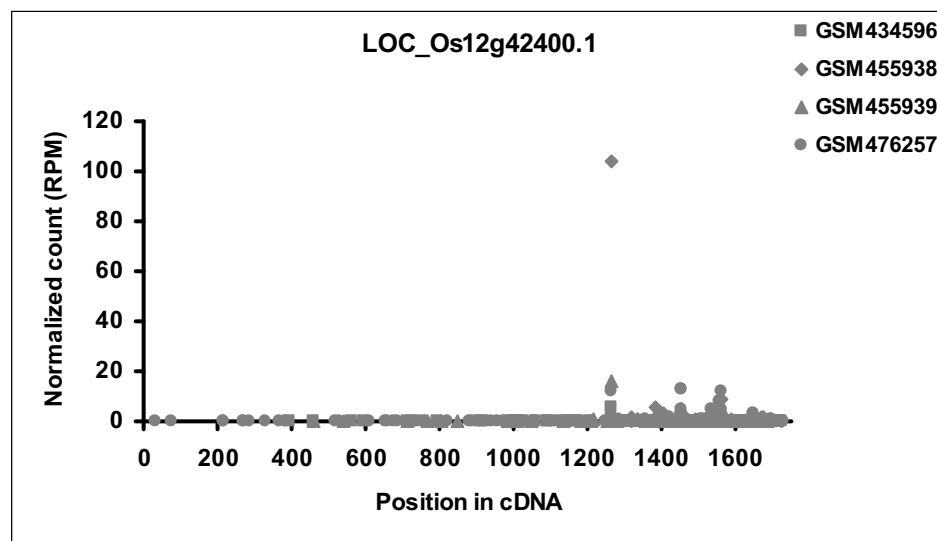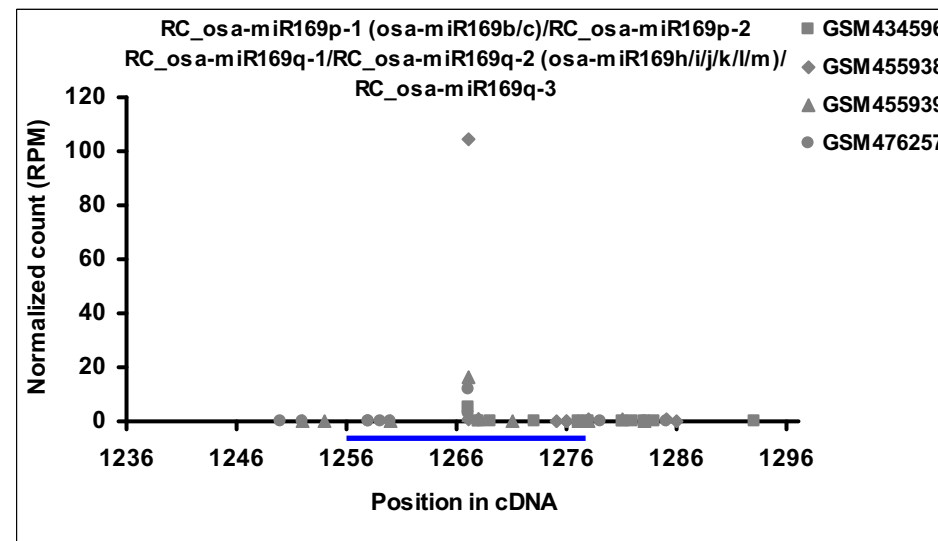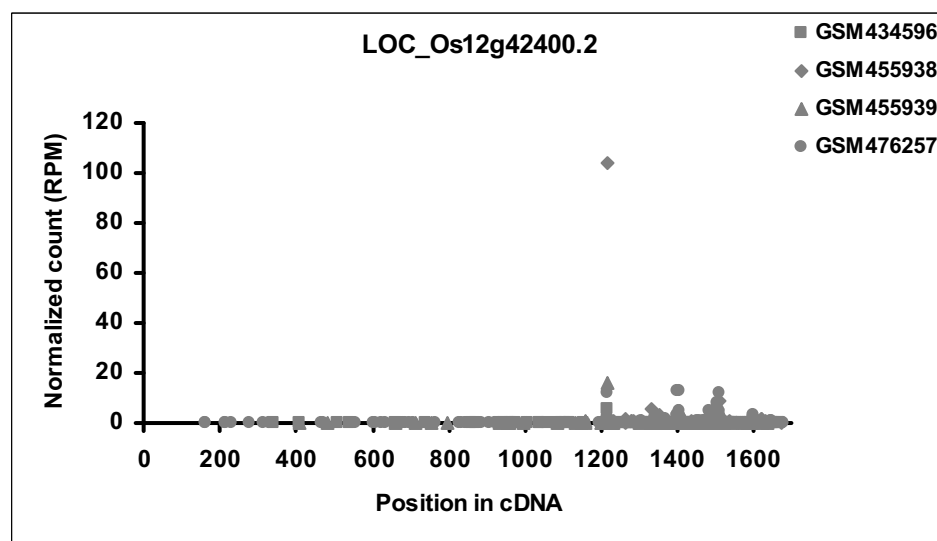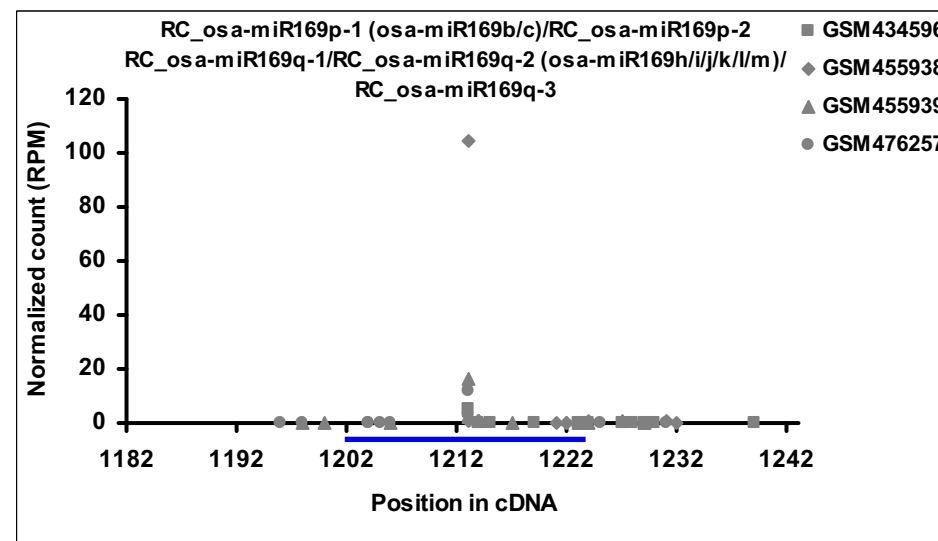

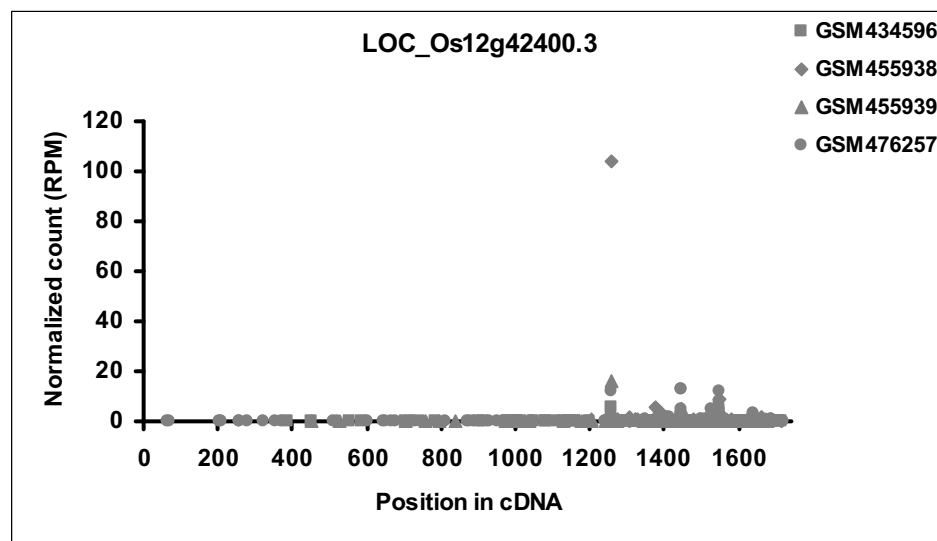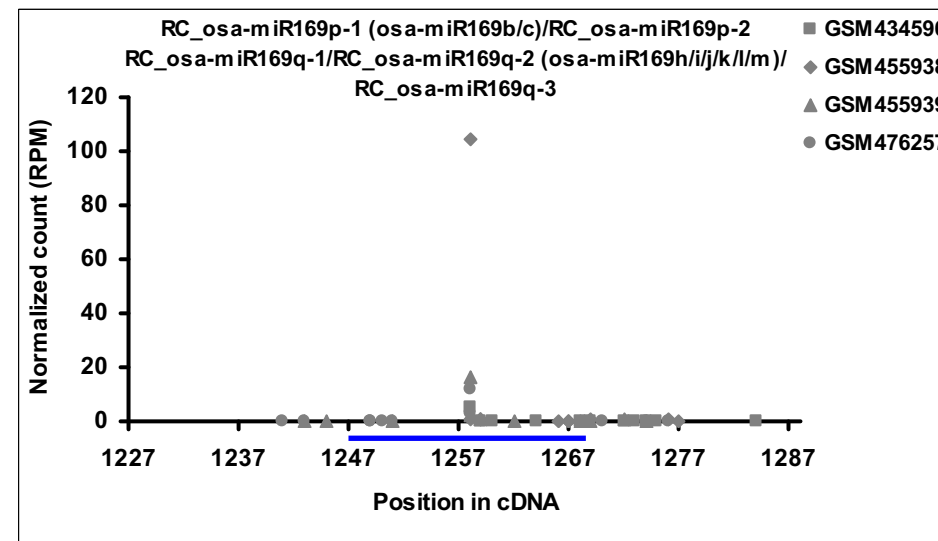

Supplement: Figure S3 — Degradome data-based validation of RC-miRNA-regulated target transcripts in rice. (PDF) [file pone.0046991.s003.pdf]

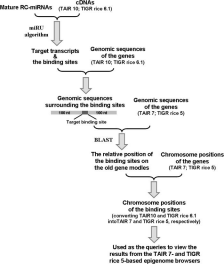

Supplement: Figure S4 — Schematic workflow of converting the genomic positions of target binding sites from TAIR 10 and TIGR rice 6.1 to TAIR 7 and TIGR rice 5 for DNA methylation profile queries in Arabidopsis and rice. (PDF) [file pone.0046991.s004.pdf]
