# Supplementary material for: Genome-Wide Identification of Reverse Complementary microRNA Genes in Plants
Source: PLoS One. 2012 Oct 23;7(10):e46991. doi: 10.1371/journal.pone.0046991 (PMC3479107; doi:10.1371/journal.pone.0046991)
Supplement: Figure S5 — RC-miRNA-guided DNA methylation surrounding their target recognition sites in Arabidopsis . (PDF) [file pone.0046991.s005.pdf]

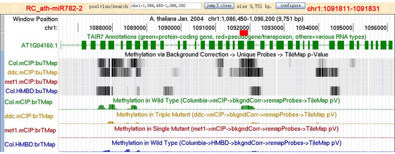
RC_ath-miR782-2
position/search
chr1:1,086,450-1,096,200
jump
clear
size 9,751 bp.
configure
chr1:1091811-1091831
Window Position
A. thaliana Jan. 2004 chr1:1,086,450-1,096,200 (9,751 bp)
chr1:
1088000
1089000
1090000
1091000
1092000
1093000
1094000
1095000
1096000
TAIR7 Annotations (green=protein-coding gene, red=pseudogene/transposon, others=various RNA types)
AT1G04160.1
Methylation via Background Correction -> Unique Probes -> TileMap p-Value
Col.mCIP:buTMap
ddc.mCIP:buTMap
met1.mCIP:buTMap
Col.HMBD:buTMap
Col.mCIP:brTMap
Methylation in Wild Type (Columbia->mCIP->bkgndCorr->remapProbes->TileMap pV)
ddc.mCIP:brTMap
Methylation in Triple Mutant (ddc->mCIP->bkgndCorr->remapProbes->TileMap pV)
met1.mCIP:brTMap
Methylation in Single Mutant (met1->mCIP->bkgndCorr->remapProbes->TileMap pV)
Col.HMBD:brTMap
Methylation in Wild Type (Columbia->HMBD->bkgndCorr->remapProbes->TileMap pV)

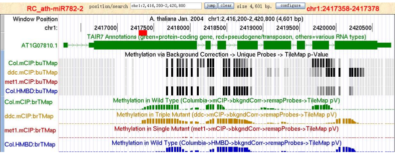

RC_ath-miR782-2
position/search chr1:2,416,200-2,420,800
jump
clear
size 4,601 bp.
configure
chr1:2417358-2417378
Window Position
A. thaliana Jan. 2004 chr1:2,416,200-2,420,800 (4,601 bp)
chr1:
2417000
2417500
2418000
2418500
2419000
2419500
2420000
2420500
TAIR7 Annotations (green=protein-coding gene, red=pseudogene/transposon, others=various RNA types)
AT1G07810.1
Methylation via Background Correction -> Unique Probes -> TileMap p-Value
Col.mCIP:buTMap
ddc.mCIP:buTMap
met1.mCIP:buTMap
Col.HMBD:buTMap
Col.mCIP:brTMap
Methylation in Wild Type (Columbia->mCIP->bkgndCorr->remapProbes->TileMap pV)
ddc.mCIP:brTMap
Methylation in Triple Mutant (ddc->mCIP->bkgndCorr->remapProbes->TileMap pV)
met1.mCIP:brTMap
Methylation in Single Mutant (met1->mCIP->bkgndCorr->remapProbes->TileMap pV)
Col.HMBD:brTMap
Methylation in Wild Type (Columbia->HMBD->bkgndCorr->remapProbes->TileMap pV)

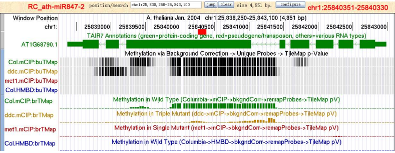
RC_ath-miR847-2
position/search
chr1:25,838,250-25,843,100
jump
clear
size 4,851 bp.
configure
chr1:25840351-25840330
Window Position
A. thaliana Jan. 2004 chr1:25,838,250-25,843,100 (4,851 bp)
chr1:
25839000
25839500
25840000
25840500
25841000
25841500
25842000
25842500
25843000
TAIR7 Annotations (green=protein-coding gene, red=pseudogene/transposon, others=various RNA types)
AT1G68790.1
Methylation via Background Correction -> Unique Probes -> TileMap p-Value
Col.mCIP:buTMap
ddc.mCIP:buTMap
met1.mCIP:buTMap
Col.HMBD:buTMap
Col.mCIP:brTMap
Methylation in Wild Type (Columbia->mCIP->bkgndCorr->remapProbes->TileMap pV)
ddc.mCIP:brTMap
Methylation in Triple Mutant (ddc->mCIP->bkgndCorr->remapProbes->TileMap pV)
met1.mCIP:brTMap
Methylation in Single Mutant (met1->mCIP->bkgndCorr->remapProbes->TileMap pV)
Col.HMBD:brTMap
Methylation in Wild Type (Columbia->HMBD->bkgndCorr->remapProbes->TileMap pV)

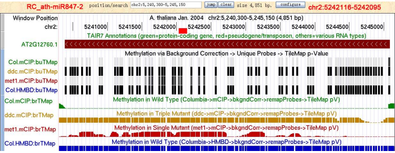
RC_ath-miR847-2
position/search chr2:5,240,300-5,245,150
jump
clear
size 4,851 bp.
configure
chr2:5242116-5242095
Window Position
A. thaliana Jan. 2004 chr2:5,240,300-5,245,150 (4,851 bp)
chr2:
5241000
5241500
5242000
5242500
5243000
5243500
5244000
5244500
5245000
TAIR7 Annotations (green=protein-coding gene, red=pseudogene/transposon, others=various RNA types)
AT2G12760.1
Methylation via Background Correction -> Unique Probes -> TileMap p-Value
Col.mCIP:buTMap
ddc.mCIP:buTMap
met1.mCIP:buTMap
Col.HMBD:buTMap
Col.mCIP:brTMap
Methylation in Wild Type (Columbia->mCIP->bkgndCorr->remapProbes->TileMap pV)
ddc.mCIP:brTMap
Methylation in Triple Mutant (ddc->mCIP->bkgndCorr->remapProbes->TileMap pV)
met1.mCIP:brTMap
Methylation in Single Mutant (met1->mCIP->bkgndCorr->remapProbes->TileMap pV)
Col.HMBD:brTMap
Methylation in Wild Type (Columbia->HMBD->bkgndCorr->remapProbes->TileMap pV)

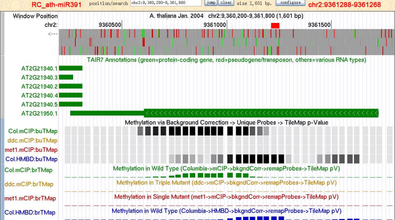

RC_ath-miR391
position/search chr2:9,360,200-9,361,800
jump
clear
size 1,601 bp.
configure
chr2:9361288-9361268
Window Position
A. thaliana Jan. 2004 chr2:9,360,200-9,361,800 (1,601 bp)
chr2:
9360500
9361000
9361500
TAIR7 Annotations (green=protein-coding gene, red=pseudogene/transposon, others=various RNA types)
AT2G21940.1
AT2G21940.3
AT2G21940.2
AT2G21940.4
AT2G21940.5
AT2G21950.1
Methylation via Background Correction -> Unique Probes -> TileMap p-Value
Col.mCIP:buTMap
ddc.mCIP:buTMap
met1.mCIP:buTMap
Col.HMBD:buTMap
Col.mCIP:brTMap
Methylation in Wild Type (Columbia->mCIP->bkgndCorr->remapProbes->TileMap pV)
ddc.mCIP:brTMap
Methylation in Triple Mutant (ddc->mCIP->bkgndCorr->remapProbes->TileMap pV)
met1.mCIP:brTMap
Methylation in Single Mutant (met1->mCIP->bkgndCorr->remapProbes->TileMap pV)
Col.HMBD:brTMap
Methylation in Wild Type (Columbia->HMBD->bkgndCorr->remapProbes->TileMap pV)

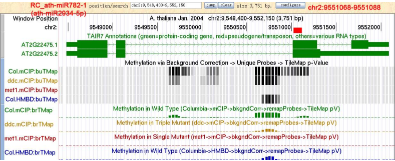

RC_ath-miR782-1
(ath-miR2934-5p)
position/search
chr2:9,548,400-9,552,150
jump
clear
size 3,751 bp.
configure
chr2:9551068-9551088
Window Position
A. thaliana Jan. 2004 chr2:9,548,400-9,552,150 (3,751 bp)
chr2:
9549000
9549500
9550000
9550500
9551000
9551500
9552000
TAIR7 Annotations (green=protein-coding gene, red=pseudogene/transposon, others=various RNA types)
AT2G22475.1
AT2G22475.2
Methylation via Background Correction -> Unique Probes -> TileMap p-Value
Col.mCIP:buTMap
ddc.mCIP:buTMap
met1.mCIP:buTMap
Col.HMBD:buTMap
Col.mCIP:brTMap
Methylation in Wild Type (Columbia->mCIP->bkgndCorr->remapProbes->TileMap pV)
ddc.mCIP:brTMap
Methylation in Triple Mutant (ddc->mCIP->bkgndCorr->remapProbes->TileMap pV)
met1.mCIP:brTMap
Methylation in Single Mutant (met1->mCIP->bkgndCorr->remapProbes->TileMap pV)
Col.HMBD:brTMap
Methylation in Wild Type (Columbia->HMBD->bkgndCorr->remapProbes->TileMap pV)

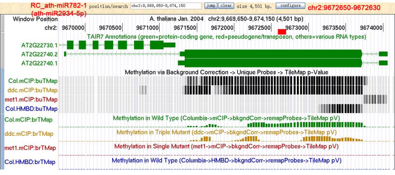
RC_ath-miR782-1
(ath-miR2934-5p)
position/search
chr2:9,669,650-9,674,150
jump
clear
size 4,501 bp.
configure
chr2:9672650-9672630
Window Position
A. thaliana Jan. 2004 chr2:9,669,650-9,674,150 (4,501 bp)
chr2:
9670000
9670500
9671000
9671500
9672000
9672500
9673000
9673500
9674000
TAIR7 Annotations (green=protein-coding gene, red=pseudogene/transposon, others=various RNA types)
AT2G22730.1
AT2G22740.2
AT2G22740.1
Methylation via Background Correction -> Unique Probes -> TileMap p-Value
Col.mCIP:buTMap
ddc.mCIP:buTMap
met1.mCIP:buTMap
Col.HMBD:buTMap
Col.mCIP:brTMap
Methylation in Wild Type (Columbia->mCIP->bkgndCorr->remapProbes->TileMap pV)
ddc.mCIP:brTMap
Methylation in Triple Mutant (ddc->mCIP->bkgndCorr->remapProbes->TileMap pV)
met1.mCIP:brTMap
Methylation in Single Mutant (met1->mCIP->bkgndCorr->remapProbes->TileMap pV)
Col.HMBD:brTMap
Methylation in Wild Type (Columbia->HMBD->bkgndCorr->remapProbes->TileMap pV)

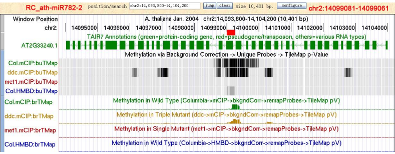

RC_ath-miR782-2
position/search chr2:14,093,800-14,104,200
jump
clear
size 10,401 bp.
configure
chr2:14099081-14099061
Window Position
A. thaliana Jan. 2004 chr2:14,093,800-14,104,200 (10,401 bp)
chr2:
14095000
14096000
14097000
14098000
14099000
14100000
14101000
14102000
14103000
14104000
TAIR7 Annotations (green=protein-coding gene, red=pseudogene/transposon, others=various RNA types)
AT2G33240.1
Methylation via Background Correction -> Unique Probes -> TileMap p-Value
Col.mCIP:buTMap
ddc.mCIP:buTMap
met1.mCIP:buTMap
Col.HMBD:buTMap
Col.mCIP:brTMap
Methylation in Wild Type (Columbia->mCIP->bkgndCorr->remapProbes->TileMap pV)
ddc.mCIP:brTMap
Methylation in Triple Mutant (ddc->mCIP->bkgndCorr->remapProbes->TileMap pV)
met1.mCIP:brTMap
Methylation in Single Mutant (met1->mCIP->bkgndCorr->remapProbes->TileMap pV)
Col.HMBD:brTMap
Methylation in Wild Type (Columbia->HMBD->bkgndCorr->remapProbes->TileMap pV)

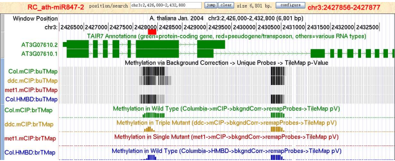

RC_ath-miR847-2
position/search
chr3:2,426,000-2,432,800
jump
clear
size 6,801 bp.
configure
chr3:2427856-2427877
Window Position
A. thaliana Jan. 2004 chr3:2,426,000-2,432,800 (6,801 bp)
chr3: 2426500 2427000 2427500 2428000 2428500 2429000 2429500 2430000 2430500 2431000 2431500 2432000 2432500
TAIR7 Annotations (green=protein-coding gene, red=pseudogene/transposon, others=various RNA types)
AT3G07610.2
AT3G07610.1
Methylation via Background Correction -> Unique Probes -> TileMap p-Value
Col.mCIP:buTMap
ddc.mCIP:buTMap
met1.mCIP:buTMap
Col.HMBD:buTMap
Col.mCIP:brTMap
Methylation in Wild Type (Columbia->mCIP->bkgndCorr->remapProbes->TileMap pV)
ddc.mCIP:brTMap
Methylation in Triple Mutant (ddc->mCIP->bkgndCorr->remapProbes->TileMap pV)
met1.mCIP:brTMap
Methylation in Single Mutant (met1->mCIP->bkgndCorr->remapProbes->TileMap pV)
Col.HMBD:brTMap
Methylation in Wild Type (Columbia->HMBD->bkgndCorr->remapProbes->TileMap pV)

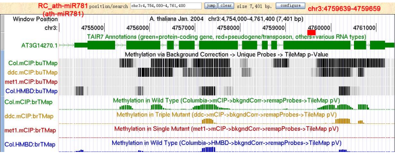

RC_ath-miR781
(ath-miR781)
position/search chr3:4,754,000-4,761,400
jump
clear
size 7,401 bp.
configure
chr3:4759639-4759659
Window Position
A. thaliana Jan. 2004 chr3:4,754,000-4,761,400 (7,401 bp)
chr3:
4755000
4756000
4757000
4758000
4759000
4760000
4761000
TAIR7 Annotations (green=protein-coding gene, red=pseudogene/transposon, others=various RNA types)
AT3G14270.1
Methylation via Background Correction -> Unique Probes -> TileMap p-Value
Col.mCIP:buTMap
ddc.mCIP:buTMap
met1.mCIP:buTMap
Col.HMBD:buTMap
Col.mCIP:brTMap
Methylation in Wild Type (Columbia->mCIP->bkgndCorr->remapProbes->TileMap pV)
ddc.mCIP:brTMap
Methylation in Triple Mutant (ddc->mCIP->bkgndCorr->remapProbes->TileMap pV)
met1.mCIP:brTMap
Methylation in Single Mutant (met1->mCIP->bkgndCorr->remapProbes->TileMap pV)
Col.HMBD:brTMap
Methylation in Wild Type (Columbia->HMBD->bkgndCorr->remapProbes->TileMap pV)

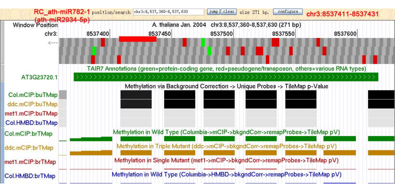

RC_ath-miR782-1
(ath-miR2934-5p)
position/search chr3:8,537,360-8,537,630
jump
clear
size 271 bp.
configure
chr3:8537411-8537431
Window Position
A. thaliana Jan. 2004 chr3:8,537,360-8,537,630 (271 bp)
chr3:
8537400
8537450
8537500
8537550
8537600
TAIR7 Annotations (green=protein-coding gene, red=pseudogene/transposon, others=various RNA types)
AT3G23720.1
Methylation via Background Correction -> Unique Probes -> TileMap p-Value
Col.mCIP:buTMap
ddc.mCIP:buTMap
met1.mCIP:buTMap
Col.HMBD:buTMap
Col.mCIP:brTMap
Methylation in Wild Type (Columbia->mCIP->bkgndCorr->remapProbes->TileMap pV)
ddc.mCIP:brTMap
Methylation in Triple Mutant (ddc->mCIP->bkgndCorr->remapProbes->TileMap pV)
met1.mCIP:brTMap
Methylation in Single Mutant (met1->mCIP->bkgndCorr->remapProbes->TileMap pV)
Col.HMBD:brTMap
Methylation in Wild Type (Columbia->HMBD->bkgndCorr->remapProbes->TileMap pV)

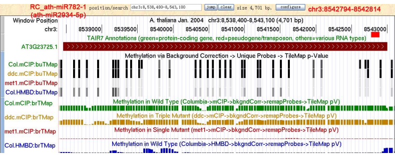
RC_ath-miR782-1
(ath-miR2934-5p)
position/search chr3:8,538,400-8,543,100 jump clear size 4,701 bp. configure
chr3:8542794-8542814
Window Position
A. thaliana Jan. 2004 chr3:8,538,400-8,543,100 (4,701 bp)
chr3:
8539000
8539500
8540000
8540500
8541000
8541500
8542000
8542500
8543000
TAIR7 Annotations (green=protein-coding gene, red=pseudogene/transposon, others=various RNA types)
AT3G23725.1
Methylation via Background Correction -> Unique Probes -> TileMap p-Value
Col.mCIP:buTMap
ddc.mCIP:buTMap
met1.mCIP:buTMap
Col.HMBD:buTMap
Col.mCIP:brTMap
Methylation in Wild Type (Columbia->mCIP->bkgndCorr->remapProbes->TileMap pV)
ddc.mCIP:brTMap
Methylation in Triple Mutant (ddc->mCIP->bkgndCorr->remapProbes->TileMap pV)
met1.mCIP:brTMap
Methylation in Single Mutant (met1->mCIP->bkgndCorr->remapProbes->TileMap pV)
Col.HMBD:brTMap
Methylation in Wild Type (Columbia->HMBD->bkgndCorr->remapProbes->TileMap pV)

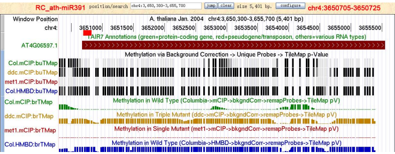

RC_ath-miR391
position/search
chr4:3,650,300-3,655,700
jump
clear
size 5,401 bp.
configure
chr4:3650705-3650725
Window Position
A. thaliana Jan. 2004 chr4:3,650,300-3,655,700 (5,401 bp)
chr4:
3651000
3651500
3652000
3652500
3653000
3653500
3654000
3654500
3655000
3655500
TAIR7 Annotations (green=protein-coding gene, red=pseudogene/transposon, others=various RNA types)
AT4G06597.1
Methylation via Background Correction -> Unique Probes -> TileMap p-Value
Col.mCIP:buTMap
ddc.mCIP:buTMap
met1.mCIP:buTMap
Col.HMBD:buTMap
Col.mCIP:brTMap
Methylation in Wild Type (Columbia->mCIP->bkgndCorr->remapProbes->TileMap pV)
ddc.mCIP:brTMap
Methylation in Triple Mutant (ddc->mCIP->bkgndCorr->remapProbes->TileMap pV)
met1.mCIP:brTMap
Methylation in Single Mutant (met1->mCIP->bkgndCorr->remapProbes->TileMap pV)
Col.HMBD:brTMap
Methylation in Wild Type (Columbia->HMBD->bkgndCorr->remapProbes->TileMap pV)

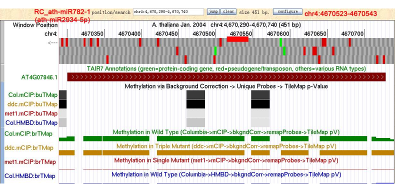
RC_ath-miR782-1
(ath-miR2934-5p)
position/search
chr4:4,670,290-4,670,740
jump
clear
size 451 bp.
configure
chr4:4670523-4670543
Window Position
A. thaliana Jan. 2004 chr4:4,670,290-4,670,740 (451 bp)
chr4:
4670350
4670400
4670450
4670500
4670550
4670600
4670650
4670700
TAIR7 Annotations (green=protein-coding gene, red=pseudogene/transposon, others=various RNA types)
AT4G07846.1
Methylation via Background Correction -> Unique Probes -> TileMap p-Value
Col.mCIP:buTMap
ddc.mCIP:buTMap
met1.mCIP:buTMap
Col.HMBD:buTMap
Col.mCIP:brTMap
Methylation in Wild Type (Columbia->mCIP->bkgndCorr->remapProbes->TileMap pV)
ddc.mCIP:brTMap
Methylation in Triple Mutant (ddc->mCIP->bkgndCorr->remapProbes->TileMap pV)
met1.mCIP:brTMap
Methylation in Single Mutant (met1->mCIP->bkgndCorr->remapProbes->TileMap pV)
Col.HMBD:brTMap
Methylation in Wild Type (Columbia->HMBD->bkgndCorr->remapProbes->TileMap pV)

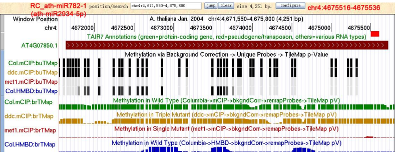

RC_ath-miR782-1
(ath-miR2934-5p)
position/search
chr4:4,671,550-4,675,800
jump
clear
size 4,251 bp.
configure
chr4:4675516-4675536
Window Position
A. thaliana Jan. 2004 chr4:4,671,550-4,675,800 (4,251 bp)
chr4:
4672000
4672500
4673000
4673500
4674000
4674500
4675000
4675500
TAIR7 Annotations (green=protein-coding gene, red=pseudogene/transposon, others=various RNA types)
AT4G07850.1
Methylation via Background Correction -> Unique Probes -> TileMap p-Value
Col.mCIP:buTMap
ddc.mCIP:buTMap
met1.mCIP:buTMap
Col.HMBD:buTMap
Col.mCIP:brTMap
Methylation in Wild Type (Columbia->mCIP->bkgndCorr->remapProbes->TileMap pV)
ddc.mCIP:brTMap
Methylation in Triple Mutant (ddc->mCIP->bkgndCorr->remapProbes->TileMap pV)
met1.mCIP:brTMap
Methylation in Single Mutant (met1->mCIP->bkgndCorr->remapProbes->TileMap pV)
Col.HMBD:brTMap
Methylation in Wild Type (Columbia->HMBD->bkgndCorr->remapProbes->TileMap pV)

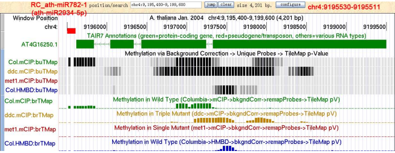
RC_ath-miR782-1
(ath-miR2934-5p)
position/search chr4:9,195,400-9,199,600
jump
clear
size 4,201 bp.
configure
chr4:9195530-9195511
Window Position
A. thaliana Jan. 2004 chr4:9,195,400-9,199,600 (4,201 bp)
chr4:
9196000
9196500
9197000
9197500
9198000
9198500
9199000
9199500
TAIR7 Annotations (green=protein-coding gene, red=pseudogene/transposon, others=various RNA types)
AT4G16250.1
Methylation via Background Correction -> Unique Probes -> TileMap p-Value
Col.mCIP:buTMap
ddc.mCIP:buTMap
met1.mCIP:buTMap
Col.HMBD:buTMap
Col.mCIP:brTMap
Methylation in Wild Type (Columbia->mCIP->bkgndCorr->remapProbes->TileMap pV)
ddc.mCIP:brTMap
Methylation in Triple Mutant (ddc->mCIP->bkgndCorr->remapProbes->TileMap pV)
met1.mCIP:brTMap
Methylation in Single Mutant (met1->mCIP->bkgndCorr->remapProbes->TileMap pV)
Col.HMBD:brTMap
Methylation in Wild Type (Columbia->HMBD->bkgndCorr->remapProbes->TileMap pV)

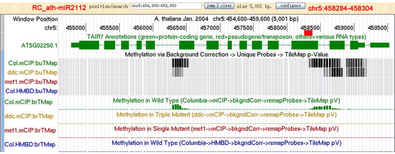
RC_ath-miR2112
position/search chr5:454,600-459,600
jump
clear
size 5,001 bp.
configure
chr5:458284-458304
Window Position
A. thaliana Jan. 2004 chr5:454,600-459,600 (5,001 bp)
chr5:
455000
455500
456000
456500
457000
457500
458000
458500
459000
459500
TAIR7 Annotations (green=protein-coding gene, red=pseudogene/transposon, others=various RNA types)
AT5G02250.1
Methylation via Background Correction -> Unique Probes -> TileMap p-Value
Col.mCIP:buTMap
ddc.mCIP:buTMap
met1.mCIP:buTMap
Col.HMBD:buTMap
Col.mCIP:brTMap
Methylation in Wild Type (Columbia->mCIP->bkgndCorr->remapProbes->TileMap pV)
ddc.mCIP:brTMap
Methylation in Triple Mutant (ddc->mCIP->bkgndCorr->remapProbes->TileMap pV)
met1.mCIP:brTMap
Methylation in Single Mutant (met1->mCIP->bkgndCorr->remapProbes->TileMap pV)
Col.HMBD:brTMap
Methylation in Wild Type (Columbia->HMBD->bkgndCorr->remapProbes->TileMap pV)

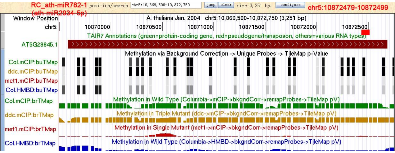

RC_ath-miR782-1
(ath-miR2934-5p)
position/search chr5:10,869,500-10,872,750
jump
clear
size 3,251 bp.
configure
chr5:10872479-10872499
Window Position
A. thaliana Jan. 2004
chr5:10,869,500-10,872,750 (3,251 bp)
chr5:
10870000
10870500
10871000
10871500
10872000
10872500
TAIR7 Annotations (green=protein-coding gene, red=pseudogene/transposon, others=various RNA types)
AT5G28845.1
Methylation via Background Correction -> Unique Probes -> TileMap p-Value
Col.mCIP:buTMap
ddc.mCIP:buTMap
met1.mCIP:buTMap
Col.HMBD:buTMap
Col.mCIP:brTMap
Methylation in Wild Type (Columbia->mCIP->bkgndCorr->remapProbes->TileMap pV)
ddc.mCIP:brTMap
Methylation in Triple Mutant (ddc->mCIP->bkgndCorr->remapProbes->TileMap pV)
met1.mCIP:brTMap
Methylation in Single Mutant (met1->mCIP->bkgndCorr->remapProbes->TileMap pV)
Col.HMBD:brTMap
Methylation in Wild Type (Columbia->HMBD->bkgndCorr->remapProbes->TileMap pV)

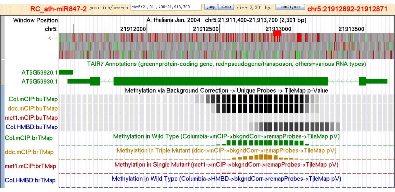

RC_ath-miR847-2
position/search chr5:21,911,400-21,913,700
jump
clear
size 2,301 bp.
configure
chr5:21912892-21912871
Window Position
A. thaliana Jan. 2004 chr5:21,911,400-21,913,700 (2,301 bp)
chr5:
21912000
21912500
21913000
21913500
TAIR7 Annotations (green=protein-coding gene, red=pseudogene/transposon, others=various RNA types)
AT5G53920.1
AT5G53930.1
Methylation via Background Correction -> Unique Probes -> TileMap p-Value
Col.mCIP:buTMap
ddc.mCIP:buTMap
met1.mCIP:buTMap
Col.HMBD:buTMap
Col.mCIP:brTMap
Methylation in Wild Type (Columbia->mCIP->bkgndCorr->remapProbes->TileMap pV)
ddc.mCIP:brTMap
Methylation in Triple Mutant (ddc->mCIP->bkgndCorr->remapProbes->TileMap pV)
met1.mCIP:brTMap
Methylation in Single Mutant (met1->mCIP->bkgndCorr->remapProbes->TileMap pV)
Col.HMBD:brTMap
Methylation in Wild Type (Columbia->HMBD->bkgndCorr->remapProbes->TileMap pV)
