# Supplementary material for: Genome-Wide Identification of Reverse Complementary microRNA Genes in Plants
Source: PLoS One. 2012 Oct 23;7(10):e46991. doi: 10.1371/journal.pone.0046991 (PMC3479107; doi:10.1371/journal.pone.0046991)
Supplement: Figure S6 — RC-miRNA-guided DNA methylation surrounding their target recognition sites in rice. (PDF) [file pone.0046991.s006.pdf]

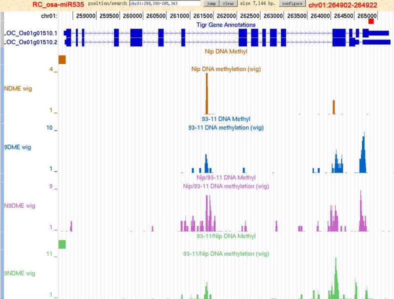

RC_osa-miR535
position/search chr01:258,200-265,343
jump
clear
size 7,144 bp.
configure
chr01:264902-264922
chr01:
259000
259500
260000
260500
261000
261500
262000
262500
263000
263500
264000
264500
265000
Tigr Gene Annotations
_OC_Os01g01510.1
_OC_Os01g01510.2
Nip DNA Methyl
Nip DNA methylation (wig)
4
NDME wig
1
93-11 DNA Methyl
93-11 DNA methylation (wig)
10
9DME wig
1
Nip/93-11 DNA Methyl
Nip/93-11 DNA methylation (wig)
9
N9DME wig
1
93-11/Nip DNA Methyl
93-11/Nip DNA methylation (wig)
11
9NDME wig
1

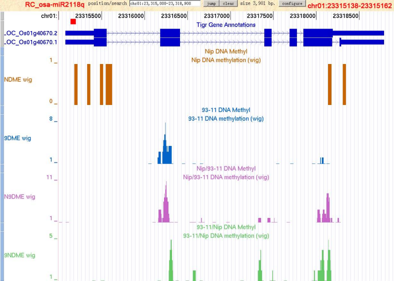

RC_osa-miR2118q
position/search
chr01:23,315,000-23,318,900
jump
clear
size 3,901 bp.
configure
chr01:23315138-23315162
chr01:
23315500
23316000
23316500
23317000
23317500
23318000
23318500
Tigr Gene Annotations
_OC_Os01g40670.2
_OC_Os01g40670.1
Nip DNA Methyl
Nip DNA methylation (wig)
NDME wig
93-11 DNA Methyl
93-11 DNA methylation (wig)
9DME wig
Nip/93-11 DNA Methyl
Nip/93-11 DNA methylation (wig)
N9DME wig
93-11/Nip DNA Methyl
93-11/Nip DNA methylation (wig)
9NDME wig

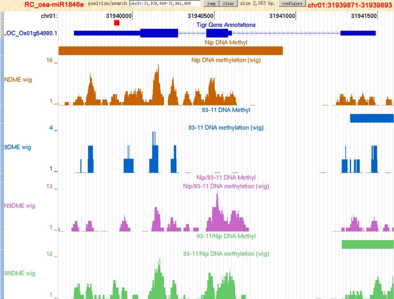
RC_osa-miR1846e
position/search
chr01:31,939,548-31,941,600
jump
clear
size 2,053 bp.
configure
chr01:31939871-31939893
chr01:
31940000
31940500
31941000
31941500
Tigr Gene Annotations
LOC_Os01g54980.1
Nip DNA Methyl
16
Nip DNA methylation (wig)
NDME wig
1
93-11 DNA Methyl
4
93-11 DNA methylation (wig)
9DME wig
1
Nip/93-11 DNA Methyl
13
Nip/93-11 DNA methylation (wig)
N9DME wig
1
93-11/Nip DNA Methyl
12
93-11/Nip DNA methylation (wig)
9NDME wig
1

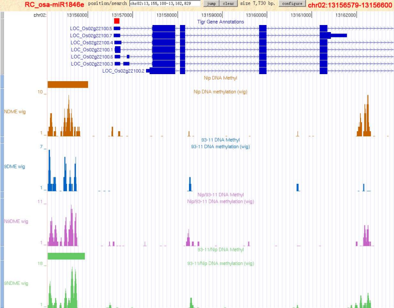

RC_osa-miR1846e
position/search
chr02:13,155,100-13,162,829
jump
clear
size 7,730 bp.
configure
chr02:13156579-13156600
chr02:
13156000
13157000
13158000
13159000
13160000
13161000
13162000
Tigr Gene Annotations
LOC_Os02g22100.5
LOC_Os02g22100.7
LOC_Os02g22100.4
LOC_Os02g22100.1
LOC_Os02g22100.6
LOC_Os02g22100.3
LOC_Os02g22100.2
Nip DNA Methyl
Nip DNA methylation (wig)
NDME wig
93-11 DNA Methyl
93-11 DNA methylation (wig)
9DME wig
Nip/93-11 DNA Methyl
Nip/93-11 DNA methylation (wig)
N9DME wig
93-11/Nip DNA Methyl
93-11/Nip DNA methylation (wig)
9NDME wig

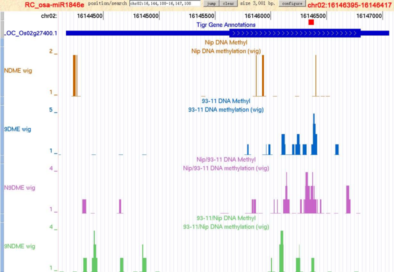

RC_osa-miR1846e
position/search
chr02:16,144,100-16,147,100
jump
clear
size 3,001 bp.
configure
chr02:16146395-16146417
chr02:
16144500
16145000
16145500
16146000
16146500
16147000
Tigr Gene Annotations
LOC_Os02g27400.1
Nip DNA Methyl
Nip DNA methylation (wig)
NDME wig
93-11 DNA Methyl
93-11 DNA methylation (wig)
9DME wig
Nip/93-11 DNA Methyl
Nip/93-11 DNA methylation (wig)
N9DME wig
93-11/Nip DNA Methyl
93-11/Nip DNA methylation (wig)
9NDME wig

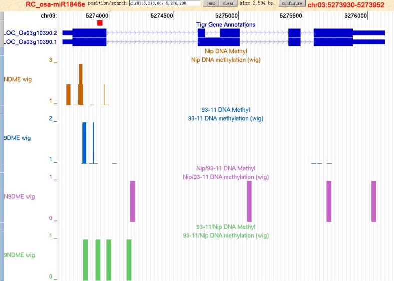

RC_osa-miR1846e
position/search
chr03:5,273,607-5,276,200
jump
clear
size 2,594 bp.
configure
chr03:5273930-5273952
chr03:
5274000
5274500
5275000
5275500
5276000
Tigr Gene Annotations
_OC_Os03g10390.2
_OC_Os03g10390.1
Nip DNA Methyl
Nip DNA methylation (wig)
NDME wig
3
1
93-11 DNA Methyl
93-11 DNA methylation (wig)
9DME wig
2
1
Nip/93-11 DNA Methyl
Nip/93-11 DNA methylation (wig)
N9DME wig
1
0
93-11/Nip DNA Methyl
93-11/Nip DNA methylation (wig)
9NDME wig
1
0

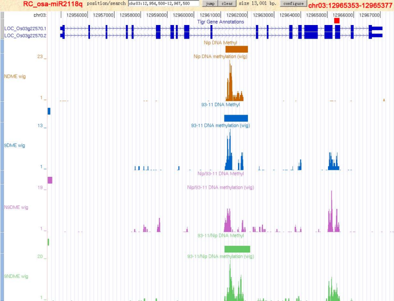

RC_osa-miR2118q
position/search chr03:12,954,500-12,967,500
jump
clear
size 13,001 bp.
configure
chr03:12965353-12965377
chr03:
12956000
12957000
12958000
12959000
12960000
12961000
12962000
12963000
12964000
12965000
12966000
12967000
Tigr Gene Annotations
LOC_Os03g22570.1
LOC_Os03g22570.2
Nip DNA Methyl
Nip DNA methylation (wig)
23
NDME wig
1
93-11 DNA Methyl
93-11 DNA methylation (wig)
13
9DME wig
1
Nip/93-11 DNA Methyl
Nip/93-11 DNA methylation (wig)
19
N9DME wig
1
93-11/Nip DNA Methyl
93-11/Nip DNA methylation (wig)
20
9NDME wig
1

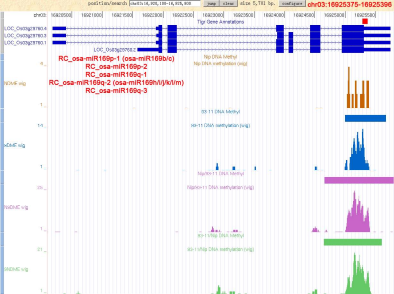
position/search
jump
clear
size 5,701 bp.
configure
chr03:16925375-16925396
chr03:
16920500
16921000
16921500
16922000
16922500
16923000
16923500
16924000
16924500
16925000
16925500
Tigr Gene Annotations
LOC_Os03g29760.4
LOC_Os03g29760.3
LOC_Os03g29760.1
LOC_Os03g29760.2
RC_osa-miR169p-1 (osa-miR169b/c)
RC_osa-miR169p-2
RC_osa-miR169q-1
RC_osa-miR169q-2 (osa-miR169h/i/j/k/l/m)
RC_osa-miR169q-3
Nip DNA Methyl
Nip DNA methylation (wig)
NDME wig
93-11 DNA Methyl
93-11 DNA methylation (wig)
9DME wig
Nip/93-11 DNA Methyl
Nip/93-11 DNA methylation (wig)
N9DME wig
93-11/Nip DNA Methyl
93-11/Nip DNA methylation (wig)
9NDME wig

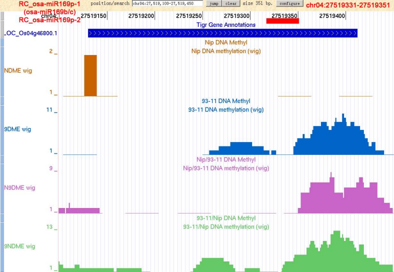
RC_osa-miR169p-1
(osa-miR169b/c)
RC_osa-miR169p-2
position/search
chr04:27,519,100-27,519,450
jump
clear
size 351 bp.
configure
chr04:27519331-27519351
chr04:
27519150
27519200
27519250
27519300
27519350
27519400
Tigr Gene Annotations
LOC_Os04g46800.1
Nip DNA Methyl
Nip DNA methylation (wig)
NDME wig
93-11 DNA Methyl
93-11 DNA methylation (wig)
9DME wig
Nip/93-11 DNA Methyl
Nip/93-11 DNA methylation (wig)
N9DME wig
93-11/Nip DNA Methyl
93-11/Nip DNA methylation (wig)
9NDME wig

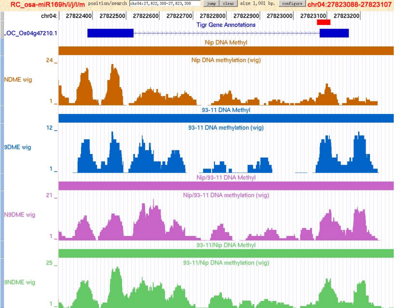

RC_osa-miR169h/i/j/l/m
chr04:27823088-27823107
chr04:
27822400
27822500
27822600
27822700
27822800
27822900
27823000
27823100
27823200
Tigr Gene Annotations
LOC_Os04g47210.1
Nip DNA Methyl
Nip DNA methylation (wig)
NDME wig
93-11 DNA Methyl
93-11 DNA methylation (wig)
9DME wig
Nip/93-11 DNA Methyl
Nip/93-11 DNA methylation (wig)
N9DME wig
93-11/Nip DNA Methyl
93-11/Nip DNA methylation (wig)
9NDME wig

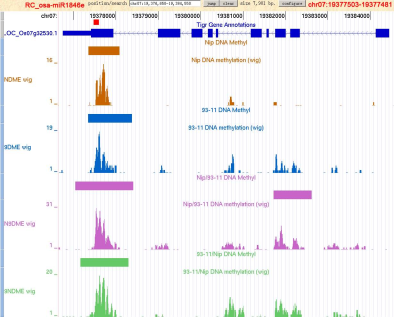
RC_osa-miR1846e
position/search
chr07:19,376,650-19,384,550
jump
clear
size 7,901 bp.
configure
chr07:19377503-19377481
chr07:
19378000
19379000
19380000
19381000
19382000
19383000
19384000
Tigr Gene Annotations
LOC_Os07g32530.1
Nip DNA Methyl
16
Nip DNA methylation (wig)
NDME wig
1
93-11 DNA Methyl
19
93-11 DNA methylation (wig)
9DME wig
1
Nip/93-11 DNA Methyl
31
Nip/93-11 DNA methylation (wig)
N9DME wig
1
93-11/Nip DNA Methyl
20
93-11/Nip DNA methylation (wig)
9NDME wig
1

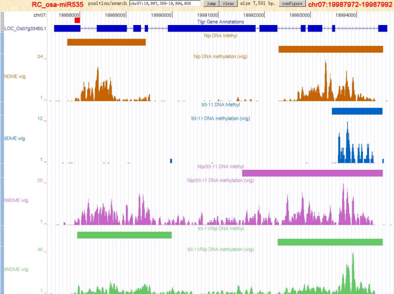
RC_osa-miR535
position/search
chr07:19,987,300-19,994,800
jump
clear
size 7,501 bp.
configure
chr07:19987972-19987992
chr07:
19988000
19989000
19990000
19991000
19992000
19993000
19994000
LOC_Os07g33450.1
Tigr Gene Annotations
Nip DNA Methyl
34
Nip DNA methylation (wig)
NDME wig
1
93-11 DNA Methyl
12
93-11 DNA methylation (wig)
9DME wig
1
Nip/93-11 DNA Methyl
20
Nip/93-11 DNA methylation (wig)
N9DME wig
1
93-11/Nip DNA Methyl
40
93-11/Nip DNA methylation (wig)
9NDME wig
1

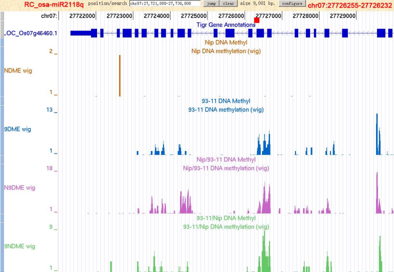

RC_osa-miR2118q
position/search
chr07:27,721,000-27,730,000
jump
clear
size 9,001 bp.
configure
chr07:27726255-27726232
chr07:
27722000
27723000
27724000
27725000
27726000
27727000
27728000
27729000
Tigr Gene Annotations
LOC_Os07g46460.1
Nip DNA Methyl
Nip DNA methylation (wig)
2 _
NDME wig
1 _
93-11 DNA Methyl
93-11 DNA methylation (wig)
13 _
9DME wig
1 _
Nip/93-11 DNA Methyl
Nip/93-11 DNA methylation (wig)
18 _
N9DME wig
1 _
93-11/Nip DNA Methyl
93-11/Nip DNA methylation (wig)
9 _
9NDME wig
1 _

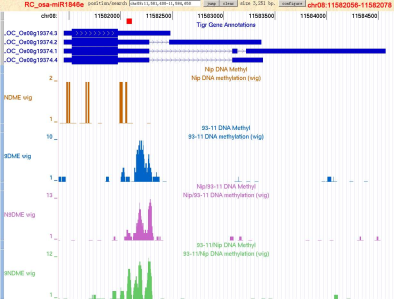

RC_osa-miR1846e
position/search
chr08:11,581,400-11,584,650
jump
clear
size 3,251 bp.
configure
chr08:11582056-11582078
chr08:
11582000
11582500
11583000
11583500
11584000
11584500
Tigr Gene Annotations
_OC_Os08g19374.3
_OC_Os08g19374.2
_OC_Os08g19374.1
_OC_Os08g19374.4
Nip DNA Methyl
Nip DNA methylation (wig)
NDME wig
2
1
93-11 DNA Methyl
93-11 DNA methylation (wig)
9DME wig
10
1
Nip/93-11 DNA Methyl
Nip/93-11 DNA methylation (wig)
N9DME wig
13
1
93-11/Nip DNA Methyl
93-11/Nip DNA methylation (wig)
9NDME wig
12
1

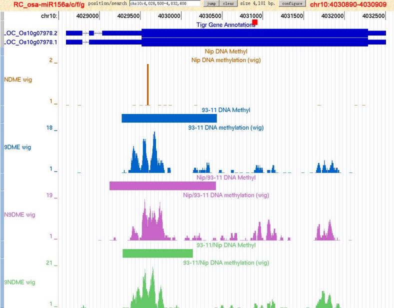

RC_osa-miR156a/c/f/g
position/search
chr10:4,028,500-4,032,600
jump
clear
size 4,101 bp.
configure
chr10:4030890-4030909
chr10:
4029000
4029500
4030000
4030500
4031000
4031500
4032000
4032500
Tigr Gene Annotations
LOC_Os10g07978.2
LOC_Os10g07978.1
Nip DNA Methyl
Nip DNA methylation (wig)
2
NDME wig
1
93-11 DNA Methyl
93-11 DNA methylation (wig)
18
9DME wig
1
Nip/93-11 DNA Methyl
Nip/93-11 DNA methylation (wig)
19
N9DME wig
1
93-11/Nip DNA Methyl
93-11/Nip DNA methylation (wig)
21
9NDME wig
1

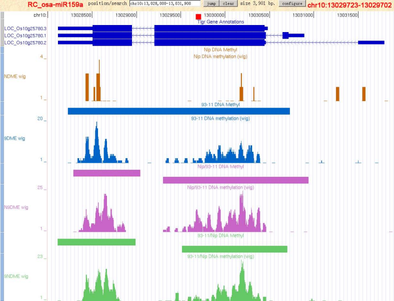
RC_osa-miR159a
position/search
chr10:13,028,000-13,031,900
jump
clear
size 3,901 bp.
configure
chr10:13029723-13029702
chr10:
13028500
13029000
13029500
13030000
13030500
13031000
13031500
Tigr Gene Annotations
LOC_Os10g25780.3
LOC_Os10g25780.1
LOC_Os10g25780.2
Nip DNA Methyl
Nip DNA methylation (wig)
NDME wig
93-11 DNA Methyl
93-11 DNA methylation (wig)
9DME wig
Nip/93-11 DNA Methyl
Nip/93-11 DNA methylation (wig)
N9DME wig
93-11/Nip DNA Methyl
93-11/Nip DNA methylation (wig)
9NDME wig

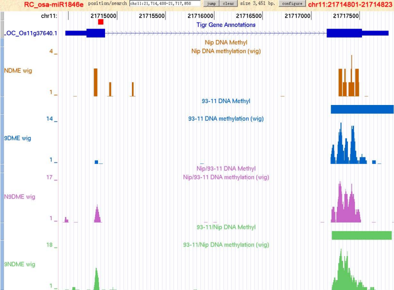
RC_osa-miR1846e
position/search chr11:21,714,400-21,717,850
jump
clear
size 3,451 bp.
configure
chr11:21714801-21714823
chr11:
21715000
21715500
21716000
21716500
21717000
21717500
Tigr Gene Annotations
LOC_Os11g37640.1
Nip DNA Methyl
Nip DNA methylation (wig)
NDME wig
93-11 DNA Methyl
93-11 DNA methylation (wig)
9DME wig
Nip/93-11 DNA Methyl
Nip/93-11 DNA methylation (wig)
N9DME wig
93-11/Nip DNA Methyl
93-11/Nip DNA methylation (wig)
9NDME wig

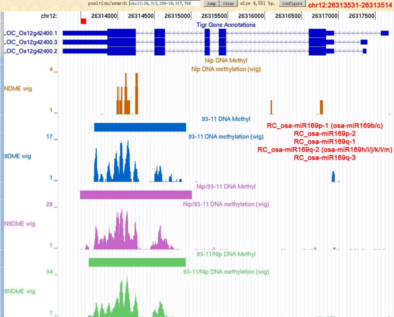

position/search chr12:26,313,200-26,317,750
jump
clear
size 4,551 bp.
configure
chr12:26313531-26313514
chr12:
26314000
26314500
26315000
26315500
26316000
26316500
26317000
26317500
Tigr Gene Annotations
_OC_Os12g42400.1
_OC_Os12g42400.3
_OC_Os12g42400.2
Nip DNA Methyl
Nip DNA methylation (wig)
NDME wig
93-11 DNA Methyl
93-11 DNA methylation (wig)
9DME wig
RC_osa-miR169p-1 (osa-miR169b/c)
RC_osa-miR169p-2
RC_osa-miR169q-1
RC_osa-miR169q-2 (osa-miR169h/i/j/k/l/m)
RC_osa-miR169q-3
Nip/93-11 DNA Methyl
Nip/93-11 DNA methylation (wig)
N9DME wig
93-11/Nip DNA Methyl
93-11/Nip DNA methylation (wig)
9NDME wig
