# Supplementary material for: Genome-Wide Identification of Reverse Complementary microRNA Genes in Plants
Source: PLoS One. 2012 Oct 23;7(10):e46991. doi: 10.1371/journal.pone.0046991 (PMC3479107; doi:10.1371/journal.pone.0046991)
Supplement: Table S1 — Plant small RNA high-throughput sequencing data sets used in this study. (PDF) [file pone.0046991.s007.pdf]

Table S1 Plant small RNA high-throughput sequencing data sets used in this study

Species	Data sets	Sources
Arabidopsis lyrata	GSM518389; GSM518390; GSM518391; GSM518392; GSM518429; GSM518430; GSM518431	GEO[a]
Arabidopsis thaliana	GSM118372; GSM118373; GSM118374; GSM118375; GSM120717; GSM121453; GSM121454; GSM121455; GSM121456; GSM121457; GSM149079; GSM149080; GSM149081; GSM154336; GSM154361; GSM154362; GSM154363; GSM154364; GSM154365; GSM154367; GSM154368; GSM154370; GSM154375; GSM154376; GSM154377; GSM253622; GSM253623; GSM253624; GSM253625; GSM257235; GSM257236; GSM257237; GSM304282; GSM304283; GSM304284; GSM304285; GSM338557; GSM342999; GSM343000; GSM343001; GSM343002; GSM343004; GSM343005; GSM366865; GSM366866; GSM366867; GSM366868; GSM366869; GSM366870; GSM442932; GSM442933; GSM442934; GSM442935; GSM456945; GSM518432; GSM642335; GSM642336; GSM642337; GSM642338	GEO[a]
Carica papaya	CPA1 Leaves_trimmed; CPA2 Flower_trimmed	CSPSR[b]
Citrus sinensis	CSI1 Leaves_trimmed; CSI2 Flower_trimmed; CSI3 Fruit_trimmed	CSPSR[b]
Glycine max	GSM769282; GSM769284; GSM769285; GSM825573; GSM852280; GSM852281	GEO[a]
Gossypium arboretum	GAR1 Leaves_trimmed; GAR2 Flower_trimmed; GAR3 Boll fibers_trimmed	CSPSR[b]
Gossypium hirsutum	GSM686014; GSM686015	GEO[a]
Hordeum vulgare	HVU1 Leaves_trimmed; HVU2 Inflorescence_trimmed	CSPSR[b]
Medicago truncatula	GSM643815; GSM643816	GEO[a]
Oryza sativa	GSM278532; GSM278533; GSM278534; GSM278535; GSM278571; GSM278572; GSM309691; GSM309692; GSM309693; GSM329296; GSM329297; GSM329298; GSM329299; GSM407071; GSM407072; GSM409313; GSM409314; GSM409315; GSM409316; GSM409317; GSM409318; GSM409319; GSM409320; GSM409321; GSM409322; GSM409323; GSM409324; GSM455962; GSM455963; GSM455964; GSM455965; GSM520634; GSM520635; GSM520636; GSM520637; GSM520638; GSM520639; GSM520640; GSM571077; GSM571078; GSM686039; GSM686040; GSM693279; GSM693280	GEO[a]
	Run1 (aerial leaf seedling); Run2 (adult stress kinase)	CSRDB[c]
Populus trichocarpa	PTR1 Leaves_trimmed; PTR2 Xylem_trimmed; PTR3 Mechanically treated xylem_trimmed	CSPSR[b]
Solanum lycopersicum	SLY1 Leaves_trimmed; SLY2 Flower_trimmed; SLY3 Fruit_trimmed	CSPSR[b]
	library1; library2; library3; library4; library5	TomFuncDB[d]
Sorghum bicolor	SBI1 Leaves_trimmed; SBI2 Flower_trimmed; SBI3 Young panicle in early grain fill_trimmed	CSPSR[b]
Triticum aestivum	TAE1 Leaves_trimmed; TAE2 Spikelet_trimmed	CSPSR[b]
Vitis vinifera	VVI1 Leaves_trimmed; VVI2 Flower_trimmed; VVI3 Fruit_trimmed	CSPSR[b]
Zea mays	GSM306487; GSM306488	GEO[a]
	run1; run2	CSRDB[c]
	ZMA1 Leaves_trimmed; ZMA2 Female inflorescence(Ears)_trimmed; ZMA3 Male inflorescence(Tassels)_trimmed	CSPSR[b]

[a]GEO (Gene Expression Omnibus), http://www.ncbi.nlm.nih.gov/geo/
[b]CSPSR (Comparative Sequencing of Plant Small RNAs), http://smallrna.udel.edu/project_data.php
[c]CSRDB (Cereal Small RNAs Database), http://sundarlab.ucdavis.edu/smrnas/
[d]TomFuncDB (Tomato Functional Genomics Database), http://ted.bti.cornell.edu/cgi-bin/TFGD/sRNA/download.cgi
