# Supplementary material for: Genome-Wide Identification of Reverse Complementary microRNA Genes in Plants
Source: PLoS One. 2012 Oct 23;7(10):e46991. doi: 10.1371/journal.pone.0046991 (PMC3479107; doi:10.1371/journal.pone.0046991)
Supplement: Table S2 — Plant degradome sequencing data sets used in this study. (PDF) [file pone.0046991.s008.pdf]

Table S2 Plant degradome sequencing data sets used in this study

Species	Data sets	Sources
Arabidopsis thaliana	GSM278333; GSM278334; GSM278335; GSM278370	GEO[a]
	AxIDT; AxIRP; AxSRP; Col; ein5l; TWF; Tx4F	NGSDBs[b]
Oryza sativa	GSM434596; GSM455938; GSM455939; GSM476257	GEO[a]
Glycine max	GSM825574	GEO[a]

[a]GEO (Gene Expression Omnibus), http://www.ncbi.nlm.nih.gov/geo/
[b]NGSDBs (Next-Gen Sequence Databases), http://mpss.udel.edu/
