# Supplementary material for: Genome-Wide Identification of Reverse Complementary microRNA Genes in Plants
Source: PLoS One. 2012 Oct 23;7(10):e46991. doi: 10.1371/journal.pone.0046991 (PMC3479107; doi:10.1371/journal.pone.0046991)
Supplement: Table S7 — Comparison of the accumulation levels between RC-microRNAs and the corresponding microRNAs in the sixteen plant species (only the RC-miRNAs with accumulation levels higher than 20 RPM were included). (PDF) [file pone.0046991.s013.pdf]

**Table S7.** Comparison of the accumulation levels between RC-microRNAs and the corresponding microRNAs in the sixteen plant species (only the RC-miRNAs with accumulation levels higher than 20 RPM were included).

| Species | RC-miRNA | Total read count (RPM)[1] | Mature miRNA | Total read count (RPM)[1] |
|---|---|---|---|---|
| *Arabidopsis thaliana* | RC-ath-miR391 | 420.68 | ath-miR391 | 14644.93 |
| | RC-ath-miR781 | 637.53 | ath-miR781 | 637.53 |
| | RC-ath-miR782-1 | 258.7 | ath-miR782 | 20.9 |
| | RC-ath-miR782-2 | 141.07 | | |
| | RC-ath-miR847-1 | 23.77 | ath-miR847 | 999.89 |
| | RC-ath-miR847-2 | 28.03 | | |
| | RC-ath-miR2112 | 37.2 | ath-miR2112-5p | 169.31 |
| | | | ath-miR2112-3p | 176.84 |
| *Oryza sativa* | RC-osa-miR156a | 1906.13 | osa-miR156a | 387568.35 |
| | RC-osa-miR156c | 1906.13 | osa-miR156c | 387568.35 |
| | RC-osa-miR156f | 1906.13 | osa-miR156f | 387568.35 |
| | RC-osa-miR156g | 1906.13 | osa-miR156g | 387568.35 |
| | RC-osa-miR159a | 245.86 | osa-miR159a.1 | 1125494.86 |
| | | | osa-miR159a.2 | 301.81 |
| | RC-osa-miR167d | 491.51 | osa-miR167d | 39776.46 |
| | RC-osa-miR167f | 491.51 | osa-miR167f | 39776.46 |
| | RC-osa-miR169b | 591.94 | osa-miR169b | 31020.69 |
| | RC-osa-miR169c | 591.94 | osa-miR169c | 31020.69 |
| | RC-osa-miR169h | 2286.81 | osa-miR169h | 222963.27 |
| | RC-osa-miR169i | 2286.81 | osa-miR169i | 222963.27 |
| | RC-osa-miR169j | 2286.81 | osa-miR169j | 222963.27 |
| | RC-osa-miR169l | 2286.81 | osa-miR169l | 222963.27 |
| | RC-osa-miR169m | 2286.81 | osa-miR169m | 222963.27 |
| | RC-osa-miR169p-1 | 31020.69 | osa-miR169p | 0 |
| | RC-osa-miR169p-2 | 1044.47 | | |
| | RC-osa-miR169q-1 | 1469.93 | osa-miR169q | 0 |
| | RC-osa-miR169q-2 | 222963.27 | | |
| | RC-osa-miR169q-3 | 1151.92 | | |
| | RC-osa-miR535 | 76.88 | osa-miR535 | 6618.92 |
| | RC-osa-miR1846e | 117.1 | osa-miR1846e | 0 |
| | RC-osa-miR1857 | 209.12 | osa-miR1857-5p | 26.99 |
| | | | osa-miR1857-3p | 391.89 |
| | RC-osa-miR2118q | 81.46 | osa-miR2118q | 84.34 |
| *Arabidopsis lyrata* | RC-aly-miR3443 | 79.27 | aly-miR3443 | 170.83 |
| | | | aly-miR3443* | 0.5 |
| *Medicago truncatula* | RC-mtr-miR2592o-1 | 33.43 | mtr-miR2592o | 5.56 |
| | RC-mtr-miR2592o-2 | 29.47 | | |
| | RC-mtr-miR2592q-1 | 33.43 | mtr-miR2592q | 5.56 |
| | RC-mtr-miR2592q-2 | 29.47 | | |
| | RC-mtr-miR2592r-1 | 33.43 | mtr-miR2592r | 5.56 |
| | RC-mtr-miR2592r-2 | 29.47 | | |

| | | | | |
|---|---|---|---|---|
| ***Populus trichocarpa*** | RC-ptc-miR396c | 238.77 | ptc-miR396c | 760.55 |
| ***Triticum aestivum*** | RC-tae-miR1120-2 | 28.26 | tae-miR1120 | 0 |
| ***Vitis vinifera*** | RC-vvi-miR172a-1 | 2154.88 | vvi-miR172a | 0 |
| | RC-vvi-miR172a-2 | 90.84 | | |
| | RC-vvi-miR172b | 2154.88 | vvi-miR172b | 0 |
| ***Zea mays*** | RC-zma-miR169d | 18173.14 | zma-miR169d | 0 |
| | RC-zma-miR169e | 18173.14 | zma-miR169e | 0 |
| ***Glycine max*** | RC_gma-MIR4413 | 423.42 | gma-miR4413 | 2.84 |
| | RC_gma-MIR4412 | 20.9 | gma-miR4412 | 7.39 |
| | RC_gma-MIR2109-1 | 978.22 | gma-miR2109 | 7.42 |
| | RC_gma-MIR2109-2 | 166 | | |
| | RC_gma-MIR1520f | 31.42 | gma-miR1520f | 5.27 |
| | RC_gma-MIR1520k | 31.42 | gma-miR1520k | 11.08 |
| | RC_gma-MIR482a | 153.35 | gma-miR482a-5p | 21.83 |
| | | | gma-miR482a-3p | 0 |
| | RC_gma-MIR167e | 914.6 | gma-miR167e | 4102.05 |
| | RC_gma-MIR167f | 914.6 | gma-miR167f | 4102.05 |

1: For one microRNA (miRNA), the “Total read count” was calculated by summing the normalized read counts (in RPM, reads per million) of this miRNA in all the analyzed small RNA high-throughput sequencing data sets.
